# Supplementary material for: A convolutional neural network for the prediction and forward design of ribozyme-based gene-control elements
Source: eLife. 2021 Apr 16;10:e59697. doi: 10.7554/eLife.59697 (PMC8128436; doi:10.7554/eLife.59697)
Supplement: Figure 6—source data 1. [file elife-59697-fig6-data1.docx]

| **Ribozyme** | **Normalized mean GFP/mCherry (+ligand)** | **Normalized GFP/mCherry std dev (+ligand)** | **Normalized mean GFP/mCherry**  **(-ligand)** | **Normalized GFP/mCherry std dev (-ligand)** | **Activation ratio** |
| --- | --- | --- | --- | --- | --- |
| Active ribozyme control | 0.02 | 0.00 | 0.01 | 0.00 | 1.05 |
| Inactive ribozyme control | 1.00 | 0.31 | 1.00 | 0.06 | 1.00 |
| FA 1 | 0.07 | 0.04 | 0.02 | 0.01 | 2.90 |
| FA 2 | 0.07 | 0.01 | 0.04 | 0.01 | 1.52 |
| FA 3 | 0.05 | 0.00 | 0.04 | 0.00 | 1.32 |
| FA 4 | 0.03 | 0.00 | 0.03 | 0.01 | 0.85 |
| FA 5 | 0.03 | 0.01 | 0.02 | 0.00 | 1.61 |
| FA 6 | 0.09 | 0.03 | 0.04 | 0.00 | 2.20 |
| FA 7 | 0.03 | 0.00 | 0.02 | 0.00 | 1.13 |
| FA 8 | 0.06 | 0.00 | 0.03 | 0.00 | 1.76 |
| FA 9 | 0.05 | 0.01 | 0.03 | 0.00 | 1.87 |
| FA 10 | 0.06 | 0.00 | 0.06 | 0.00 | 0.96 |
| FA 11 | 0.02 | 0.00 | 0.01 | 0.00 | 1.26 |
| FA 12 | 0.04 | 0.00 | 0.02 | 0.00 | 1.85 |
| FA 13 | 0.53 | 0.09 | 0.23 | 0.06 | 2.24 |
| FA 14 | 0.05 | 0.01 | 0.05 | 0.00 | 1.03 |
| FA 15 | 0.03 | 0.00 | 0.03 | 0.00 | 1.04 |
| Tet 1 | 0.76 | 0.06 | 0.28 | 0.01 | 2.75 |
| Tet 2 | 0.77 | 0.04 | 0.28 | 0.02 | 2.76 |
| Tet 3 | 0.52 | 0.24 | 0.29 | 0.05 | 1.78 |
| Tet 4 | 0.69 | 0.01 | 0.25 | 0.01 | 2.83 |
| Tet 5 | 1.00 | 0.15 | 0.41 | 0.03 | 2.46 |
| Tet 6 | 0.77 | 0.00 | 0.26 | 0.01 | 2.96 |
| Tet 7 | 0.85 | 0.05 | 0.64 | 0.05 | 1.33 |
| Tet 8 | 0.80 | 0.04 | 0.26 | 0.02 | 3.05 |
| Tet 9 | 0.65 | 0.02 | 0.14 | 0.02 | 4.52 |
| Tet 10 | 0.62 | 0.02 | 0.11 | 0.01 | 5.84 |
| Tet 11 | 0.74 | 0.02 | 0.17 | 0.01 | 4.28 |
| Tet 12 | 0.65 | 0.02 | 0.12 | 0.01 | 5.29 |
| Tet 13 | 0.76 | 0.01 | 0.41 | 0.03 | 1.85 |
| Tet 14 | 0.42 | 0.03 | 0.12 | 0.00 | 3.51 |
| Tet 15 | 0.77 | 0.08 | 0.34 | 0.02 | 2.27 |
| Theo 1 | 0.68 | 0.10 | 0.47 | 0.16 | 1.45 |
| Theo 2 | 0.75 | 0.02 | 0.66 | 0.01 | 1.13 |
| Theo 3 | 0.73 | 0.22 | 0.77 | 0.05 | 0.95 |
| Theo 4 | 0.51 | 0.09 | 0.13 | 0.04 | 4.00 |
| Theo 5 | 1.12 | 0.08 | 1.16 | 0.04 | 0.97 |
| Theo 6 | 0.81 | 0.10 | 0.77 | 0.03 | 1.05 |
| Theo 7 | 0.87 | 0.07 | 0.62 | 0.03 | 1.42 |
| Theo 8 | 1.15 | 0.07 | 1.12 | 0.11 | 1.03 |
| Theo 9 | 1.01 | 0.01 | 0.82 | 0.10 | 1.24 |
| Theo 10 | 0.61 | 0.06 | 0.23 | 0.01 | 2.62 |
| Theo 11 | 1.05 | 0.04 | 0.83 | 0.10 | 1.27 |
| Theo 12 | 1.30 | 0.05 | 1.02 | 0.03 | 1.28 |
| Theo 13 | 1.28 | 0.05 | 1.39 | 0.06 | 0.92 |
| Theo 14 | 1.35 | 0.10 | 1.36 | 0.18 | 0.99 |
| Theo 15 | 0.73 | 0.11 | 0.18 | 0.04 | 4.19 |
| Cml 1 | 0.36 | 0.04 | 0.33 | 0.03 | 1.09 |
| Cml 2 | 0.82 | 0.13 | 0.66 | 0.07 | 1.24 |
| Cml 3 | 0.25 | 0.01 | 0.23 | 0.03 | 1.11 |
| Cml 4 | 0.31 | 0.03 | 0.30 | 0.01 | 1.07 |
| Cml 5 | 0.54 | 0.14 | 0.51 | 0.10 | 1.05 |
| Cml 6 | 0.51 | 0.06 | 0.50 | 0.06 | 1.01 |
| Cml 7 | 0.50 | 0.06 | 0.44 | 0.03 | 1.12 |
| Cml 8 | 0.31 | 0.03 | 0.28 | 0.02 | 1.11 |
| Cml 9 | 0.59 | 0.03 | 0.52 | 0.02 | 1.13 |
| Cml 10 | 0.42 | 0.08 | 0.36 | 0.01 | 1.15 |
| Cml 11 | 0.67 | 0.03 | 0.51 | 0.02 | 1.31 |
| Cml 12 | 0.56 | 0.14 | 0.51 | 0.03 | 1.11 |
| Cml 13 | 0.43 | 0.08 | 0.40 | 0.02 | 1.08 |
| Cml 14 | 0.24 | 0.01 | 0.27 | 0.05 | 0.92 |
| Cml 15 | 0.48 | 0.03 | 0.41 | 0.04 | 1.17 |
| Neo 1 | 0.95 | 0.04 | 0.48 | 0.04 | 1.96 |
| Neo 2 | 0.99 | 0.09 | 0.78 | 0.06 | 1.27 |
| Neo 3 | 0.77 | 0.16 | 0.44 | 0.07 | 1.75 |
| Neo 4 | 1.17 | 0.31 | 0.83 | 0.16 | 1.40 |
| Neo 5 | 0.80 | 0.24 | 0.57 | 0.21 | 1.41 |
| Neo 6 | 0.99 | 0.08 | 0.72 | 0.13 | 1.37 |
| Neo 7 | 0.92 | 0.19 | 0.80 | 0.20 | 1.16 |
| Neo 8 | 1.04 | 0.23 | 0.90 | 0.13 | 1.15 |
| Neo 9 | 0.89 | 0.11 | 0.52 | 0.02 | 1.69 |
| Neo 10 | 0.94 | 0.08 | 0.69 | 0.06 | 1.36 |
| Neo 11 | 1.01 | 0.05 | 1.10 | 0.26 | 2.46 |
| Neo 12 | 1.09 | 0.09 | 0.88 | 0.13 | 1.24 |
| Neo 13 | 1.55 | 0.14 | 1.30 | 0.14 | 1.19 |
| Neo 14 | 1.01 | 0.15 | 0.68 | 0.03 | 1.50 |
| Neo 15 | 0.94 | 0.13 | 0.56 | 0.14 | 1.69 |

**Figure 6 - Source Table 1. Gene-regulatory activity values for individual designed ribozyme switches in the presence and absence of ligand.** Activity values for each ribozyme was generated through FACS analysis on triplicate cultures for ribozymes in the presence and absence of the cognate ligand. The standard deviation for each ribozyme represents the standard deviation of the mean GFP/mCherry values for each sample in the triplicate. The activation ratio for each ribozyme is obtained by dividing the normalized mean (+ligand) value by the normalized mean (-ligand) value. The concentrations used for the induced ribozymes were 5 mM for folinic acid, 1 mM for tetracycline, 25 μg/mL for chloramphenicol, 5 mM for theophylline, and 5 mM for neomycin.
