# Supplementary material for: A convolutional neural network for the prediction and forward design of ribozyme-based gene-control elements
Source: eLife. 2021 Apr 16;10:e59697. doi: 10.7554/eLife.59697 (PMC8128436; doi:10.7554/eLife.59697)
Supplement: Supplementary file 1. — Primers are DNA oligonucleotide sequences. [file elife-59697-supp1.docx]

| **Oligo name** | **Oligo Sequence** |
| --- | --- |
| switch_GFP_AvrII_fwd | TATGGATGAATTGTACAAATAAAGCCTAGGAAACAAACAAAGCTGTCACC |
| switch_ADH1t_XhoI_rev | GCTTATTTAGAAGTGGCGCGCCCTCTCGAGTTTTTATTTTTCTTTTTGCTGTT |
| CS_1748_gaprepair_F | TGGTATTATCCATGGTATGGATGAATTGTACAAATAAAGCCTAGG |
| CS_1748_gaprepair_R | ATAAGAAATTCGCTTATTTAGAAGTGGCGCGCCC |
| CS_L1N5_F | TGGTATTATCCATGGTATGGATGAATTGTACAAATAAAGCCTAGGAAACAAACAAAGCTGTCACCGGANNNNNTCCGGTCTGATGAGTCC |
| CS_CS_L1N6_F | TGGTATTATCCATGGTATGGATGAATTGTACAAATAAAGCCTAGGAAACAAACAAAGCTGTCACCGGANNNNNNTCCGGTCTGATGAGTCC |
| CS_CS_L1N7_F | TGGTATTATCCATGGTATGGATGAATTGTACAAATAAAGCCTAGGAAACAAACAAAGCTGTCACCGGANNNNNNNTCCGGTCTGATGAGTCC |
| CS_CS_L1N8_F | TGGTATTATCCATGGTATGGATGAATTGTACAAATAAAGCCTAGGAAACAAACAAAGCTGTCACCGGANNNNNNNNTCCGGTCTGATGAGTCC |
| CS_CS_L1N9_F | TGGTATTATCCATGGTATGGATGAATTGTACAAATAAAGCCTAGGAAACAAACAAAGCTGTCACCGGANNNNNNNNNTCCGGTCTGATGAGTCC |
| CS_CS_L1N10_F | TGGTATTATCCATGGTATGGATGAATTGTACAAATAAAGCCTAGGAAACAAACAAAGCTGTCACCGGANNNNNNNNNNTCCGGTCTGATGAGTCC |
| CS_CS_L1N11_F | TGGTATTATCCATGGTATGGATGAATTGTACAAATAAAGCCTAGGAAACAAACAAAGCTGTCACCGGANNNNNNNNNNNTCCGGTCTGATGAGTCC |
| CS_CS_L1N12_F | TGGTATTATCCATGGTATGGATGAATTGTACAAATAAAGCCTAGGAAACAAACAAAGCTGTCACCGGANNNNNNNNNNNNTCCGGTCTGATGAGTCC |
| CS_CS_L1N13_F | TGGTATTATCCATGGTATGGATGAATTGTACAAATAAAGCCTAGGAAACAAACAAAGCTGTCACCGGANNNNNNNNNNNNNTCCGGTCTGATGAGTCC |
| CS_L2N5_R | ATAAGAAATTCGCTTATTTAGAAGTGGCGCGCCCTCTCGAGTTTTTATTTTTCTTTTTGCTGTTTCGTCCNNNNNGGACTCATCAGACCGGA |
| CS_CS_L2N6_R | ATAAGAAATTCGCTTATTTAGAAGTGGCGCGCCCTCTCGAGTTTTTATTTTTCTTTTTGCTGTTTCGTCCNNNNNNGGACTCATCAGACCGGA |
| CS_CS_L2N7_R | ATAAGAAATTCGCTTATTTAGAAGTGGCGCGCCCTCTCGAGTTTTTATTTTTCTTTTTGCTGTTTCGTCCNNNNNNNGGACTCATCAGACCGGA |
| CS_CS_L2N8_R | ATAAGAAATTCGCTTATTTAGAAGTGGCGCGCCCTCTCGAGTTTTTATTTTTCTTTTTGCTGTTTCGTCCNNNNNNNNGGACTCATCAGACCGGA |
| CS_CS_L2N9_R | ATAAGAAATTCGCTTATTTAGAAGTGGCGCGCCCTCTCGAGTTTTTATTTTTCTTTTTGCTGTTTCGTCCNNNNNNNNNGGACTCATCAGACCGGA |
| CS_CS_L2N10_R | ATAAGAAATTCGCTTATTTAGAAGTGGCGCGCCCTCTCGAGTTTTTATTTTTCTTTTTGCTGTTTCGTCCNNNNNNNNNNGGACTCATCAGACCGGA |
| CS_CS_L2N11_R | ATAAGAAATTCGCTTATTTAGAAGTGGCGCGCCCTCTCGAGTTTTTATTTTTCTTTTTGCTGTTTCGTCCNNNNNNNNNNNGGACTCATCAGACCGGA |
| CS_CS_L2N12_R | ATAAGAAATTCGCTTATTTAGAAGTGGCGCGCCCTCTCGAGTTTTTATTTTTCTTTTTGCTGTTTCGTCCNNNNNNNNNNNNGGACTCATCAGACCGGA |
| CS_CS_L2N13_R | ATAAGAAATTCGCTTATTTAGAAGTGGCGCGCCCTCTCGAGTTTTTATTTTTCTTTTTGCTGTTTCGTCCNNNNNNNNNNNNNGGACTCATCAGACCGGA |
