# Supplementary material for: A convolutional neural network for the prediction and forward design of ribozyme-based gene-control elements
Source: eLife. 2021 Apr 16;10:e59697. doi: 10.7554/eLife.59697 (PMC8128436; doi:10.7554/eLife.59697)
Supplement: Supplementary file 2. — Primers are DNA oligonucleotide sequences. [file elife-59697-supp2.docx]

| **Oligo name** | **Oligo sequence** |
| --- | --- |
| GFP_F | GTCCAGTCTTGTTACCAGACAACCATTACTTATCCACTCAATCTG |
| ADH1t_R | CCCTGTTATCCCTAGCGG |
| BT1334 | AATGATACGGCGACCACCGAGATCTACACTCTTTCCCTACACGACGCTCTTCCGATCTCCGGGAAACAAACAAAGCTG |
| BT1335 | AATGATACGGCGACCACCGAGATCTACACTCTTTCCCTACACGACGCTCTTCCGATCTAGATGGGAAACAAACAAAGCTG |
| BT609 | CAAGCAGAAGACGGCATACGAGATGCCTAAGTGACTGGAGTTCAGACGTGTGCTCTTCCGATCTAATTTCTTTTTGCTGTTTCGTC |
| BT610 | CAAGCAGAAGACGGCATACGAGATTGGTCAGTGACTGGAGTTCAGACGTGTGCTCTTCCGATCTATTTCTTTTTGCTGTTTCGTC |
| BT907 | CAAGCAGAAGACGGCATACGAGATATTGGCGTGACTGGAGTTCAGACGTGTGCTCTTCCGATCTCCGTTTCTTTTTGCTGTTTCGTC |
| BT908 | CAAGCAGAAGACGGCATACGAGATGATCTGGTGACTGGAGTTCAGACGTGTGCTCTTCCGATCTGCGCTTTCTTTTTGCTGTTTCGTC |
| BT909 | CAAGCAGAAGACGGCATACGAGATAAGCTAGTGACTGGAGTTCAGACGTGTGCTCTTCCGATCTTGCACATTTCTTTTTGCTGTTTCGTC |
| BT910 | CAAGCAGAAGACGGCATACGAGATTACAAGGTGACTGGAGTTCAGACGTGTGCTCTTCCGATCTGGCTACCTTTCTTTTTGCTGTTTCGTC |
| BT1275 | CAAGCAGAAGACGGCATACGAGATTTGACTGTGACTGGAGTTCAGACGTGTGCTCTTCCGATCTCAAGGGAATTTCTTTTTGCTGTTTCGTC |
| BT1460 | CAAGCAGAAGACGGCATACGAGATGGAACTGTGACTGGAGTTCAGACGTGTGCTCTTCCGATCTGACTGTTTCTTTTTGCTGTTTCGTC |
| BT1461 | CAAGCAGAAGACGGCATACGAGATTGACATGTGACTGGAGTTCAGACGTGTGCTCTTCCGATCTGATTTCTTTTTGCTGTTTCGTC |
| BT1462 | CAAGCAGAAGACGGCATACGAGATGGACGGGTGACTGGAGTTCAGACGTGTGCTCTTCCGATCTACTTTTCTTTTTGCTGTTTCGTC |
| BT235 | CAAGCAGAAGACGGCATACG |
| BT236 | AATGATACGGCGACCACCGA |
