# Supplementary material for: A convolutional neural network for the prediction and forward design of ribozyme-based gene-control elements
Source: eLife. 2021 Apr 16;10:e59697. doi: 10.7554/eLife.59697 (PMC8128436; doi:10.7554/eLife.59697)
Supplement: Supplementary file 3. — Primers are DNA oligonucleotide sequences. [file elife-59697-supp3.docx]

| **Oligo name** | **Oligo Sequence** |
| --- | --- |
| switch_GFP_AvrII_fwd | TATGGATGAATTGTACAAATAAAGCCTAGGAAACAAACAAAGCTGTCACC |
| switch_ADH1t_XhoI_rev | GCTTATTTAGAAGTGGCGCGCCCTCTCGAGTTTTTATTTTTCTTTTTGCTGTT |
| CS_1748_gaprepair_F | TGGTATTATCCATGGTATGGATGAATTGTACAAATAAAGCCTAGG |
| CS_1748_gaprepair_R | ATAAGAAATTCGCTTATTTAGAAGTGGCGCGCCC |
| FA_tert_L1_F3 | AAACAAACAAAGCTGTCACCGGTGCTTGGTACGTTATATTCAGCCGGTCTGATGAGTCC |
| FA_tert_L2_R | TTTTTATTTTTCTTTTTGCTGTTTCGTCCTGAATATAACGTACCAAGCGGACTCATCAGA |
| Theo_tert_L1_F | AAACAAACAAAGCTGTCACCGGAATACCAGCATCGTCTTGATGCCCTTGGCAGTCCGGTCTGATGAGTCC |
| Theo_tert_L2_R | TTTTTATTTTTCTTTTTGCTGTTTCGTCCCTGCCAAGGGCATCAAGACGATGCTGGTATGGACTCATCAGA |
| Tet_tert_L1_F | AAACAAACAAAGCTGTCACCGGAAAAACATACCAGATTTCGATCTGGAGAGGTGAAGAATTCGACCACCTTCCGGTCTGATGAGTCC |
| Tet_tert_L2_R | TTTTTATTTTTCTTTTTGCTGTTTCGTCCAGGTGGTCGAATTCTTCACCTCTCCAGATCGAAATCTGGTATGTTTTGGACTCATCAGA |
| Neo_tert_L1_F | AAACAAACAAAGCTGTCACCGGAGCTTGTCCTTTAATGGTCCTCCGGTCTGATGAGTCC |
| Cml_tert_L1_F | AAACAAACAAAGCTGTCACCGGAACAGTGAAAAAAGACGTGTGAATGTCACACTGAAAAAATCCGGTCTGATGAGTCC |
| Cml_tert_L2_N5_R | TTTTTATTTTTCTTTTTGCTGTTTCGTCCNNNNNGGACTCATCAGACCGGATTTTT |
| Cml_tert_L2_N6_R | TTTTTATTTTTCTTTTTGCTGTTTCGTCCNNNNNNGGACTCATCAGACCGGATTTTT |
| Cml_tert_L2_N7_R | TTTTTATTTTTCTTTTTGCTGTTTCGTCCNNNNNNNGGACTCATCAGACCGGATTTTT |
| Cml_tert_L2_N8_R | TTTTTATTTTTCTTTTTGCTGTTTCGTCCNNNNNNNNGGACTCATCAGACCGGATTTTT |
| Neo_tert_L2_N5_R | TTTTTATTTTTCTTTTTGCTGTTTCGTCCNNNNNGGACTCATCAGACCGGAGGACC |
| Neo_tert_L2_N6_R | TTTTTATTTTTCTTTTTGCTGTTTCGTCCNNNNNNGGACTCATCAGACCGGAGGACC |
| Neo_tert_L2_N7_R | TTTTTATTTTTCTTTTTGCTGTTTCGTCCNNNNNNNGGACTCATCAGACCGGAGGACC |
| Neo_tert_L2_N8_R | TTTTTATTTTTCTTTTTGCTGTTTCGTCCNNNNNNNNGGACTCATCAGACCGGAGGACC |
| Theo_tert_L2_N5_R | TTTTTATTTTTCTTTTTGCTGTTTCGTCCNNNNNGGACTCATCAGACCGGACTGCC |
| Theo_tert_L2_N6_R | TTTTTATTTTTCTTTTTGCTGTTTCGTCCNNNNNNGGACTCATCAGACCGGACTGCC |
| Theo_tert_L2_N7_R | TTTTTATTTTTCTTTTTGCTGTTTCGTCCNNNNNNNGGACTCATCAGACCGGACTGCC |
| Theo_tert_L2_N8_R | TTTTTATTTTTCTTTTTGCTGTTTCGTCCNNNNNNNNGGACTCATCAGACCGGACTGCC |
| Tet_tert_L2_N5_R | TTTTTATTTTTCTTTTTGCTGTTTCGTCCNNNNNGGACTCATCAGACCGGAAGGTG |
| Tet_tert_L2_N6_R | TTTTTATTTTTCTTTTTGCTGTTTCGTCCNNNNNNGGACTCATCAGACCGGAAGGTG |
| Tet_tert_L2_N7_R | TTTTTATTTTTCTTTTTGCTGTTTCGTCCNNNNNNNGGACTCATCAGACCGGAAGGTG |
| Tet_tert_L2_N8_R | TTTTTATTTTTCTTTTTGCTGTTTCGTCCNNNNNNNNGGACTCATCAGACCGGAAGGTG |
| FA_tert_L2_N5_R | TTTTTATTTTTCTTTTTGCTGTTTCGTCCNNNNNGGACTCATCAGACCGGCTGAAT |
| FA_tert_L2_N6_R | TTTTTATTTTTCTTTTTGCTGTTTCGTCCNNNNNNGGACTCATCAGACCGGCTGAAT |
| FA_tert_L2_N7_R | TTTTTATTTTTCTTTTTGCTGTTTCGTCCNNNNNNNGGACTCATCAGACCGGCTGAAT |
| FA_tert_L2_N8_R | TTTTTATTTTTCTTTTTGCTGTTTCGTCCNNNNNNNNGGACTCATCAGACCGGCTGAAT |
| Theo_tert_L1_N5_F | AAACAAACAAAGCTGTCACCGGANNNNNTCCGGTCTGATGAGTCCATACCAGCAT |
| Theo_tert_L1_N6_F | AAACAAACAAAGCTGTCACCGGANNNNNNTCCGGTCTGATGAGTCCATACCAGCAT |
| Theo_tert_L1_N7_F | AAACAAACAAAGCTGTCACCGGANNNNNNNTCCGGTCTGATGAGTCCATACCAGCAT |
| Theo_tert_L1_N8_F | AAACAAACAAAGCTGTCACCGGANNNNNNNNTCCGGTCTGATGAGTCCATACCAGCAT |
| Tet_tert_L1_N5_F | AAACAAACAAAGCTGTCACCGGANNNNNTCCGGTCTGATGAGTCCAAAACATACC |
| Tet_tert_L1_N6_F | AAACAAACAAAGCTGTCACCGGANNNNNNTCCGGTCTGATGAGTCCAAAACATACC |
| Tet_tert_L1_N7_F | AAACAAACAAAGCTGTCACCGGANNNNNNNTCCGGTCTGATGAGTCCAAAACATACC |
| Tet_tert_L1_N8_F | AAACAAACAAAGCTGTCACCGGANNNNNNNNTCCGGTCTGATGAGTCCAAAACATACC |
| FA_tert_L1_N5_F | AAACAAACAAAGCTGTCACCGGANNNNNTCCGGTCTGATGAGTCCGCTTGGTACG |
| FA_tert_L1_N6_F | AAACAAACAAAGCTGTCACCGGANNNNNNTCCGGTCTGATGAGTCCGCTTGGTACG |
| FA_tert_L1_N7_F | AAACAAACAAAGCTGTCACCGGANNNNNNNTCCGGTCTGATGAGTCCGCTTGGTACG |
| FA_tert_L1_N8_F | AAACAAACAAAGCTGTCACCGGANNNNNNNNTCCGGTCTGATGAGTCCGCTTGGTACG |
