# Supplementary material for: A convolutional neural network for the prediction and forward design of ribozyme-based gene-control elements
Source: eLife. 2021 Apr 16;10:e59697. doi: 10.7554/eLife.59697 (PMC8128436; doi:10.7554/eLife.59697)
Supplement: Supplementary file 4. — Primers are DNA oligonucleotide sequences. [file elife-59697-supp4.docx]

| **Oligo name** | **Oligo Sequence** |
| --- | --- |
| switch_GFP_AvrII_fwd | TATGGATGAATTGTACAAATAAAGCCTAGGAAACAAACAAAGCTGTCACC |
| switch_ADH1t_XhoI_rev | GCTTATTTAGAAGTGGCGCGCCCTCTCGAGTTTTTATTTTTCTTTTTGCTGTT |
| FA_tert_L1_F3 | AAACAAACAAAGCTGTCACCGGTGCTTGGTACGTTATATTCAGCCGGTCTGATGAGTCC |
| FA_tert_L2_R | TTTTTATTTTTCTTTTTGCTGTTTCGTCCTGAATATAACGTACCAAGCGGACTCATCAGA |
| Theo_tert_L1_F | AAACAAACAAAGCTGTCACCGGAATACCAGCATCGTCTTGATGCCCTTGGCAGTCCGGTCTGATGAGTCC |
| Theo_tert_L2_R | TTTTTATTTTTCTTTTTGCTGTTTCGTCCCTGCCAAGGGCATCAAGACGATGCTGGTATGGACTCATCAGA |
| Tet_tert_L1_F | AAACAAACAAAGCTGTCACCGGAAAAACATACCAGATTTCGATCTGGAGAGGTGAAGAATTCGACCACCTTCCGGTCTGATGAGTCC |
| Tet_tert_L2_R | TTTTTATTTTTCTTTTTGCTGTTTCGTCCAGGTGGTCGAATTCTTCACCTCTCCAGATCGAAATCTGGTATGTTTTGGACTCATCAGA |
| Neo_tert_L1_F | AAACAAACAAAGCTGTCACCGGAGCTTGTCCTTTAATGGTCCTCCGGTCTGATGAGTCC |
| Cml_tert_L1_F | AAACAAACAAAGCTGTCACCGGAACAGTGAAAAAAGACGTGTGAATGTCACACTGAAAAAATCCGGTCTGATGAGTCC |
| FA_NGS4_1_R | TTTTTATTTTTCTTTTTGCTGTTTCGTCCCTCCAGGACTCATCAGACCGGCTGAAT |
| FA_NGS4_2_R | TTTTTATTTTTCTTTTTGCTGTTTCGTCCCCCTAGGACTCATCAGACCGGCTGAAT |
| FA_NGS4_4_F | AAACAAACAAAGCTGTCACCGGATAGGAGTCCGGTCTGATGAGTCCGCTTGGTACG |
| FA_NGS4_5_F | AAACAAACAAAGCTGTCACCGGATAGGGGTCCGGTCTGATGAGTCCGCTTGGTACG |
| FA_NGS4_6_R | TTTTTATTTTTCTTTTTGCTGTTTCGTCCCCTTAGGACTCATCAGACCGGCTGAAT |
| FA_NGS4_7_F | AAACAAACAAAGCTGTCACCGGATAAGAGTCCGGTCTGATGAGTCCGCTTGGTACG |
| FA_NGS4_8_R | TTTTTATTTTTCTTTTTGCTGTTTCGTCCCCTCAGGACTCATCAGACCGGCTGAAT |
| FA_NGS4_9_R | TTTTTATTTTTCTTTTTGCTGTTTCGTCCCTCTAGGACTCATCAGACCGGCTGAAT |
| FA_NGS4_10_F | AAACAAACAAAGCTGTCACCGGATAGTAGTCCGGTCTGATGAGTCCGCTTGGTACG |
| FA_NGS4_11_F | AAACAAACAAAGCTGTCACCGGATAAGGGTCCGGTCTGATGAGTCCGCTTGGTACG |
| FA_NGS4_14_F | AAACAAACAAAGCTGTCACCGGATAGAAGTCCGGTCTGATGAGTCCGCTTGGTACG |
| FA_NGS4_15_F | AAACAAACAAAGCTGTCACCGGATATGAGTCCGGTCTGATGAGTCCGCTTGGTACG |
| Theo_NGS4_1_R | TTTTTATTTTTCTTTTTGCTGTTTCGTCCCTCCAGGACTCATCAGACCGGACTGCC |
| Theo_NGS4_2_R | TTTTTATTTTTCTTTTTGCTGTTTCGTCCCTCGAGGACTCATCAGACCGGACTGCC |
| Theo_NGS4_3_R | TTTTTATTTTTCTTTTTGCTGTTTCGTCCCCCCAGGACTCATCAGACCGGACTGCC |
| Theo_NGS4_4_R | TTTTTATTTTTCTTTTTGCTGTTTCGTCCTTCCAGGACTCATCAGACCGGACTGCC |
| Theo_NGS4_5_R | TTTTTATTTTTCTTTTTGCTGTTTCGTCCCTTCAGGACTCATCAGACCGGACTGCC |
| Theo_NGS4_6_R | TTTTTATTTTTCTTTTTGCTGTTTCGTCCCCCGAGGACTCATCAGACCGGACTGCC |
| Theo_NGS4_7_R | TTTTTATTTTTCTTTTTGCTGTTTCGTCCCTCTAGGACTCATCAGACCGGACTGCC |
| Theo_NGS4_8_R | TTTTTATTTTTCTTTTTGCTGTTTCGTCCTCCCAGGACTCATCAGACCGGACTGCC |
| Theo_NGS4_9_R | TTTTTATTTTTCTTTTTGCTGTTTCGTCCCCCTAGGACTCATCAGACCGGACTGCC |
| Theo_NGS4_10_R | TTTTTATTTTTCTTTTTGCTGTTTCGTCCTTCGAGGACTCATCAGACCGGACTGCC |
| Theo_NGS4_11_R | TTTTTATTTTTCTTTTTGCTGTTTCGTCCCCTCAGGACTCATCAGACCGGACTGCC |
| Theo_NGS4_12_R | TTTTTATTTTTCTTTTTGCTGTTTCGTCCCTTGAGGACTCATCAGACCGGACTGCC |
| Theo_NGS4_13_F | AAACAAACAAAGCTGTCACCGGAAAATGGGTCCGGTCTGATGAGTCCATACCAGCAT |
| Theo_NGS4_14_F | AAACAAACAAAGCTGTCACCGGAATATGGGTCCGGTCTGATGAGTCCATACCAGCAT |
| Theo_NGS4_15_R | TTTTTATTTTTCTTTTTGCTGTTTCGTCCTCCCTTGGACTCATCAGACCGGACTGCC |
| Tet_NGS4_1_R | TTTTTATTTTTCTTTTTGCTGTTTCGTCCTTCCTGGACTCATCAGACCGGAAGGTG |
| Tet_NGS4_2_R | TTTTTATTTTTCTTTTTGCTGTTTCGTCCTCCCTGGACTCATCAGACCGGAAGGTG |
| Tet_NGS4_3_R | TTTTTATTTTTCTTTTTGCTGTTTCGTCCCTCCTGGACTCATCAGACCGGAAGGTG |
| Tet_NGS4_4_R | TTTTTATTTTTCTTTTTGCTGTTTCGTCCTTCCCCCTGGACTCATCAGACCGGAAGGTG |
| Tet_NGS4_5_R | TTTTTATTTTTCTTTTTGCTGTTTCGTCCTTCTTGGACTCATCAGACCGGAAGGTG |
| Tet_NGS4_6_R | TTTTTATTTTTCTTTTTGCTGTTTCGTCCTCCTTGGACTCATCAGACCGGAAGGTG |
| Tet_NGS4_7_R | TTTTTATTTTTCTTTTTGCTGTTTCGTCCTTCCCTGGACTCATCAGACCGGAAGGTG |
| Tet_NGS4_8_R | TTTTTATTTTTCTTTTTGCTGTTTCGTCCTTTCTGGACTCATCAGACCGGAAGGTG |
| Tet_NGS4_9_R | TTTTTATTTTTCTTTTTGCTGTTTCGTCCTTACCCCTGGACTCATCAGACCGGAAGGTG |
| Tet_NGS4_10_R | TTTTTATTTTTCTTTTTGCTGTTTCGTCCTTCACCCTGGACTCATCAGACCGGAAGGTG |
| Tet_NGS4_11_R | TTTTTATTTTTCTTTTTGCTGTTTCGTCCTTATCCCTGGACTCATCAGACCGGAAGGTG |
| Tet_NGS4_12_R | TTTTTATTTTTCTTTTTGCTGTTTCGTCCTTTCCCCTGGACTCATCAGACCGGAAGGTG |
| Tet_NGS4_13_R | TTTTTATTTTTCTTTTTGCTGTTTCGTCCTCCACCCTGGACTCATCAGACCGGAAGGTG |
| Tet_NGS4_14_R | TTTTTATTTTTCTTTTTGCTGTTTCGTCCTTTTCCCTGGACTCATCAGACCGGAAGGTG |
| Tet_NGS4_15_R | TTTTTATTTTTCTTTTTGCTGTTTCGTCCTCACCCCTGGACTCATCAGACCGGAAGGTG |
| Cml_NGS4_1_R | TTTTTATTTTTCTTTTTGCTGTTTCGTCCCTCCAGGACTCATCAGACCGGATTTTT |
| Cml_NGS4_2_R | TTTTTATTTTTCTTTTTGCTGTTTCGTCCCTCTAGGACTCATCAGACCGGATTTTT |
| Cml_NGS4_3_R | TTTTTATTTTTCTTTTTGCTGTTTCGTCCCCCTAGGACTCATCAGACCGGATTTTT |
| Cml_NGS4_4_R | TTTTTATTTTTCTTTTTGCTGTTTCGTCCCCCCAGGACTCATCAGACCGGATTTTT |
| Cml_NGS4_5_R | TTTTTATTTTTCTTTTTGCTGTTTCGTCCCCCTTAGGACTCATCAGACCGGATTTTT |
| Cml_NGS4_6_R | TTTTTATTTTTCTTTTTGCTGTTTCGTCCCGCTAGGACTCATCAGACCGGATTTTT |
| Cml_NGS4_7_R | TTTTTATTTTTCTTTTTGCTGTTTCGTCCCCTTTAGGACTCATCAGACCGGATTTTT |
| Cml_NGS4_8_R | TTTTTATTTTTCTTTTTGCTGTTTCGTCCCCTTAGGACTCATCAGACCGGATTTTT |
| Cml_NSG4_9_R | TTTTTATTTTTCTTTTTGCTGTTTCGTCCCCCCTAGGACTCATCAGACCGGATTTTT |
| Cml_NGS4_10_R | TTTTTATTTTTCTTTTTGCTGTTTCGTCCCTTCAGGACTCATCAGACCGGATTTTT |
| Cml_NGS4_11_R | TTTTTATTTTTCTTTTTGCTGTTTCGTCCCTCGAGGACTCATCAGACCGGATTTTT |
| Cml_NGS4_12_R | TTTTTATTTTTCTTTTTGCTGTTTCGTCCCTACTAGGACTCATCAGACCGGATTTTT |
| Cml_NGS4_13_R | TTTTTATTTTTCTTTTTGCTGTTTCGTCCCCTCTAGGACTCATCAGACCGGATTTTT |
| Cml_NGS4_14_R | TTTTTATTTTTCTTTTTGCTGTTTCGTCCTTCCAGGACTCATCAGACCGGATTTTT |
| Cml_NGS4_15_R | TTTTTATTTTTCTTTTTGCTGTTTCGTCCCGCCAGGACTCATCAGACCGGATTTTT |
| Neo_NGS4_1_R | TTTTTATTTTTCTTTTTGCTGTTTCGTCCCCCCTGGACTCATCAGACCGGAGGACC |
| Neo_NGS4_2_R | TTTTTATTTTTCTTTTTGCTGTTTCGTCCTTCCGGGACTCATCAGACCGGAGGACC |
| Neo_NGS4_3_R | TTTTTATTTTTCTTTTTGCTGTTTCGTCCCTCCTGGACTCATCAGACCGGAGGACC |
| Neo_NGS4_4_R | TTTTTATTTTTCTTTTTGCTGTTTCGTCCCCCCCTGGACTCATCAGACCGGAGGACC |
| Neo_NGS4_5_R | TTTTTATTTTTCTTTTTGCTGTTTCGTCCCCACTGGACTCATCAGACCGGAGGACC |
| Neo_NGS4_6_R | TTTTTATTTTTCTTTTTGCTGTTTCGTCCCCCTTGGACTCATCAGACCGGAGGACC |
| Neo_NSG4_7_R | TTTTTATTTTTCTTTTTGCTGTTTCGTCCTTCTGGGACTCATCAGACCGGAGGACC |
| Neo_NGS4_8_R | TTTTTATTTTTCTTTTTGCTGTTTCGTCCTTACGGGACTCATCAGACCGGAGGACC |
| Neo_NGS4_9_R | TTTTTATTTTTCTTTTTGCTGTTTCGTCCCCTCTGGACTCATCAGACCGGAGGACC |
| Neo_NGS4_10_R | TTTTTATTTTTCTTTTTGCTGTTTCGTCCCCCCTTGGACTCATCAGACCGGAGGACC |
| Neo_NGS4_11_R | TTTTTATTTTTCTTTTTGCTGTTTCGTCCCCCGTGGACTCATCAGACCGGAGGACC |
| Neo_NGS4_12_R | TTTTTATTTTTCTTTTTGCTGTTTCGTCCCTCCAGGACTCATCAGACCGGAGGACC |
| Neo_NGS4_13_R | TTTTTATTTTTCTTTTTGCTGTTTCGTCCCCTCCTGGACTCATCAGACCGGAGGACC |
| Neo_NGS4_14_R | TTTTTATTTTTCTTTTTGCTGTTTCGTCCCTCTTGGACTCATCAGACCGGAGGACC |
| Neo_NGS4_15_R | TTTTTATTTTTCTTTTTGCTGTTTCGTCCCTACTGGACTCATCAGACCGGAGGACC |
