# Supplementary material for: A convolutional neural network for the prediction and forward design of ribozyme-based gene-control elements
Source: eLife. 2021 Apr 16;10:e59697. doi: 10.7554/eLife.59697 (PMC8128436; doi:10.7554/eLife.59697)
Supplement: Supplementary file 5. — Structures are laid out in dot-bracket notation. [file elife-59697-supp5.docx]

| Folinic Acid |
| --- |
| ...........(((((.((((((.....(((...))).....))))))(....).(((((...)))))...)))))................. |
| ...........(((((.((((((.....(((...))).....))))))(....).(((((...)))))...)))))................. |
| ...........(((((.((((((.....(((...))).....))))))(....).(((((...)))))...)))))................. |
| ...........(((((.((((((.....(((...))).....))))))(....).(((((...)))))...)))))................. |
| ...........(((((.((((((.....(((...))).....))))))(....).(((((...)))))...)))))................. |
| ...........(((((.((((((.....(((...))).....))))))(....).(((((...)))))...)))))................. |
| ...........(((((.((((((.....(((...))).....))))))(....).(((((...)))))...)))))................. |
| ...........(((((.((((((.....(((...))).....))))))(....).(((((...)))))...)))))................. |
| ...........(((((.((((((.....(((...))).....))))))(....).(((((...)))))...)))))................. |
| ...........(((((.((((((.....(((...))).....))))))(....).(((((...)))))...)))))................. |
| ...........(((((.((((((.....(((...))).....))))))(....).(((((...)))))...)))))................. |
| ...........(((((.((((((.....(((...))).....))))))(....).(((((...)))))...)))))................. |
| ...........(((((.((((((.....(((...))).....))))))(....).(((((...)))))...)))))................. |
| ...........(((((.((((((.....(((...))).....))))))(....).(((((...)))))...)))))................. |
| ...........(((((.((((((.....(((...))).....))))))(....).(((((...)))))...)))))................. |
| ...........(((((.((((((.....(((...))).....))))))(....).(((((...)))))...)))))................. |
| ...........(((((.((((((.....(((...))).....))))))(....).(((((...)))))...)))))................. |
| ...........(((((.((((((.....(((...))).....))))))(....).(((((...)))))...)))))................. |
| ...........(((((.((((((.....(((...))).....))))))(....).(((((...)))))...)))))................. |
| ...........(((((.((((((.....(((...))).....))))))(....).(((((...)))))...)))))................. |
| ...........(((((.((((((.....(((...))).....))))))(....).(((((...)))))...)))))................. |
| ...........(((((.((((((.....(((...))).....))))))(....).(((((...)))))...)))))................. |
| ...........(((((.((((((.....(((...))).....))))))(....).(((((...)))))...)))))................. |
| ...........(((((.((((((.....(((...))).....))))))(....).(((((...)))))...)))))................. |
| ...........(((((.((((((.....(((...))).....))))))(....).(((((...)))))...)))))................. |
| ...........(((((.((((((.....(((...))).....))))))(....).(((((...)))))...)))))................. |
| ...........(((((.((((((.....(((...))).....))))))(....).(((((...)))))...)))))................. |
| ...........(((((.((((((.....(((...))).....))))))(....).(((((...)))))...)))))................. |
| ...........(((((.((((((.....(((...))).....))))))(....).(((((...)))))...)))))................. |
| ...........(((((.((((((.....(((...))).....))))))(....).(((((...)))))...)))))................. |
| ...........(((((.((((((.....(((...))).....))))))(....).(((((...)))))...)))))................. |
| ...........(((((.((((((.....(((...))).....))))))(....).(((((...)))))...)))))................. |
| ...........(((((.((((((.....(((...))).....))))))(....).(((((...)))))...)))))................. |
| ...........(((((.((((((.....(((...))).....))))))(....).(((((...)))))...)))))................. |
| ...........(((((.((((((.....(((...))).....))))))(....).(((((...)))))...)))))................. |
| ...........(((((.((((((.....(((...))).....))))))(....).(((((...)))))...)))))................. |
| ...........(((((.((((((.....(((...))).....))))))(....).(((((...)))))...)))))................. |
| ...........(((((.((((((.....(((...))).....))))))(....).(((((...)))))...)))))................. |
| ...........(((((.((((((.....(((...))).....))))))(....).(((((...)))))...)))))................. |
| ...........(((((.((((((.....(((...))).....))))))(....).(((((...)))))...)))))................. |
| ...........(((((.((((((.....(((...))).....))))))(....).(((((...)))))...)))))................. |
| ...........(((((.((((((.....(((...))).....))))))(....).(((((...)))))...)))))................. |
| ...........(((((.((((((.....(((...))).....))))))(....).(((((...)))))...)))))................. |
| ...........(((((.((((((.....(((...))).....))))))(....).(((((...)))))...)))))................. |
| ...........(((((.((((((.....(((...))).....))))))(....).(((((...)))))...)))))................. |
| ...........(((((.((((((.....(((...))).....))))))(....).(((((...)))))...)))))................. |
| ...........(((((.((((((.....(((...))).....))))))(....).(((((...)))))...)))))................. |
| ...........(((((.((((((.....(((...))).....))))))(....).(((((...)))))...)))))................. |
| ...........(((((.((((((.....(((...))).....))))))(....).(((((...)))))...)))))................. |
| ...........(((((.((((((.....(((...))).....))))))(....).(((((...)))))...)))))................. |
| ...........(((((.((((((.....(((...))).....))))))(....).(((((...)))))...)))))................. |
| ...........(((((.((((((.....(((...))).....))))))(....).(((((...)))))...)))))................. |
| ...........(((((.((((((.....(((...))).....))))))(....).(((((...)))))...)))))................. |
| ...........(((((.((((((.....(((...))).....))))))(....).(((((...)))))...)))))................. |
| ...........(((((.((((((.....(((...))).....))))))(....).(((((...)))))...)))))................. |
| ...........(((((.((((((.....(((...))).....))))))(....).(((((...)))))...)))))................. |
| ...........(((((.((((((.....(((...))).....))))))(....).(((((...)))))...)))))................. |
| ...........(((((.((((((.....(((...))).....))))))(....).(((((...)))))...)))))................. |
| ...........(((((.((((((.....(((...))).....))))))(....).(((((...)))))...)))))................. |
| ...........(((((.((((((.....(((...))).....))))))(....).(((((...)))))...)))))................. |
| ...........(((((.((((((.....(((...))).....))))))(....).(((((...)))))...)))))................. |
| ...........(((((.((((((.....(((...))).....))))))(....).(((((...)))))...)))))................. |
| ...........(((((.((((((.....(((...))).....))))))(....).(((((...)))))...)))))................. |
| ...........(((((.((((((.....(((...))).....))))))(....).(((((...)))))...)))))................. |
| ...........(((((.((((((.....(((...))).....))))))(....).(((((...)))))...)))))................. |
| ...........(((((.((((((.....(((...))).....))))))(....).(((((...)))))...)))))................. |
| ...........(((((.((((((.....(((...))).....))))))(....).(((((...)))))...)))))................. |
| ...........(((((.((((((.....(((...))).....))))))(....).(((((...)))))...)))))................. |
| ...........(((((.((((((.....(((...))).....))))))(....).(((((...)))))...)))))................. |
| ...........(((((.((((((.....(((...))).....))))))(....).(((((...)))))...)))))................. |
| ...........(((((.((((((.....(((...))).....))))))(....).(((((...)))))...)))))................. |
| ...........(((((.((((((.....(((...))).....))))))(....).(((((...)))))...)))))................. |
| ...........(((((.((((((.....(((...))).....))))))(....).(((((...)))))...)))))................. |
| ...........(((((.((((((.....(((...))).....))))))(....).(((((...)))))...)))))................. |
| ...........(((((.((((((.....(((...))).....))))))(....).(((((...)))))...)))))................. |
| ...........(((((.((((((.....(((...))).....))))))(....).(((((...)))))...)))))................. |
| ...........(((((.((((((.....(((...))).....))))))(....).(((((...)))))...)))))................. |
| ...........(((((.((((((.....(((...))).....))))))(....).(((((...)))))...)))))................. |
| ...........(((((.((((((.....(((...))).....))))))(....).(((((...)))))...)))))................. |
| ...........(((((.((((((.....(((...))).....))))))(....).(((((...)))))...)))))................. |
| ...........(((((.((((((.....(((...))).....))))))(....).(((((...)))))...)))))................. |
| ...........(((((.((((((.....(((...))).....))))))(....).(((((...)))))...)))))................. |
| ...........(((((.((((((.....(((...))).....))))))(....).(((((...)))))...)))))................. |
| ...........(((((.((((((.....(((...))).....))))))(....).(((((...)))))...)))))................. |
| ...........(((((.((((((.....(((...))).....))))))(....).(((((...)))))...)))))................. |
| ...........(((((.((((((.....(((...))).....))))))(....).(((((...)))))...)))))................. |
| ...........(((((.((((((.....(((...))).....))))))(....).(((((...)))))...)))))................. |
| ...........(((((.((((((.....(((...))).....))))))(....).(((((...)))))...)))))................. |
| ...........(((((.((((((.....(((...))).....))))))(....).(((((...)))))...)))))................. |
| ...........(((((.((((((.....(((...))).....))))))(....).(((((...)))))...)))))................. |
| ...........(((((.((((((.....(((...))).....))))))(....).(((((...)))))...)))))................. |
| ...........(((((.((((((.....(((...))).....))))))(....).(((((...)))))...)))))................. |
| ...........(((((.((((((.....(((...))).....))))))(....).(((((...)))))...)))))................. |
| ...........(((((.((((((.....(((...))).....))))))(....).(((((...)))))...)))))................. |
| ...........(((((.((((((.....(((...))).....))))))(....).(((((...)))))...)))))................. |
| ...........(((((.((((((.....(((...))).....))))))(....).(((((...)))))...)))))................. |
| ...........(((((.((((((.....(((...))).....))))))(....).(((((...)))))...)))))................. |
| ...........(((((.((((((.....(((...))).....))))))(....).(((((...)))))...)))))................. |
| ...........(((((.((((((.....(((...))).....))))))(....).(((((...)))))...)))))................. |
| ...........(((((.((((((.....(((...))).....))))))(....).(((((...)))))...)))))................. |
| Tetracycline |
| ...........(((((.(((((.........((((((....))))))..(((((...........))))).)))))(....).((((.....))))...)))))................. |
| ...........(((((.(((((.........((((((....))))))..(((((...........))))).)))))(....).((((.....))))...)))))................. |
| ...........(((((.(((((.........((((((....))))))..(((((...........))))).)))))(....).((((.....))))...)))))................. |
| ...........(((((.(((((.........((((((....))))))..(((((...........))))).)))))(....).((((.....))))...)))))................. |
| ...........(((((.(((((.........((((((....))))))..(((((...........))))).)))))(....).((((.....))))...)))))................. |
| ...........(((((.(((((.........((((((....))))))..(((((...........))))).)))))(....).((((.....))))...)))))................. |
| ...........(((((.(((((.........((((((....))))))..(((((...........))))).)))))(....).((((.....))))...)))))................. |
| ...........(((((.(((((.........((((((....))))))..(((((...........))))).)))))(....).((((.....))))...)))))................. |
| ...........(((((.(((((.........((((((....))))))..(((((...........))))).)))))(....).((((.....))))...)))))................. |
| ...........(((((.(((((.........((((((....))))))..(((((...........))))).)))))(....).((((.....))))...)))))................. |
| ...........(((((.(((((.........((((((....))))))..(((((...........))))).)))))(....).((((.....))))...)))))................. |
| ...........(((((.(((((.........((((((....))))))..(((((...........))))).)))))(....).((((.....))))...)))))................. |
| ...........(((((.(((((.........((((((....))))))..(((((...........))))).)))))(....).((((.....))))...)))))................. |
| ...........(((((.(((((.........((((((....))))))..(((((...........))))).)))))(....).((((.....))))...)))))................. |
| ...........(((((.(((((.........((((((....))))))..(((((...........))))).)))))(....).((((.....))))...)))))................. |
| ...........(((((.(((((.........((((((....))))))..(((((...........))))).)))))(....).((((.....))))...)))))................. |
| ...........(((((.(((((.........((((((....))))))..(((((...........))))).)))))(....).((((.....))))...)))))................. |
| ...........(((((.(((((.........((((((....))))))..(((((...........))))).)))))(....).((((.....))))...)))))................. |
| ...........(((((.(((((.........((((((....))))))..(((((...........))))).)))))(....).((((.....))))...)))))................. |
| ...........(((((.(((((.........((((((....))))))..(((((...........))))).)))))(....).((((.....))))...)))))................. |
| ...........(((((.(((((.........((((((....))))))..(((((...........))))).)))))(....).((((.....))))...)))))................. |
| ...........(((((.(((((.........((((((....))))))..(((((...........))))).)))))(....).((((.....))))...)))))................. |
| ...........(((((.(((((.........((((((....))))))..(((((...........))))).)))))(....).((((.....))))...)))))................. |
| ...........(((((.(((((.........((((((....))))))..(((((...........))))).)))))(....).((((.....))))...)))))................. |
| ...........(((((.(((((.........((((((....))))))..(((((...........))))).)))))(....).((((.....))))...)))))................. |
| ...........(((((.(((((.........((((((....))))))..(((((...........))))).)))))(....).((((.....))))...)))))................. |
| ...........(((((.(((((.........((((((....))))))..(((((...........))))).)))))(....).((((.....))))...)))))................. |
| ...........(((((.(((((.........((((((....))))))..(((((...........))))).)))))(....).((((.....))))...)))))................. |
| ...........(((((.(((((.........((((((....))))))..(((((...........))))).)))))(....).((((.....))))...)))))................. |
| ...........(((((.(((((.........((((((....))))))..(((((...........))))).)))))(....).((((.....))))...)))))................. |
| ...........(((((.(((((.........((((((....))))))..(((((...........))))).)))))(....).((((.....))))...)))))................. |
| ...........(((((.(((((.........((((((....))))))..(((((...........))))).)))))(....).((((.....))))...)))))................. |
| ...........(((((.(((((.........((((((....))))))..(((((...........))))).)))))(....).((((.....))))...)))))................. |
| ...........(((((.(((((.........((((((....))))))..(((((...........))))).)))))(....).((((.....))))...)))))................. |
| ...........(((((.(((((.........((((((....))))))..(((((...........))))).)))))(....).((((.....))))...)))))................. |
| ...........(((((.(((((.........((((((....))))))..(((((...........))))).)))))(....).((((.....))))...)))))................. |
| ...........(((((.(((((.........((((((....))))))..(((((...........))))).)))))(....).((((.....))))...)))))................. |
| ...........(((((.(((((.........((((((....))))))..(((((...........))))).)))))(....).((((.....))))...)))))................. |
| ...........(((((.(((((.........((((((....))))))..(((((...........))))).)))))(....).((((.....))))...)))))................. |
| ...........(((((.(((((.........((((((....))))))..(((((...........))))).)))))(....).((((.....))))...)))))................. |
| ...........(((((.(((((.........((((((....))))))..(((((...........))))).)))))(....).((((.....))))...)))))................. |
| ...........(((((.(((((.........((((((....))))))..(((((...........))))).)))))(....).((((.....))))...)))))................. |
| ...........(((((.(((((.........((((((....))))))..(((((...........))))).)))))(....).((((.....))))...)))))................. |
| ...........(((((.(((((.........((((((....))))))..(((((...........))))).)))))(....).((((.....))))...)))))................. |
| ...........(((((.(((((.........((((((....))))))..(((((...........))))).)))))(....).((((.....))))...)))))................. |
| ...........(((((.(((((.........((((((....))))))..(((((...........))))).)))))(....).((((.....))))...)))))................. |
| ...........(((((.(((((.........((((((....))))))..(((((...........))))).)))))(....).((((.....))))...)))))................. |
| ...........(((((.(((((.........((((((....))))))..(((((...........))))).)))))(....).((((.....))))...)))))................. |
| ...........(((((.(((((.........((((((....))))))..(((((...........))))).)))))(....).((((.....))))...)))))................. |
| ...........(((((.(((((.........((((((....))))))..(((((...........))))).)))))(....).((((.....))))...)))))................. |
| ...........(((((.(((((.........((((((....))))))..(((((...........))))).)))))(....).((((.....))))...)))))................. |
| ...........(((((.(((((.........((((((....))))))..(((((...........))))).)))))(....).((((.....))))...)))))................. |
| ...........(((((.(((((.........((((((....))))))..(((((...........))))).)))))(....).((((.....))))...)))))................. |
| ...........(((((.(((((.........((((((....))))))..(((((...........))))).)))))(....).((((.....))))...)))))................. |
| ...........(((((.(((((.........((((((....))))))..(((((...........))))).)))))(....).((((.....))))...)))))................. |
| ...........(((((.(((((.........((((((....))))))..(((((...........))))).)))))(....).((((.....))))...)))))................. |
| ...........(((((.(((((.........((((((....))))))..(((((...........))))).)))))(....).((((.....))))...)))))................. |
| ...........(((((.(((((.........((((((....))))))..(((((...........))))).)))))(....).((((.....))))...)))))................. |
| ...........(((((.(((((.........((((((....))))))..(((((...........))))).)))))(....).((((.....))))...)))))................. |
| ...........(((((.(((((.........((((((....))))))..(((((...........))))).)))))(....).((((.....))))...)))))................. |
| ...........(((((.(((((.........((((((....))))))..(((((...........))))).)))))(....).((((.....))))...)))))................. |
| ...........(((((.(((((.........((((((....))))))..(((((...........))))).)))))(....).((((.....))))...)))))................. |
| ...........(((((.(((((.........((((((....))))))..(((((...........))))).)))))(....).((((.....))))...)))))................. |
| ...........(((((.(((((.........((((((....))))))..(((((...........))))).)))))(....).((((.....))))...)))))................. |
| ...........(((((.(((((.........((((((....))))))..(((((...........))))).)))))(....).((((.....))))...)))))................. |
| ...........(((((.(((((.........((((((....))))))..(((((...........))))).)))))(....).((((.....))))...)))))................. |
| ...........(((((.(((((.........((((((....))))))..(((((...........))))).)))))(....).((((.....))))...)))))................. |
| ...........(((((.(((((.........((((((....))))))..(((((...........))))).)))))(....).((((.....))))...)))))................. |
| ...........(((((.(((((.........((((((....))))))..(((((...........))))).)))))(....).((((.....))))...)))))................. |
| ...........(((((.(((((.........((((((....))))))..(((((...........))))).)))))(....).((((.....))))...)))))................. |
| ...........(((((.(((((.........((((((....))))))..(((((...........))))).)))))(....).((((.....))))...)))))................. |
| ...........(((((.(((((.........((((((....))))))..(((((...........))))).)))))(....).((((.....))))...)))))................. |
| ...........(((((.(((((.........((((((....))))))..(((((...........))))).)))))(....).((((.....))))...)))))................. |
| ...........(((((.(((((.........((((((....))))))..(((((...........))))).)))))(....).((((.....))))...)))))................. |
| ...........(((((.(((((.........((((((....))))))..(((((...........))))).)))))(....).((((.....))))...)))))................. |
| ...........(((((.(((((.........((((((....))))))..(((((...........))))).)))))(....).((((.....))))...)))))................. |
| ...........(((((.(((((.........((((((....))))))..(((((...........))))).)))))(....).((((.....))))...)))))................. |
| ...........(((((.(((((.........((((((....))))))..(((((...........))))).)))))(....).((((.....))))...)))))................. |
| ...........(((((.(((((.........((((((....))))))..(((((...........))))).)))))(....).((((.....))))...)))))................. |
| ...........(((((.(((((.........((((((....))))))..(((((...........))))).)))))(....).((((.....))))...)))))................. |
| ...........(((((.(((((.........((((((....))))))..(((((...........))))).)))))(....).((((.....))))...)))))................. |
| ...........(((((.(((((.........((((((....))))))..(((((...........))))).)))))(....).((((.....))))...)))))................. |
| ...........(((((.(((((.........((((((....))))))..(((((...........))))).)))))(....).((((.....))))...)))))................. |
| ...........(((((.(((((.........((((((....))))))..(((((...........))))).)))))(....).((((.....))))...)))))................. |
| ...........(((((.(((((.........((((((....))))))..(((((...........))))).)))))(....).((((.....))))...)))))................. |
| ...........(((((.(((((.........((((((....))))))..(((((...........))))).)))))(....).((((.....))))...)))))................. |
| ...........(((((.(((((.........((((((....))))))..(((((...........))))).)))))(....).((((.....))))...)))))................. |
| ...........(((((.(((((.........((((((....))))))..(((((...........))))).)))))(....).((((.....))))...)))))................. |
| ...........(((((.(((((.........((((((....))))))..(((((...........))))).)))))(....).((((.....))))...)))))................. |
| ...........(((((.(((((.........((((((....))))))..(((((...........))))).)))))(....).((((.....))))...)))))................. |
| ...........(((((.(((((.........((((((....))))))..(((((...........))))).)))))(....).((((.....))))...)))))................. |
| ...........(((((.(((((.........((((((....))))))..(((((...........))))).)))))(....).((((.....))))...)))))................. |
| ...........(((((.(((((.........((((((....))))))..(((((...........))))).)))))(....).((((.....))))...)))))................. |
| ...........(((((.(((((.........((((((....))))))..(((((...........))))).)))))(....).((((.....))))...)))))................. |
| ...........(((((.(((((.........((((((....))))))..(((((...........))))).)))))(....).((((.....))))...)))))................. |
| ...........(((((.(((((.........((((((....))))))..(((((...........))))).)))))(....).((((.....))))...)))))................. |
| ...........(((((.(((((.........((((((....))))))..(((((...........))))).)))))(....).((((.....))))...)))))................. |
| ...........(((((.(((((.........((((((....))))))..(((((...........))))).)))))(....).((((.....))))...)))))................. |
| ...........(((((.(((((.........((((((....))))))..(((((...........))))).)))))(....).((((.....))))...)))))................. |
| ...........(((((.(((((.........((((((....))))))..(((((...........))))).)))))(....).((((.....))))...)))))................. |
| Chloramphenicol |
| ...........(((((.((((((.(((((......((((......)))).)))))......))))))(....).(((((...)))))...)))))................. |
| ...........(((((.((((((.(((((......((((......)))).)))))......))))))(....).(((((...)))))...)))))................. |
| ...........(((((.((((((.(((((......((((......)))).)))))......))))))(....).(((((...)))))...)))))................. |
| ...........(((((.((((((.(((((......((((......)))).)))))......))))))(....).(((((...)))))...)))))................. |
| ...........(((((.((((((.(((((......((((......)))).)))))......))))))(....).(((((...)))))...)))))................. |
| ...........(((((.((((((.(((((......((((......)))).)))))......))))))(....).(((((...)))))...)))))................. |
| ...........(((((.((((((.(((((......((((......)))).)))))......))))))(....).(((((...)))))...)))))................. |
| ...........(((((.((((((.(((((......((((......)))).)))))......))))))(....).(((((...)))))...)))))................. |
| ...........(((((.((((((.(((((......((((......)))).)))))......))))))(....).(((((...)))))...)))))................. |
| ...........(((((.((((((.(((((......((((......)))).)))))......))))))(....).(((((...)))))...)))))................. |
| ...........(((((.((((((.(((((......((((......)))).)))))......))))))(....).(((((...)))))...)))))................. |
| ...........(((((.((((((.(((((......((((......)))).)))))......))))))(....).(((((...)))))...)))))................. |
| ...........(((((.((((((.(((((......((((......)))).)))))......))))))(....).(((((...)))))...)))))................. |
| ...........(((((.((((((.(((((......((((......)))).)))))......))))))(....).(((((...)))))...)))))................. |
| ...........(((((.((((((.(((((......((((......)))).)))))......))))))(....).(((((...)))))...)))))................. |
| ...........(((((.((((((.(((((......((((......)))).)))))......))))))(....).(((((...)))))...)))))................. |
| ...........(((((.((((((.(((((......((((......)))).)))))......))))))(....).(((((...)))))...)))))................. |
| ...........(((((.((((((.(((((......((((......)))).)))))......))))))(....).(((((...)))))...)))))................. |
| ...........(((((.((((((.(((((......((((......)))).)))))......))))))(....).(((((...)))))...)))))................. |
| ...........(((((.((((((.(((((......((((......)))).)))))......))))))(....).(((((...)))))...)))))................. |
| ...........(((((.((((((.(((((......((((......)))).)))))......))))))(....).(((((...)))))...)))))................. |
| ...........(((((.((((((.(((((......((((......)))).)))))......))))))(....).(((((...)))))...)))))................. |
| ...........(((((.((((((.(((((......((((......)))).)))))......))))))(....).(((((...)))))...)))))................. |
| ...........(((((.((((((.(((((......((((......)))).)))))......))))))(....).(((((...)))))...)))))................. |
| ...........(((((.((((((.(((((......((((......)))).)))))......))))))(....).(((((...)))))...)))))................. |
| ...........(((((.((((((.(((((......((((......)))).)))))......))))))(....).(((((...)))))...)))))................. |
| ...........(((((.((((((.(((((......((((......)))).)))))......))))))(....).(((((...)))))...)))))................. |
| ...........(((((.((((((.(((((......((((......)))).)))))......))))))(....).(((((...)))))...)))))................. |
| ...........(((((.((((((.(((((......((((......)))).)))))......))))))(....).(((((...)))))...)))))................. |
| ...........(((((.((((((.(((((......((((......)))).)))))......))))))(....).(((((...)))))...)))))................. |
| ...........(((((.((((((.(((((......((((......)))).)))))......))))))(....).(((((...)))))...)))))................. |
| ...........(((((.((((((.(((((......((((......)))).)))))......))))))(....).(((((...)))))...)))))................. |
| ...........(((((.((((((.(((((......((((......)))).)))))......))))))(....).(((((...)))))...)))))................. |
| ...........(((((.((((((.(((((......((((......)))).)))))......))))))(....).(((((...)))))...)))))................. |
| ...........(((((.((((((.(((((......((((......)))).)))))......))))))(....).(((((...)))))...)))))................. |
| ...........(((((.((((((.(((((......((((......)))).)))))......))))))(....).(((((...)))))...)))))................. |
| ...........(((((.((((((.(((((......((((......)))).)))))......))))))(....).(((((...)))))...)))))................. |
| ...........(((((.((((((.(((((......((((......)))).)))))......))))))(....).(((((...)))))...)))))................. |
| ...........(((((.((((((.(((((......((((......)))).)))))......))))))(....).(((((...)))))...)))))................. |
| ...........(((((.((((((.(((((......((((......)))).)))))......))))))(....).(((((...)))))...)))))................. |
| ...........(((((.((((((.(((((......((((......)))).)))))......))))))(....).(((((...)))))...)))))................. |
| ...........(((((.((((((.(((((......((((......)))).)))))......))))))(....).(((((...)))))...)))))................. |
| ...........(((((.((((((.(((((......((((......)))).)))))......))))))(....).(((((...)))))...)))))................. |
| ...........(((((.((((((.(((((......((((......)))).)))))......))))))(....).(((((...)))))...)))))................. |
| ...........(((((.((((((.(((((......((((......)))).)))))......))))))(....).(((((...)))))...)))))................. |
| ...........(((((.((((((.(((((......((((......)))).)))))......))))))(....).(((((...)))))...)))))................. |
| ...........(((((.((((((.(((((......((((......)))).)))))......))))))(....).(((((...)))))...)))))................. |
| ...........(((((.((((((.(((((......((((......)))).)))))......))))))(....).(((((...)))))...)))))................. |
| ...........(((((.((((((.(((((......((((......)))).)))))......))))))(....).(((((...)))))...)))))................. |
| ...........(((((.((((((.(((((......((((......)))).)))))......))))))(....).(((((...)))))...)))))................. |
| ...........(((((.((((((.(((((......((((......)))).)))))......))))))(....).(((((...)))))...)))))................. |
| ...........(((((.((((((.(((((......((((......)))).)))))......))))))(....).(((((...)))))...)))))................. |
| ...........(((((.((((((.(((((......((((......)))).)))))......))))))(....).(((((...)))))...)))))................. |
| ...........(((((.((((((.(((((......((((......)))).)))))......))))))(....).(((((...)))))...)))))................. |
| ...........(((((.((((((.(((((......((((......)))).)))))......))))))(....).(((((...)))))...)))))................. |
| ...........(((((.((((((.(((((......((((......)))).)))))......))))))(....).(((((...)))))...)))))................. |
| ...........(((((.((((((.(((((......((((......)))).)))))......))))))(....).(((((...)))))...)))))................. |
| ...........(((((.((((((.(((((......((((......)))).)))))......))))))(....).(((((...)))))...)))))................. |
| ...........(((((.((((((.(((((......((((......)))).)))))......))))))(....).(((((...)))))...)))))................. |
| ...........(((((.((((((.(((((......((((......)))).)))))......))))))(....).(((((...)))))...)))))................. |
| ...........(((((.((((((.(((((......((((......)))).)))))......))))))(....).(((((...)))))...)))))................. |
| ...........(((((.((((((.(((((......((((......)))).)))))......))))))(....).(((((...)))))...)))))................. |
| ...........(((((.((((((.(((((......((((......)))).)))))......))))))(....).(((((...)))))...)))))................. |
| ...........(((((.((((((.(((((......((((......)))).)))))......))))))(....).(((((...)))))...)))))................. |
| ...........(((((.((((((.(((((......((((......)))).)))))......))))))(....).(((((...)))))...)))))................. |
| ...........(((((.((((((.(((((......((((......)))).)))))......))))))(....).(((((...)))))...)))))................. |
| ...........(((((.((((((.(((((......((((......)))).)))))......))))))(....).(((((...)))))...)))))................. |
| ...........(((((.((((((.(((((......((((......)))).)))))......))))))(....).(((((...)))))...)))))................. |
| ...........(((((.((((((.(((((......((((......)))).)))))......))))))(....).(((((...)))))...)))))................. |
| ...........(((((.((((((.(((((......((((......)))).)))))......))))))(....).(((((...)))))...)))))................. |
| ...........(((((.((((((.(((((......((((......)))).)))))......))))))(....).(((((...)))))...)))))................. |
| ...........(((((.((((((.(((((......((((......)))).)))))......))))))(....).(((((...)))))...)))))................. |
| ...........(((((.((((((.(((((......((((......)))).)))))......))))))(....).(((((...)))))...)))))................. |
| ...........(((((.((((((.(((((......((((......)))).)))))......))))))(....).(((((...)))))...)))))................. |
| ...........(((((.((((((.(((((......((((......)))).)))))......))))))(....).(((((...)))))...)))))................. |
| ...........(((((.((((((.(((((......((((......)))).)))))......))))))(....).(((((...)))))...)))))................. |
| ...........(((((.((((((.(((((......((((......)))).)))))......))))))(....).(((((...)))))...)))))................. |
| ...........(((((.((((((.(((((......((((......)))).)))))......))))))(....).(((((...)))))...)))))................. |
| ...........(((((.((((((.(((((......((((......)))).)))))......))))))(....).(((((...)))))...)))))................. |
| ...........(((((.((((((.(((((......((((......)))).)))))......))))))(....).(((((...)))))...)))))................. |
| ...........(((((.((((((.(((((......((((......)))).)))))......))))))(....).(((((...)))))...)))))................. |
| ...........(((((.((((((.(((((......((((......)))).)))))......))))))(....).(((((...)))))...)))))................. |
| ...........(((((.((((((.(((((......((((......)))).)))))......))))))(....).(((((...)))))...)))))................. |
| ...........(((((.((((((.(((((......((((......)))).)))))......))))))(....).(((((...)))))...)))))................. |
| ...........(((((.((((((.(((((......((((......)))).)))))......))))))(....).(((((...)))))...)))))................. |
| ...........(((((.((((((.(((((......((((......)))).)))))......))))))(....).(((((...)))))...)))))................. |
| ...........(((((.((((((.(((((......((((......)))).)))))......))))))(....).(((((...)))))...)))))................. |
| ...........(((((.((((((.(((((......((((......)))).)))))......))))))(....).(((((...)))))...)))))................. |
| ...........(((((.((((((.(((((......((((......)))).)))))......))))))(....).(((((...)))))...)))))................. |
| ...........(((((.((((((.(((((......((((......)))).)))))......))))))(....).(((((...)))))...)))))................. |
| ...........(((((.((((((.(((((......((((......)))).)))))......))))))(....).(((((...)))))...)))))................. |
| ...........(((((.((((((.(((((......((((......)))).)))))......))))))(....).(((((...)))))...)))))................. |
| ...........(((((.((((((.(((((......((((......)))).)))))......))))))(....).(((((...)))))...)))))................. |
| ...........(((((.((((((.(((((......((((......)))).)))))......))))))(....).(((((...)))))...)))))................. |
| ...........(((((.((((((.(((((......((((......)))).)))))......))))))(....).(((((...)))))...)))))................. |
| ...........(((((.((((((.(((((......((((......)))).)))))......))))))(....).(((((...)))))...)))))................. |
| ...........(((((.((((((.(((((......((((......)))).)))))......))))))(....).(((((...)))))...)))))................. |
| ...........(((((.((((((.(((((......((((......)))).)))))......))))))(....).(((((...)))))...)))))................. |
| ...........(((((.((((((.(((((......((((......)))).)))))......))))))(....).(((((...)))))...)))))................. |
| ...........(((((.((((((.(((((......((((......)))).)))))......))))))(....).(((((...)))))...)))))................. |
| Theophylline |
| ...........(((((.((((((...((((((((.....)))))...)))...))))))(....).(((((...)))))...)))))................. |
| ...........(((((.((((((...((((((((.....)))))...)))...))))))(....).(((((...)))))...)))))................. |
| ...........(((((.((((((...((((((((.....)))))...)))...))))))(....).(((((...)))))...)))))................. |
| ...........(((((.((((((...((((((((.....)))))...)))...))))))(....).(((((...)))))...)))))................. |
| ...........(((((.((((((...((((((((.....)))))...)))...))))))(....).(((((...)))))...)))))................. |
| ...........(((((.((((((...((((((((.....)))))...)))...))))))(....).(((((...)))))...)))))................. |
| ...........(((((.((((((...((((((((.....)))))...)))...))))))(....).(((((...)))))...)))))................. |
| ...........(((((.((((((...((((((((.....)))))...)))...))))))(....).(((((...)))))...)))))................. |
| ...........(((((.((((((...((((((((.....)))))...)))...))))))(....).(((((...)))))...)))))................. |
| ...........(((((.((((((...((((((((.....)))))...)))...))))))(....).(((((...)))))...)))))................. |
| ...........(((((.((((((...((((((((.....)))))...)))...))))))(....).(((((...)))))...)))))................. |
| ...........(((((.((((((...((((((((.....)))))...)))...))))))(....).(((((...)))))...)))))................. |
| ...........(((((.((((((...((((((((.....)))))...)))...))))))(....).(((((...)))))...)))))................. |
| ...........(((((.((((((...((((((((.....)))))...)))...))))))(....).(((((...)))))...)))))................. |
| ...........(((((.((((((...((((((((.....)))))...)))...))))))(....).(((((...)))))...)))))................. |
| ...........(((((.((((((...((((((((.....)))))...)))...))))))(....).(((((...)))))...)))))................. |
| ...........(((((.((((((...((((((((.....)))))...)))...))))))(....).(((((...)))))...)))))................. |
| ...........(((((.((((((...((((((((.....)))))...)))...))))))(....).(((((...)))))...)))))................. |
| ...........(((((.((((((...((((((((.....)))))...)))...))))))(....).(((((...)))))...)))))................. |
| ...........(((((.((((((...((((((((.....)))))...)))...))))))(....).(((((...)))))...)))))................. |
| ...........(((((.((((((...((((((((.....)))))...)))...))))))(....).(((((...)))))...)))))................. |
| ...........(((((.((((((...((((((((.....)))))...)))...))))))(....).(((((...)))))...)))))................. |
| ...........(((((.((((((...((((((((.....)))))...)))...))))))(....).(((((...)))))...)))))................. |
| ...........(((((.((((((...((((((((.....)))))...)))...))))))(....).(((((...)))))...)))))................. |
| ...........(((((.((((((...((((((((.....)))))...)))...))))))(....).(((((...)))))...)))))................. |
| ...........(((((.((((((...((((((((.....)))))...)))...))))))(....).(((((...)))))...)))))................. |
| ...........(((((.((((((...((((((((.....)))))...)))...))))))(....).(((((...)))))...)))))................. |
| ...........(((((.((((((...((((((((.....)))))...)))...))))))(....).(((((...)))))...)))))................. |
| ...........(((((.((((((...((((((((.....)))))...)))...))))))(....).(((((...)))))...)))))................. |
| ...........(((((.((((((...((((((((.....)))))...)))...))))))(....).(((((...)))))...)))))................. |
| ...........(((((.((((((...((((((((.....)))))...)))...))))))(....).(((((...)))))...)))))................. |
| ...........(((((.((((((...((((((((.....)))))...)))...))))))(....).(((((...)))))...)))))................. |
| ...........(((((.((((((...((((((((.....)))))...)))...))))))(....).(((((...)))))...)))))................. |
| ...........(((((.((((((...((((((((.....)))))...)))...))))))(....).(((((...)))))...)))))................. |
| ...........(((((.((((((...((((((((.....)))))...)))...))))))(....).(((((...)))))...)))))................. |
| ...........(((((.((((((...((((((((.....)))))...)))...))))))(....).(((((...)))))...)))))................. |
| ...........(((((.((((((...((((((((.....)))))...)))...))))))(....).(((((...)))))...)))))................. |
| ...........(((((.((((((...((((((((.....)))))...)))...))))))(....).(((((...)))))...)))))................. |
| ...........(((((.((((((...((((((((.....)))))...)))...))))))(....).(((((...)))))...)))))................. |
| ...........(((((.((((((...((((((((.....)))))...)))...))))))(....).(((((...)))))...)))))................. |
| ...........(((((.((((((...((((((((.....)))))...)))...))))))(....).(((((...)))))...)))))................. |
| ...........(((((.((((((...((((((((.....)))))...)))...))))))(....).(((((...)))))...)))))................. |
| ...........(((((.((((((...((((((((.....)))))...)))...))))))(....).(((((...)))))...)))))................. |
| ...........(((((.((((((...((((((((.....)))))...)))...))))))(....).(((((...)))))...)))))................. |
| ...........(((((.((((((...((((((((.....)))))...)))...))))))(....).(((((...)))))...)))))................. |
| ...........(((((.((((((...((((((((.....)))))...)))...))))))(....).(((((...)))))...)))))................. |
| ...........(((((.((((((...((((((((.....)))))...)))...))))))(....).(((((...)))))...)))))................. |
| ...........(((((.((((((...((((((((.....)))))...)))...))))))(....).(((((...)))))...)))))................. |
| ...........(((((.((((((...((((((((.....)))))...)))...))))))(....).(((((...)))))...)))))................. |
| ...........(((((.((((((...((((((((.....)))))...)))...))))))(....).(((((...)))))...)))))................. |
| ...........(((((.((((((...((((((((.....)))))...)))...))))))(....).(((((...)))))...)))))................. |
| ...........(((((.((((((...((((((((.....)))))...)))...))))))(....).(((((...)))))...)))))................. |
| ...........(((((.((((((...((((((((.....)))))...)))...))))))(....).(((((...)))))...)))))................. |
| ...........(((((.((((((...((((((((.....)))))...)))...))))))(....).(((((...)))))...)))))................. |
| ...........(((((.((((((...((((((((.....)))))...)))...))))))(....).(((((...)))))...)))))................. |
| ...........(((((.((((((...((((((((.....)))))...)))...))))))(....).(((((...)))))...)))))................. |
| ...........(((((.((((((...((((((((.....)))))...)))...))))))(....).(((((...)))))...)))))................. |
| ...........(((((.((((((...((((((((.....)))))...)))...))))))(....).(((((...)))))...)))))................. |
| ...........(((((.((((((...((((((((.....)))))...)))...))))))(....).(((((...)))))...)))))................. |
| ...........(((((.((((((...((((((((.....)))))...)))...))))))(....).(((((...)))))...)))))................. |
| ...........(((((.((((((...((((((((.....)))))...)))...))))))(....).(((((...)))))...)))))................. |
| ...........(((((.((((((...((((((((.....)))))...)))...))))))(....).(((((...)))))...)))))................. |
| ...........(((((.((((((...((((((((.....)))))...)))...))))))(....).(((((...)))))...)))))................. |
| ...........(((((.((((((...((((((((.....)))))...)))...))))))(....).(((((...)))))...)))))................. |
| ...........(((((.((((((...((((((((.....)))))...)))...))))))(....).(((((...)))))...)))))................. |
| ...........(((((.((((((...((((((((.....)))))...)))...))))))(....).(((((...)))))...)))))................. |
| ...........(((((.((((((...((((((((.....)))))...)))...))))))(....).(((((...)))))...)))))................. |
| ...........(((((.((((((...((((((((.....)))))...)))...))))))(....).(((((...)))))...)))))................. |
| ...........(((((.((((((...((((((((.....)))))...)))...))))))(....).(((((...)))))...)))))................. |
| ...........(((((.((((((...((((((((.....)))))...)))...))))))(....).(((((...)))))...)))))................. |
| ...........(((((.((((((...((((((((.....)))))...)))...))))))(....).(((((...)))))...)))))................. |
| ...........(((((.((((((...((((((((.....)))))...)))...))))))(....).(((((...)))))...)))))................. |
| ...........(((((.((((((...((((((((.....)))))...)))...))))))(....).(((((...)))))...)))))................. |
| ...........(((((.((((((...((((((((.....)))))...)))...))))))(....).(((((...)))))...)))))................. |
| ...........(((((.((((((...((((((((.....)))))...)))...))))))(....).(((((...)))))...)))))................. |
| ...........(((((.((((((...((((((((.....)))))...)))...))))))(....).(((((...)))))...)))))................. |
| ...........(((((.((((((...((((((((.....)))))...)))...))))))(....).(((((...)))))...)))))................. |
| ...........(((((.((((((...((((((((.....)))))...)))...))))))(....).(((((...)))))...)))))................. |
| ...........(((((.((((((...((((((((.....)))))...)))...))))))(....).(((((...)))))...)))))................. |
| ...........(((((.((((((...((((((((.....)))))...)))...))))))(....).(((((...)))))...)))))................. |
| ...........(((((.((((((...((((((((.....)))))...)))...))))))(....).(((((...)))))...)))))................. |
| ...........(((((.((((((...((((((((.....)))))...)))...))))))(....).(((((...)))))...)))))................. |
| ...........(((((.((((((...((((((((.....)))))...)))...))))))(....).(((((...)))))...)))))................. |
| ...........(((((.((((((...((((((((.....)))))...)))...))))))(....).(((((...)))))...)))))................. |
| ...........(((((.((((((...((((((((.....)))))...)))...))))))(....).(((((...)))))...)))))................. |
| ...........(((((.((((((...((((((((.....)))))...)))...))))))(....).(((((...)))))...)))))................. |
| ...........(((((.((((((...((((((((.....)))))...)))...))))))(....).(((((...)))))...)))))................. |
| ...........(((((.((((((...((((((((.....)))))...)))...))))))(....).(((((...)))))...)))))................. |
| ...........(((((.((((((...((((((((.....)))))...)))...))))))(....).(((((...)))))...)))))................. |
| ...........(((((.((((((...((((((((.....)))))...)))...))))))(....).(((((...)))))...)))))................. |
| ...........(((((.((((((...((((((((.....)))))...)))...))))))(....).(((((...)))))...)))))................. |
| ...........(((((.((((((...((((((((.....)))))...)))...))))))(....).(((((...)))))...)))))................. |
| ...........(((((.((((((...((((((((.....)))))...)))...))))))(....).(((((...)))))...)))))................. |
| ...........(((((.((((((...((((((((.....)))))...)))...))))))(....).(((((...)))))...)))))................. |
| ...........(((((.((((((...((((((((.....)))))...)))...))))))(....).(((((...)))))...)))))................. |
| ...........(((((.((((((...((((((((.....)))))...)))...))))))(....).(((((...)))))...)))))................. |
| ...........(((((.((((((...((((((((.....)))))...)))...))))))(....).(((((...)))))...)))))................. |
| ...........(((((.((((((...((((((((.....)))))...)))...))))))(....).(((((...)))))...)))))................. |
| ...........(((((.((((((...((((((((.....)))))...)))...))))))(....).(((((...)))))...)))))................. |
| ...........(((((.((((((...((((((((.....)))))...)))...))))))(....).(((((...)))))...)))))................. |
| Neomycin |
| ...........(((((.(((((((.....((......))..)))))))(....).((((.....))))...)))))................. |
| ...........(((((.(((((((.....((......))..)))))))(....).((((.....))))...)))))................. |
| ...........(((((.(((((((.....((......))..)))))))(....).((((.....))))...)))))................. |
| ...........(((((.(((((((.....((......))..)))))))(....).((((.....))))...)))))................. |
| ...........(((((.(((((((.....((......))..)))))))(....).((((.....))))...)))))................. |
| ...........(((((.(((((((.....((......))..)))))))(....).((((.....))))...)))))................. |
| ...........(((((.(((((((.....((......))..)))))))(....).((((.....))))...)))))................. |
| ...........(((((.(((((((.....((......))..)))))))(....).((((.....))))...)))))................. |
| ...........(((((.(((((((.....((......))..)))))))(....).((((.....))))...)))))................. |
| ...........(((((.(((((((.....((......))..)))))))(....).((((.....))))...)))))................. |
| ...........(((((.(((((((.....((......))..)))))))(....).((((.....))))...)))))................. |
| ...........(((((.(((((((.....((......))..)))))))(....).((((.....))))...)))))................. |
| ...........(((((.(((((((.....((......))..)))))))(....).((((.....))))...)))))................. |
| ...........(((((.(((((((.....((......))..)))))))(....).((((.....))))...)))))................. |
| ...........(((((.(((((((.....((......))..)))))))(....).((((.....))))...)))))................. |
| ...........(((((.(((((((.....((......))..)))))))(....).((((.....))))...)))))................. |
| ...........(((((.(((((((.....((......))..)))))))(....).((((.....))))...)))))................. |
| ...........(((((.(((((((.....((......))..)))))))(....).((((.....))))...)))))................. |
| ...........(((((.(((((((.....((......))..)))))))(....).((((.....))))...)))))................. |
| ...........(((((.(((((((.....((......))..)))))))(....).((((.....))))...)))))................. |
| ...........(((((.(((((((.....((......))..)))))))(....).((((.....))))...)))))................. |
| ...........(((((.(((((((.....((......))..)))))))(....).((((.....))))...)))))................. |
| ...........(((((.(((((((.....((......))..)))))))(....).((((.....))))...)))))................. |
| ...........(((((.(((((((.....((......))..)))))))(....).((((.....))))...)))))................. |
| ...........(((((.(((((((.....((......))..)))))))(....).((((.....))))...)))))................. |
| ...........(((((.(((((((.....((......))..)))))))(....).((((.....))))...)))))................. |
| ...........(((((.(((((((.....((......))..)))))))(....).((((.....))))...)))))................. |
| ...........(((((.(((((((.....((......))..)))))))(....).((((.....))))...)))))................. |
| ...........(((((.(((((((.....((......))..)))))))(....).((((.....))))...)))))................. |
| ...........(((((.(((((((.....((......))..)))))))(....).((((.....))))...)))))................. |
| ...........(((((.(((((((.....((......))..)))))))(....).((((.....))))...)))))................. |
| ...........(((((.(((((((.....((......))..)))))))(....).((((.....))))...)))))................. |
| ...........(((((.(((((((.....((......))..)))))))(....).((((.....))))...)))))................. |
| ...........(((((.(((((((.....((......))..)))))))(....).((((.....))))...)))))................. |
| ...........(((((.(((((((.....((......))..)))))))(....).((((.....))))...)))))................. |
| ...........(((((.(((((((.....((......))..)))))))(....).((((.....))))...)))))................. |
| ...........(((((.(((((((.....((......))..)))))))(....).((((.....))))...)))))................. |
| ...........(((((.(((((((.....((......))..)))))))(....).((((.....))))...)))))................. |
| ...........(((((.(((((((.....((......))..)))))))(....).((((.....))))...)))))................. |
| ...........(((((.(((((((.....((......))..)))))))(....).((((.....))))...)))))................. |
| ...........(((((.(((((((.....((......))..)))))))(....).((((.....))))...)))))................. |
| ...........(((((.(((((((.....((......))..)))))))(....).((((.....))))...)))))................. |
| ...........(((((.(((((((.....((......))..)))))))(....).((((.....))))...)))))................. |
| ...........(((((.(((((((.....((......))..)))))))(....).((((.....))))...)))))................. |
| ...........(((((.(((((((.....((......))..)))))))(....).((((.....))))...)))))................. |
| ...........(((((.(((((((.....((......))..)))))))(....).((((.....))))...)))))................. |
| ...........(((((.(((((((.....((......))..)))))))(....).((((.....))))...)))))................. |
| ...........(((((.(((((((.....((......))..)))))))(....).((((.....))))...)))))................. |
| ...........(((((.(((((((.....((......))..)))))))(....).((((.....))))...)))))................. |
| ...........(((((.(((((((.....((......))..)))))))(....).((((.....))))...)))))................. |
| ...........(((((.(((((((.....((......))..)))))))(....).((((.....))))...)))))................. |
| ...........(((((.(((((((.....((......))..)))))))(....).((((.....))))...)))))................. |
| ...........(((((.(((((((.....((......))..)))))))(....).((((.....))))...)))))................. |
| ...........(((((.(((((((.....((......))..)))))))(....).((((.....))))...)))))................. |
| ...........(((((.(((((((.....((......))..)))))))(....).((((.....))))...)))))................. |
| ...........(((((.(((((((.....((......))..)))))))(....).((((.....))))...)))))................. |
| ...........(((((.(((((((.....((......))..)))))))(....).((((.....))))...)))))................. |
| ...........(((((.(((((((.....((......))..)))))))(....).((((.....))))...)))))................. |
| ...........(((((.(((((((.....((......))..)))))))(....).((((.....))))...)))))................. |
| ...........(((((.(((((((.....((......))..)))))))(....).((((.....))))...)))))................. |
| ...........(((((.(((((((.....((......))..)))))))(....).((((.....))))...)))))................. |
| ...........(((((.(((((((.....((......))..)))))))(....).((((.....))))...)))))................. |
| ...........(((((.(((((((.....((......))..)))))))(....).((((.....))))...)))))................. |
| ...........(((((.(((((((.....((......))..)))))))(....).((((.....))))...)))))................. |
| ...........(((((.(((((((.....((......))..)))))))(....).((((.....))))...)))))................. |
| ...........(((((.(((((((.....((......))..)))))))(....).((((.....))))...)))))................. |
| ...........(((((.(((((((.....((......))..)))))))(....).((((.....))))...)))))................. |
| ...........(((((.(((((((.....((......))..)))))))(....).((((.....))))...)))))................. |
| ...........(((((.(((((((.....((......))..)))))))(....).((((.....))))...)))))................. |
| ...........(((((.(((((((.....((......))..)))))))(....).((((.....))))...)))))................. |
| ...........(((((.(((((((.....((......))..)))))))(....).((((.....))))...)))))................. |
| ...........(((((.(((((((.....((......))..)))))))(....).((((.....))))...)))))................. |
| ...........(((((.(((((((.....((......))..)))))))(....).((((.....))))...)))))................. |
| ...........(((((.(((((((.....((......))..)))))))(....).((((.....))))...)))))................. |
| ...........(((((.(((((((.....((......))..)))))))(....).((((.....))))...)))))................. |
| ...........(((((.(((((((.....((......))..)))))))(....).((((.....))))...)))))................. |
| ...........(((((.(((((((.....((......))..)))))))(....).((((.....))))...)))))................. |
| ...........(((((.(((((((.....((......))..)))))))(....).((((.....))))...)))))................. |
| ...........(((((.(((((((.....((......))..)))))))(....).((((.....))))...)))))................. |
| ...........(((((.(((((((.....((......))..)))))))(....).((((.....))))...)))))................. |
| ...........(((((.(((((((.....((......))..)))))))(....).((((.....))))...)))))................. |
| ...........(((((.(((((((.....((......))..)))))))(....).((((.....))))...)))))................. |
| ...........(((((.(((((((.....((......))..)))))))(....).((((.....))))...)))))................. |
| ...........(((((.(((((((.....((......))..)))))))(....).((((.....))))...)))))................. |
| ...........(((((.(((((((.....((......))..)))))))(....).((((.....))))...)))))................. |
| ...........(((((.(((((((.....((......))..)))))))(....).((((.....))))...)))))................. |
| ...........(((((.(((((((.....((......))..)))))))(....).((((.....))))...)))))................. |
| ...........(((((.(((((((.....((......))..)))))))(....).((((.....))))...)))))................. |
| ...........(((((.(((((((.....((......))..)))))))(....).((((.....))))...)))))................. |
| ...........(((((.(((((((.....((......))..)))))))(....).((((.....))))...)))))................. |
| ...........(((((.(((((((.....((......))..)))))))(....).((((.....))))...)))))................. |
| ...........(((((.(((((((.....((......))..)))))))(....).((((.....))))...)))))................. |
| ...........(((((.(((((((.....((......))..)))))))(....).((((.....))))...)))))................. |
| ...........(((((.(((((((.....((......))..)))))))(....).((((.....))))...)))))................. |
| ...........(((((.(((((((.....((......))..)))))))(....).((((.....))))...)))))................. |
| ...........(((((.(((((((.....((......))..)))))))(....).((((.....))))...)))))................. |
| ...........(((((.(((((((.....((......))..)))))))(....).((((.....))))...)))))................. |
| ...........(((((.(((((((.....((......))..)))))))(....).((((.....))))...)))))................. |
| ...........(((((.(((((((.....((......))..)))))))(....).((((.....))))...)))))................. |
| ...........(((((.(((((((.....((......))..)))))))(....).((((.....))))...)))))................. |
