# Supplementary material for: A convolutional neural network for the prediction and forward design of ribozyme-based gene-control elements
Source: eLife. 2021 Apr 16;10:e59697. doi: 10.7554/eLife.59697 (PMC8128436; doi:10.7554/eLife.59697)
Supplement: Supplementary file 6. — The sequences of loops I and II are indicated, one of which is the indicated aptamer, while the remainder of the ribozyme sequence is conserved. The final column contains the predicted basal gene-regulatory activity for a ribozyme with the two given loop sequences in terms of log10(GFP/mCherry). [file elife-59697-supp6.docx]

| **Aptamer** | **Loop I sequence** | **Loop II sequence** | **Predicted log10(GFP/mCh) value** |
| --- | --- | --- | --- |
| Chloramphenicol | ACAGTGAAAAAAGACGTGTGAATGTCACACTGAAAAAA | UAGAG | -1.067 |
| Chloramphenicol | ACAGTGAAAAAAGACGTGTGAATGTCACACTGAAAAAA | UAGGG | -1.054 |
| Chloramphenicol | ACAGTGAAAAAAGACGTGTGAATGTCACACTGAAAAAA | UGGGG | -1.051 |
| Chloramphenicol | ACAGTGAAAAAAGACGTGTGAATGTCACACTGAAAAAA | UAAGGG | -1.033 |
| Chloramphenicol | ACAGTGAAAAAAGACGTGTGAATGTCACACTGAAAAAA | UAGCG | -1.028 |
| Chloramphenicol | ACAGTGAAAAAAGACGTGTGAATGTCACACTGAAAAAA | UAAAGG | -1.022 |
| Chloramphenicol | ACAGTGAAAAAAGACGTGTGAATGTCACACTGAAAAAA | UAAGG | -1.021 |
| Chloramphenicol | ACAGTGAAAAAAGACGTGTGAATGTCACACTGAAAAAA | UAGGGG | -1.019 |
| Chloramphenicol | ACAGTGAAAAAAGACGTGTGAATGTCACACTGAAAAAA | UGAAG | -1.017 |
| Chloramphenicol | ACAGTGAAAAAAGACGTGTGAATGTCACACTGAAAAAA | UCGAG | -1.017 |
| Chloramphenicol | ACAGTGAAAAAAGACGTGTGAATGTCACACTGAAAAAA | UAGUAG | -1.015 |
| Chloramphenicol | ACAGTGAAAAAAGACGTGTGAATGTCACACTGAAAAAA | UAGAGG | -1.013 |
| Chloramphenicol | ACAGTGAAAAAAGACGTGTGAATGTCACACTGAAAAAA | UGGAA | -1.011 |
| Chloramphenicol | ACAGTGAAAAAAGACGTGTGAATGTCACACTGAAAAAA | UGGCG | -1.011 |
| Chloramphenicol | ACAGTGAAAAAAGACGTGTGAATGTCACACTGAAAAAA | UGGAG | -1.100 |
| Chloramphenicol | ACAGTGAAAAAAGACGTGTGAATGTCACACTGAAAAAA | UAGAAG | -1.010 |
| Chloramphenicol | ACAGTGAAAAAAGACGTGTGAATGTCACACTGAAAAAA | UAGGAG | -1.006 |
| Chloramphenicol | ACAGTGAAAAAAGACGTGTGAATGTCACACTGAAAAAA | UAGUGG | -1.005 |
| Chloramphenicol | ACAGTGAAAAAAGACGTGTGAATGTCACACTGAAAAAA | UGAGG | -0.998 |
| Chloramphenicol | ACAGTGAAAAAAGACGTGTGAATGTCACACTGAAAAAA | UAAAG | -0.989 |
| Chloramphenicol | ACAGTGAAAAAAGACGTGTGAATGTCACACTGAAAAAA | UAAAAG | -0.984 |
| Chloramphenicol | ACAGTGAAAAAAGACGTGTGAATGTCACACTGAAAAAA | UAAGAG | -0.983 |
| Chloramphenicol | ACAGTGAAAAAAGACGTGTGAATGTCACACTGAAAAAA | UGGUAG | -0.973 |
| Chloramphenicol | ACAGTGAAAAAAGACGTGTGAATGTCACACTGAAAAAA | UAGCAG | -0.965 |
| Chloramphenicol | ACAGTGAAAAAAGACGTGTGAATGTCACACTGAAAAAA | UAGCGG | -0.962 |
| Chloramphenicol | ACAGTGAAAAAAGACGTGTGAATGTCACACTGAAAAAA | UAUGGG | -0.959 |
| Chloramphenicol | ACAGTGAAAAAAGACGTGTGAATGTCACACTGAAAAAA | UGGAAG | -0.958 |
| Chloramphenicol | ACAGTGAAAAAAGACGTGTGAATGTCACACTGAAAAAA | UAGAA | -0.958 |
| Chloramphenicol | ACAGTGAAAAAAGACGTGTGAATGTCACACTGAAAAAA | UGGUG | -0.958 |
| Chloramphenicol | ACAGTGAAAAAAGACGTGTGAATGTCACACTGAAAAAA | UCGAA | -0.951 |
| Chloramphenicol | ACAGTGAAAAAAGACGTGTGAATGTCACACTGAAAAAA | UAAUGG | -0.949 |
| Chloramphenicol | ACAGTGAAAAAAGACGTGTGAATGTCACACTGAAAAAA | UGGGAG | -0.948 |
| Chloramphenicol | ACAGTGAAAAAAGACGTGTGAATGTCACACTGAAAAAA | UGAGGG | -0.947 |
| Chloramphenicol | ACAGTGAAAAAAGACGTGTGAATGTCACACTGAAAAAA | UAACGG | -0.946 |
| Chloramphenicol | ACAGTGAAAAAAGACGTGTGAATGTCACACTGAAAAAA | UGGGA | -0.946 |
| Chloramphenicol | ACAGTGAAAAAAGACGTGTGAATGTCACACTGAAAAAA | UAGUG | -0.942 |
| Chloramphenicol | ACAGTGAAAAAAGACGTGTGAATGTCACACTGAAAAAA | UAUAGG | -0.941 |
| Chloramphenicol | ACAGTGAAAAAAGACGTGTGAATGTCACACTGAAAAAA | UGGUGG | -0.941 |
| Chloramphenicol | ACAGTGAAAAAAGACGTGTGAATGTCACACTGAAAAAA | UAGGCG | -0.941 |
| Chloramphenicol | ACAGTGAAAAAAGACGTGTGAATGTCACACTGAAAAAA | UGGGGG | -0.941 |
| Chloramphenicol | ACAGTGAAAAAAGACGTGTGAATGTCACACTGAAAAAA | UCGGG | -0.941 |
| Chloramphenicol | ACAGTGAAAAAAGACGTGTGAATGTCACACTGAAAAAA | UGGCA | -0.940 |
| Chloramphenicol | ACAGTGAAAAAAGACGTGTGAATGTCACACTGAAAAAA | UGAAGG | -0.937 |
| Chloramphenicol | ACAGTGAAAAAAGACGTGTGAATGTCACACTGAAAAAA | UGGAGG | -0.936 |
| Chloramphenicol | ACAGTGAAAAAAGACGTGTGAATGTCACACTGAAAAAA | UGAAAG | -0.935 |
| Chloramphenicol | ACAGTGAAAAAAGACGTGTGAATGTCACACTGAAAAAA | UAUGG | -0.932 |
| Chloramphenicol | ACAGTGAAAAAAGACGTGTGAATGTCACACTGAAAAAA | UGAGAG | -0.928 |
| Chloramphenicol | ACAGTGAAAAAAGACGTGTGAATGTCACACTGAAAAAA | UGAAA | -0.928 |
| Chloramphenicol | ACAGTGAAAAAAGACGTGTGAATGTCACACTGAAAAAA | UGUAG | -0.924 |
| Chloramphenicol | ACAGTGAAAAAAGACGTGTGAATGTCACACTGAAAAAA | UAUGAG | -0.923 |
| Chloramphenicol | ACAGTGAAAAAAGACGTGTGAATGTCACACTGAAAAAA | UAUAAG | -0.921 |
| Chloramphenicol | ACAGTGAAAAAAGACGTGTGAATGTCACACTGAAAAAA | UAAUAG | -0.921 |
| Chloramphenicol | ACAGTGAAAAAAGACGTGTGAATGTCACACTGAAAAAA | UAGGA | -0.914 |
| Chloramphenicol | ACAGTGAAAAAAGACGTGTGAATGTCACACTGAAAAAA | UAUAG | -0.910 |
| Chloramphenicol | ACAGTGAAAAAAGACGTGTGAATGTCACACTGAAAAAA | UAGUCG | -0.908 |
| Chloramphenicol | ACAGTGAAAAAAGACGTGTGAATGTCACACTGAAAAAA | UAACAG | -0.908 |
| Chloramphenicol | ACAGTGAAAAAAGACGTGTGAATGTCACACTGAAAAAA | UAACG | -0.907 |
| Chloramphenicol | ACAGTGAAAAAAGACGTGTGAATGTCACACTGAAAAAA | UAGGUG | -0.906 |
| Chloramphenicol | ACAGTGAAAAAAGACGTGTGAATGTCACACTGAAAAAA | UGUGG | -0.905 |
| Chloramphenicol | ACAGTGAAAAAAGACGTGTGAATGTCACACTGAAAAAA | CAGGG | -0.902 |
| Chloramphenicol | ACAGTGAAAAAAGACGTGTGAATGTCACACTGAAAAAA | UGGCAG | -0.900 |
| Chloramphenicol | ACAGTGAAAAAAGACGTGTGAATGTCACACTGAAAAAA | UAGACG | -0.900 |
| Chloramphenicol | ACAGTGAAAAAAGACGTGTGAATGTCACACTGAAAAAA | UCGGAG | -0.900 |
| Chloramphenicol | ACAGTGAAAAAAGACGTGTGAATGTCACACTGAAAAAA | UCAAG | -0.899 |
| Chloramphenicol | ACAGTGAAAAAAGACGTGTGAATGTCACACTGAAAAAA | UAGCA | -0.899 |
| Chloramphenicol | ACAGTGAAAAAAGACGTGTGAATGTCACACTGAAAAAA | CGGAG | -0.897 |
| Chloramphenicol | ACAGTGAAAAAAGACGTGTGAATGTCACACTGAAAAAA | UGAUGG | -0.892 |
| Chloramphenicol | ACAGTGAAAAAAGACGTGTGAATGTCACACTGAAAAAA | UGUGGG | -0.892 |
| Chloramphenicol | ACAGTGAAAAAAGACGTGTGAATGTCACACTGAAAAAA | UGACG | -0.890 |
| Chloramphenicol | ACAGTGAAAAAAGACGTGTGAATGTCACACTGAAAAAA | CGGGG | -0.890 |
| Chloramphenicol | ACAGTGAAAAAAGACGTGTGAATGTCACACTGAAAAAA | UGUAAG | -0.889 |
| Chloramphenicol | ACAGTGAAAAAAGACGTGTGAATGTCACACTGAAAAAA | UGAGA | -0.887 |
| Chloramphenicol | ACAGTGAAAAAAGACGTGTGAATGTCACACTGAAAAAA | UAGGGA | -0.885 |
| Chloramphenicol | ACAGTGAAAAAAGACGTGTGAATGTCACACTGAAAAAA | UGUGAG | -0.884 |
| Chloramphenicol | ACAGTGAAAAAAGACGTGTGAATGTCACACTGAAAAAA | UGAUAG | -0.884 |
| Chloramphenicol | ACAGTGAAAAAAGACGTGTGAATGTCACACTGAAAAAA | UCGGGG | -0.884 |
| Chloramphenicol | ACAGTGAAAAAAGACGTGTGAATGTCACACTGAAAAAA | UCGAAG | -0.883 |
| Chloramphenicol | ACAGTGAAAAAAGACGTGTGAATGTCACACTGAAAAAA | UAGCCG | -0.883 |
| Chloramphenicol | ACAGTGAAAAAAGACGTGTGAATGTCACACTGAAAAAA | UAGAUG | -0.883 |
| Chloramphenicol | ACAGTGAAAAAAGACGTGTGAATGTCACACTGAAAAAA | UAAAA | -0.882 |
| Chloramphenicol | ACAGTGAAAAAAGACGTGTGAATGTCACACTGAAAAAA | UUGAG | -0.881 |
| Chloramphenicol | ACAGTGAAAAAAGACGTGTGAATGTCACACTGAAAAAA | UAAGGA | -0.880 |
| Chloramphenicol | ACAGTGAAAAAAGACGTGTGAATGTCACACTGAAAAAA | UGGCGG | -0.879 |
| Chloramphenicol | ACAGTGAAAAAAGACGTGTGAATGTCACACTGAAAAAA | UAAAGA | -0.878 |
| Chloramphenicol | ACAGTGAAAAAAGACGTGTGAATGTCACACTGAAAAAA | UAUCGG | -0.878 |
| Chloramphenicol | ACAGTGAAAAAAGACGTGTGAATGTCACACTGAAAAAA | UAGAGA | -0.878 |
| Chloramphenicol | ACAGTGAAAAAAGACGTGTGAATGTCACACTGAAAAAA | UAAGA | -0.878 |
| Chloramphenicol | ACAGTGAAAAAAGACGTGTGAATGTCACACTGAAAAAA | UUGGAG | -0.877 |
| Chloramphenicol | ACAGTGAAAAAAGACGTGTGAATGTCACACTGAAAAAA | UGUAGG | -0.877 |
| Chloramphenicol | ACAGTGAAAAAAGACGTGTGAATGTCACACTGAAAAAA | UGGUA | -0.875 |
| Chloramphenicol | ACAGTGAAAAAAGACGTGTGAATGTCACACTGAAAAAA | CAAGG | -0.873 |
| Chloramphenicol | ACAGTGAAAAAAGACGTGTGAATGTCACACTGAAAAAA | UAUUAG | -0.869 |
| Chloramphenicol | ACAGTGAAAAAAGACGTGTGAATGTCACACTGAAAAAA | UAUUGG | -0.869 |
| Chloramphenicol | ACAGTGAAAAAAGACGTGTGAATGTCACACTGAAAAAA | UAUCAG | -0.868 |
| Chloramphenicol | ACAGTGAAAAAAGACGTGTGAATGTCACACTGAAAAAA | UCAGAG | -0.867 |
| Chloramphenicol | ACAGTGAAAAAAGACGTGTGAATGTCACACTGAAAAAA | UAGUGA | -0.866 |
| Chloramphenicol | ACAGTGAAAAAAGACGTGTGAATGTCACACTGAAAAAA | UGGUAA | -0.866 |
| Chloramphenicol | ACAGTGAAAAAAGACGTGTGAATGTCACACTGAAAAAA | UGGGUG | -0.865 |
| Chloramphenicol | ACAGTGAAAAAAGACGTGTGAATGTCACACTGAAAAAA | UCGUAG | -0.865 |
| Chloramphenicol | ACAGTGAAAAAAGACGTGTGAATGTCACACTGAAAAAA | UGGGCG | -0.865 |
| Chloramphenicol | ACAGTGAAAAAAGACGTGTGAATGTCACACTGAAAAAA | UAAGCG | -0.862 |
| Chloramphenicol | ACAGTGAAAAAAGACGTGTGAATGTCACACTGAAAAAA | UAGUUG | -0.860 |
| Chloramphenicol | ACAGTGAAAAAAGACGTGTGAATGTCACACTGAAAAAA | UAGCUG | -0.859 |
| Chloramphenicol | ACAGTGAAAAAAGACGTGTGAATGTCACACTGAAAAAA | UUGGG | -0.858 |
| Chloramphenicol | ACAGTGAAAAAAGACGTGTGAATGTCACACTGAAAAAA | UCAGG | -0.858 |
| Chloramphenicol | ACAGTGAAAAAAGACGTGTGAATGTCACACTGAAAAAA | UCAGGG | -0.858 |
| Chloramphenicol | ACAGTGAAAAAAGACGTGTGAATGTCACACTGAAAAAA | UAAGUG | -0.857 |
| Chloramphenicol | ACAGTGAAAAAAGACGTGTGAATGTCACACTGAAAAAA | UUAGGG | -0.856 |
| Chloramphenicol | ACAGTGAAAAAAGACGTGTGAATGTCACACTGAAAAAA | UUGUAG | -0.855 |
| Chloramphenicol | ACAGTGAAAAAAGACGTGTGAATGTCACACTGAAAAAA | UGGAAA | -0.854 |
| Chloramphenicol | ACAGTGAAAAAAGACGTGTGAATGTCACACTGAAAAAA | UUGGGG | -0.853 |
| Chloramphenicol | ACAGTGAAAAAAGACGTGTGAATGTCACACTGAAAAAA | UGUUAG | -0.852 |
| Chloramphenicol | ACAGTGAAAAAAGACGTGTGAATGTCACACTGAAAAAA | UUAGAG | -0.852 |
| Chloramphenicol | ACAGTGAAAAAAGACGTGTGAATGTCACACTGAAAAAA | UAUCG | -0.851 |
| Chloramphenicol | ACAGTGAAAAAAGACGTGTGAATGTCACACTGAAAAAA | UGGUCG | -0.851 |
| Chloramphenicol | ACAGTGAAAAAAGACGTGTGAATGTCACACTGAAAAAA | UGGAUG | -0.851 |
| Chloramphenicol | ACAGTGAAAAAAGACGTGTGAATGTCACACTGAAAAAA | UGGGAA | -0.850 |
| Chloramphenicol | ACAGTGAAAAAAGACGTGTGAATGTCACACTGAAAAAA | UAGCGA | -0.850 |
| Chloramphenicol | ACAGTGAAAAAAGACGTGTGAATGTCACACTGAAAAAA | UUGAAG | -0.850 |
| Chloramphenicol | ACAGTGAAAAAAGACGTGTGAATGTCACACTGAAAAAA | UUGCAG | -0.846 |
| Chloramphenicol | ACAGTGAAAAAAGACGTGTGAATGTCACACTGAAAAAA | UGGUGA | -0.844 |
| Chloramphenicol | ACAGTGAAAAAAGACGTGTGAATGTCACACTGAAAAAA | UGUAA | -0.844 |
| Chloramphenicol | ACAGTGAAAAAAGACGTGTGAATGTCACACTGAAAAAA | UGGGGA | -0.841 |
| Chloramphenicol | ACAGTGAAAAAAGACGTGTGAATGTCACACTGAAAAAA | UGGAGA | -0.841 |
| Chloramphenicol | ACAGTGAAAAAAGACGTGTGAATGTCACACTGAAAAAA | CGAGG | -0.840 |
| Chloramphenicol | ACAGTGAAAAAAGACGTGTGAATGTCACACTGAAAAAA | UCGCAG | -0.840 |
| Chloramphenicol | ACAGTGAAAAAAGACGTGTGAATGTCACACTGAAAAAA | UCAAA | -0.840 |
| Chloramphenicol | ACAGTGAAAAAAGACGTGTGAATGTCACACTGAAAAAA | UGCAG | -0.839 |
| Chloramphenicol | ACAGTGAAAAAAGACGTGTGAATGTCACACTGAAAAAA | UAGGAA | -0.837 |
| Chloramphenicol | ACAGTGAAAAAAGACGTGTGAATGTCACACTGAAAAAA | UAGAAA | -0.836 |
| Chloramphenicol | ACAGTGAAAAAAGACGTGTGAATGTCACACTGAAAAAA | UAGUAA | -0.835 |
| Chloramphenicol | ACAGTGAAAAAAGACGTGTGAATGTCACACTGAAAAAA | UCGAGG | -0.835 |
| Chloramphenicol | ACAGTGAAAAAAGACGTGTGAATGTCACACTGAAAAAA | UGACAG | -0.835 |
| Chloramphenicol | ACAGTGAAAAAAGACGTGTGAATGTCACACTGAAAAAA | UGACGG | -0.834 |
| Chloramphenicol | ACAGTGAAAAAAGACGTGTGAATGTCACACTGAAAAAA | UGGUUG | -0.834 |
| Chloramphenicol | ACAGTGAAAAAAGACGTGTGAATGTCACACTGAAAAAA | UGAAGA | -0.834 |
| Chloramphenicol | ACAGTGAAAAAAGACGTGTGAATGTCACACTGAAAAAA | UGAUG | -0.831 |
| Chloramphenicol | ACAGTGAAAAAAGACGTGTGAATGTCACACTGAAAAAA | UGAGGA | -0.829 |
| Chloramphenicol | ACAGTGAAAAAAGACGTGTGAATGTCACACTGAAAAAA | UGGACG | -0.829 |
| Chloramphenicol | ACAGTGAAAAAAGACGTGTGAATGTCACACTGAAAAAA | UAUGCG | -0.826 |
| Chloramphenicol | ACAGTGAAAAAAGACGTGTGAATGTCACACTGAAAAAA | UGUUGG | -0.826 |
| Chloramphenicol | ACAGTGAAAAAAGACGTGTGAATGTCACACTGAAAAAA | UCGGA | -0.824 |
| Chloramphenicol | ACAGTGAAAAAAGACGTGTGAATGTCACACTGAAAAAA | UGUCG | -0.824 |
| Chloramphenicol | ACAGTGAAAAAAGACGTGTGAATGTCACACTGAAAAAA | UGAAAA | -0.823 |
| Chloramphenicol | ACAGTGAAAAAAGACGTGTGAATGTCACACTGAAAAAA | UGGCAA | -0.822 |
| Chloramphenicol | ACAGTGAAAAAAGACGTGTGAATGTCACACTGAAAAAA | UAAUG | -0.822 |
| Chloramphenicol | ACAGTGAAAAAAGACGTGTGAATGTCACACTGAAAAAA | UCAAAG | -0.821 |
| Chloramphenicol | ACAGTGAAAAAAGACGTGTGAATGTCACACTGAAAAAA | CAGCG | -0.820 |
| Chloramphenicol | ACAGTGAAAAAAGACGTGTGAATGTCACACTGAAAAAA | UGACA | -0.820 |
| Chloramphenicol | ACAGTGAAAAAAGACGTGTGAATGTCACACTGAAAAAA | CGGCG | -0.820 |
| Chloramphenicol | ACAGTGAAAAAAGACGTGTGAATGTCACACTGAAAAAA | UUGAGG | -0.820 |
| Chloramphenicol | ACAGTGAAAAAAGACGTGTGAATGTCACACTGAAAAAA | UCGGAA | -0.820 |
| Chloramphenicol | ACAGTGAAAAAAGACGTGTGAATGTCACACTGAAAAAA | UGUCAG | -0.818 |
| Chloramphenicol | ACAGTGAAAAAAGACGTGTGAATGTCACACTGAAAAAA | UCUGAG | -0.818 |
| Chloramphenicol | ACAGTGAAAAAAGACGTGTGAATGTCACACTGAAAAAA | UUAAAG | -0.818 |
| Chloramphenicol | ACAGTGAAAAAAGACGTGTGAATGTCACACTGAAAAAA | CGAAG | -0.817 |
| Chloramphenicol | ACAGTGAAAAAAGACGTGTGAATGTCACACTGAAAAAA | UACGGG | -0.816 |
| Chloramphenicol | ACAGTGAAAAAAGACGTGTGAATGTCACACTGAAAAAA | UGAGAA | -0.815 |
| Chloramphenicol | ACAGTGAAAAAAGACGTGTGAATGTCACACTGAAAAAA | UGAGUG | -0.815 |
| Chloramphenicol | ACAGTGAAAAAAGACGTGTGAATGTCACACTGAAAAAA | CAGAG | -0.815 |
| Chloramphenicol | ACAGTGAAAAAAGACGTGTGAATGTCACACTGAAAAAA | UAACGA | -0.814 |
| Chloramphenicol | ACAGTGAAAAAAGACGTGTGAATGTCACACTGAAAAAA | UCUAG | -0.814 |
| Chloramphenicol | ACAGTGAAAAAAGACGTGTGAATGTCACACTGAAAAAA | UAGCAA | -0.813 |
| Chloramphenicol | ACAGTGAAAAAAGACGTGTGAATGTCACACTGAAAAAA | UAUGGA | -0.813 |
| Chloramphenicol | ACAGTGAAAAAAGACGTGTGAATGTCACACTGAAAAAA | UAAAUG | -0.813 |
| Chloramphenicol | ACAGTGAAAAAAGACGTGTGAATGTCACACTGAAAAAA | UGUCGG | -0.810 |
| Chloramphenicol | ACAGTGAAAAAAGACGTGTGAATGTCACACTGAAAAAA | UUGAA | -0.810 |
| Chloramphenicol | ACAGTGAAAAAAGACGTGTGAATGTCACACTGAAAAAA | UGGCGA | -0.807 |
| Chloramphenicol | ACAGTGAAAAAAGACGTGTGAATGTCACACTGAAAAAA | UAAAAA | -0.807 |
| Chloramphenicol | ACAGTGAAAAAAGACGTGTGAATGTCACACTGAAAAAA | UAUAA | -0.806 |
| Chloramphenicol | ACAGTGAAAAAAGACGTGTGAATGTCACACTGAAAAAA | UACAGG | -0.806 |
| Chloramphenicol | ACAGTGAAAAAAGACGTGTGAATGTCACACTGAAAAAA | UCUGGG | -0.805 |
| Chloramphenicol | ACAGTGAAAAAAGACGTGTGAATGTCACACTGAAAAAA | UCGUGG | -0.805 |
| Chloramphenicol | ACAGTGAAAAAAGACGTGTGAATGTCACACTGAAAAAA | UUAGG | -0.805 |
| Chloramphenicol | ACAGTGAAAAAAGACGTGTGAATGTCACACTGAAAAAA | UAAUGA | -0.805 |
| Chloramphenicol | ACAGTGAAAAAAGACGTGTGAATGTCACACTGAAAAAA | UGUGA | -0.804 |
| Chloramphenicol | ACAGTGAAAAAAGACGTGTGAATGTCACACTGAAAAAA | UAGUA | -0.804 |
| Chloramphenicol | ACAGTGAAAAAAGACGTGTGAATGTCACACTGAAAAAA | UUUGAG | -0.804 |
| Chloramphenicol | ACAGTGAAAAAAGACGTGTGAATGTCACACTGAAAAAA | UCGAAA | -0.803 |
| Chloramphenicol | ACAGTGAAAAAAGACGTGTGAATGTCACACTGAAAAAA | UGCGG | -0.802 |
| Chloramphenicol | ACAGTGAAAAAAGACGTGTGAATGTCACACTGAAAAAA | UAAGAA | -0.801 |
| Chloramphenicol | ACAGTGAAAAAAGACGTGTGAATGTCACACTGAAAAAA | UAUAGA | -0.801 |
| Chloramphenicol | ACAGTGAAAAAAGACGTGTGAATGTCACACTGAAAAAA | UAAACG | -0.801 |
| Chloramphenicol | ACAGTGAAAAAAGACGTGTGAATGTCACACTGAAAAAA | UCGCG | -0.801 |
| Chloramphenicol | ACAGTGAAAAAAGACGTGTGAATGTCACACTGAAAAAA | UCAAGG | -0.801 |
| Chloramphenicol | ACAGTGAAAAAAGACGTGTGAATGTCACACTGAAAAAA | UCGUG | -0.801 |
| Chloramphenicol | ACAGTGAAAAAAGACGTGTGAATGTCACACTGAAAAAA | UAUGUG | -0.800 |
| Chloramphenicol | ACAGTGAAAAAAGACGTGTGAATGTCACACTGAAAAAA | UUAAGG | -0.799 |
| Chloramphenicol | ACAGTGAAAAAAGACGTGTGAATGTCACACTGAAAAAA | UACCGG | -0.798 |
| Chloramphenicol | ACAGTGAAAAAAGACGTGTGAATGTCACACTGAAAAAA | UCUAAG | -0.797 |
| Chloramphenicol | ACAGTGAAAAAAGACGTGTGAATGTCACACTGAAAAAA | UGGCUG | -0.796 |
| Chloramphenicol | ACAGTGAAAAAAGACGTGTGAATGTCACACTGAAAAAA | UUGCGG | -0.796 |
| Chloramphenicol | ACAGTGAAAAAAGACGTGTGAATGTCACACTGAAAAAA | UAUGA | -0.794 |
| Chloramphenicol | ACAGTGAAAAAAGACGTGTGAATGTCACACTGAAAAAA | UACGG | -0.794 |
| Chloramphenicol | ACAGTGAAAAAAGACGTGTGAATGTCACACTGAAAAAA | UUGUGG | -0.794 |
| Chloramphenicol | ACAGTGAAAAAAGACGTGTGAATGTCACACTGAAAAAA | CAAGGG | -0.794 |
| Chloramphenicol | ACAGTGAAAAAAGACGTGTGAATGTCACACTGAAAAAA | UACGAG | -0.791 |
| Chloramphenicol | ACAGTGAAAAAAGACGTGTGAATGTCACACTGAAAAAA | UUUGGG | -0.790 |
| Chloramphenicol | ACAGTGAAAAAAGACGTGTGAATGTCACACTGAAAAAA | UGAUGA | -0.790 |
| Chloramphenicol | ACAGTGAAAAAAGACGTGTGAATGTCACACTGAAAAAA | UGGCCG | -0.788 |
| Folinic Acid | GCUUGGUACGUUAUAUUCA | UGGAG | -0.712 |
| Folinic Acid | GCUUGGUACGUUAUAUUCA | UAGGG | -0.702 |
| Folinic Acid | GCUUGGUACGUUAUAUUCA | UGGGG | -0.699 |
| Folinic Acid | UAGGAG | GCUUGGUACGUUAUAUUCA | -0.685 |
| Folinic Acid | UAGGGG | GCUUGGUACGUUAUAUUCA | -0.673 |
| Folinic Acid | GCUUGGUACGUUAUAUUCA | UAAGG | -0.672 |
| Folinic Acid | UAAGAG | GCUUGGUACGUUAUAUUCA | -0.667 |
| Folinic Acid | GCUUGGUACGUUAUAUUCA | UGAGG | -0.661 |
| Folinic Acid | GCUUGGUACGUUAUAUUCA | UAGAG | -0.656 |
| Folinic Acid | UAGUAG | GCUUGGUACGUUAUAUUCA | -0.654 |
| Folinic Acid | UAAGGG | GCUUGGUACGUUAUAUUCA | -0.653 |
| Folinic Acid | GCUUGGUACGUUAUAUUCA | UGAAG | -0.652 |
| Folinic Acid | GCUUGGUACGUUAUAUUCA | UGGAA | -0.648 |
| Folinic Acid | UAGAAG | GCUUGGUACGUUAUAUUCA | -0.643 |
| Folinic Acid | UAUGAG | GCUUGGUACGUUAUAUUCA | -0.641 |
| Folinic Acid | UAGUGG | GCUUGGUACGUUAUAUUCA | -0.633 |
| Folinic Acid | UAAUAG | GCUUGGUACGUUAUAUUCA | -0.628 |
| Folinic Acid | UAUGGG | GCUUGGUACGUUAUAUUCA | -0.627 |
| Folinic Acid | UAGAGG | GCUUGGUACGUUAUAUUCA | -0.626 |
| Folinic Acid | UGGGAG | GCUUGGUACGUUAUAUUCA | -0.624 |
| Folinic Acid | GCUUGGUACGUUAUAUUCA | UGGGA | -0.624 |
| Folinic Acid | UAGGUG | GCUUGGUACGUUAUAUUCA | -0.622 |
| Folinic Acid | UAAAAG | GCUUGGUACGUUAUAUUCA | -0.620 |
| Folinic Acid | GCUUGGUACGUUAUAUUCA | UGUAG | -0.616 |
| Folinic Acid | GCUUGGUACGUUAUAUUCA | UAGGA | -0.614 |
| Folinic Acid | UAAGUG | GCUUGGUACGUUAUAUUCA | -0.610 |
| Folinic Acid | GCUUGGUACGUUAUAUUCA | UGGUG | -0.609 |
| Folinic Acid | GCUUGGUACGUUAUAUUCA | UGUGG | -0.607 |
| Folinic Acid | GCUUGGUACGUUAUAUUCA | UAUGG | -0.607 |
| Folinic Acid | UAGGGGG | GCUUGGUACGUUAUAUUCA | -0.606 |
| Folinic Acid | UAGGGGGG | GCUUGGUACGUUAUAUUCA | -0.605 |
| Folinic Acid | GCUUGGUACGUUAUAUUCA | UAAAG | -0.603 |
| Folinic Acid | UAUAAG | GCUUGGUACGUUAUAUUCA | -0.602 |
| Folinic Acid | UGGGGG | GCUUGGUACGUUAUAUUCA | -0.602 |
| Folinic Acid | UAAUGG | GCUUGGUACGUUAUAUUCA | -0.601 |
| Folinic Acid | UAAAGG | GCUUGGUACGUUAUAUUCA | -0.600 |
| Folinic Acid | UAGGAGGG | GCUUGGUACGUUAUAUUCA | -0.599 |
| Folinic Acid | UUGGAG | GCUUGGUACGUUAUAUUCA | -0.599 |
| Folinic Acid | UAUUAG | GCUUGGUACGUUAUAUUCA | -0.598 |
| Folinic Acid | UAAGGGGG | GCUUGGUACGUUAUAUUCA | -0.598 |
| Folinic Acid | UAAGAGGG | GCUUGGUACGUUAUAUUCA | -0.597 |
| Folinic Acid | UGAGAG | GCUUGGUACGUUAUAUUCA | -0.596 |
| Folinic Acid | UGGUAG | GCUUGGUACGUUAUAUUCA | -0.596 |
| Folinic Acid | UAAGGGG | GCUUGGUACGUUAUAUUCA | -0.596 |
| Folinic Acid | UAGGUGGG | GCUUGGUACGUUAUAUUCA | -0.594 |
| Folinic Acid | UAUGGGG | GCUUGGUACGUUAUAUUCA | -0.591 |
| Folinic Acid | UGAAG | GCUUGGUACGUUAUAUUCA | -0.591 |
| Folinic Acid | UAAAG | GCUUGGUACGUUAUAUUCA | -0.589 |
| Folinic Acid | UAAGUGGG | GCUUGGUACGUUAUAUUCA | -0.589 |
| Folinic Acid | UGGAAG | GCUUGGUACGUUAUAUUCA | -0.589 |
| Folinic Acid | UUGGGG | GCUUGGUACGUUAUAUUCA | -0.589 |
| Folinic Acid | UAUGAGGG | GCUUGGUACGUUAUAUUCA | -0.588 |
| Folinic Acid | UAGUUG | GCUUGGUACGUUAUAUUCA | -0.588 |
| Folinic Acid | UGGAG | GCUUGGUACGUUAUAUUCA | -0.587 |
| Folinic Acid | UGGGGG | GCUUGGUACGUUAUAUUCA | -0.587 |
| Folinic Acid | GCUUGGUACGUUAUAUUCA | UGAGA | -0.587 |
| Folinic Acid | UAGGGG | GCUUGGUACGUUAUAUUCA | -0.586 |
| Folinic Acid | UAGGGAGG | GCUUGGUACGUUAUAUUCA | -0.586 |
| Folinic Acid | GCUUGGUACGUUAUAUUCA | UGAAA | -0.586 |
| Folinic Acid | UAAGG | GCUUGGUACGUUAUAUUCA | -0.585 |
| Folinic Acid | GCUUGGUACGUUAUAUUCA | UAAGA | -0.585 |
| Folinic Acid | UAGGCGGG | GCUUGGUACGUUAUAUUCA | -0.585 |
| Folinic Acid | UAGCAG | GCUUGGUACGUUAUAUUCA | -0.584 |
| Folinic Acid | UAGGAGG | GCUUGGUACGUUAUAUUCA | -0.584 |
| Folinic Acid | UAGGAAGG | GCUUGGUACGUUAUAUUCA | -0.584 |
| Folinic Acid | UGGGGGG | GCUUGGUACGUUAUAUUCA | -0.584 |
| Folinic Acid | UAGGGGG | GCUUGGUACGUUAUAUUCA | -0.584 |
| Folinic Acid | UAUGUG | GCUUGGUACGUUAUAUUCA | -0.583 |
| Folinic Acid | UAUGGGGG | GCUUGGUACGUUAUAUUCA | -0.583 |
| Folinic Acid | UAGGGAG | GCUUGGUACGUUAUAUUCA | -0.582 |
| Folinic Acid | UAAGGG | GCUUGGUACGUUAUAUUCA | -0.582 |
| Folinic Acid | UGUGAG | GCUUGGUACGUUAUAUUCA | -0.582 |
| Folinic Acid | GCUUGGUACGUUAUAUUCA | UAGUG | -0.582 |
| Folinic Acid | GCUUGGUACGUUAUAUUCA | UCGGG | -0.581 |
| Folinic Acid | UAUAGG | GCUUGGUACGUUAUAUUCA | -0.581 |
| Folinic Acid | UAGAGGG | GCUUGGUACGUUAUAUUCA | -0.581 |
| Folinic Acid | UAAGGGG | GCUUGGUACGUUAUAUUCA | -0.580 |
| Folinic Acid | UAGUGGG | GCUUGGUACGUUAUAUUCA | -0.580 |
| Folinic Acid | UAGAUG | GCUUGGUACGUUAUAUUCA | -0.580 |
| Folinic Acid | UGGGG | GCUUGGUACGUUAUAUUCA | -0.579 |
| Folinic Acid | UAGAG | GCUUGGUACGUUAUAUUCA | -0.579 |
| Folinic Acid | UAAGCGGG | GCUUGGUACGUUAUAUUCA | -0.579 |
| Folinic Acid | UGAGGG | GCUUGGUACGUUAUAUUCA | -0.578 |
| Folinic Acid | UAGGG | GCUUGGUACGUUAUAUUCA | -0.578 |
| Folinic Acid | UGAGG | GCUUGGUACGUUAUAUUCA | -0.578 |
| Folinic Acid | UAGGGGAG | GCUUGGUACGUUAUAUUCA | -0.577 |
| Folinic Acid | GCUUGGUACGUUAUAUUCA | UCGAG | -0.577 |
| Folinic Acid | UGAGGGG | GCUUGGUACGUUAUAUUCA | -0.577 |
| Folinic Acid | UGGAGGG | GCUUGGUACGUUAUAUUCA | -0.576 |
| Folinic Acid | UAGAGGG | GCUUGGUACGUUAUAUUCA | -0.575 |
| Folinic Acid | GCUUGGUACGUUAUAUUCA | UAGAA | -0.574 |
| Folinic Acid | UGGGG | GCUUGGUACGUUAUAUUCA | -0.574 |
| Folinic Acid | UAAAGGG | GCUUGGUACGUUAUAUUCA | -0.573 |
| Folinic Acid | UUAGAG | GCUUGGUACGUUAUAUUCA | -0.573 |
| Folinic Acid | UAGUGGGG | GCUUGGUACGUUAUAUUCA | -0.572 |
| Folinic Acid | UAGGG | GCUUGGUACGUUAUAUUCA | -0.571 |
| Folinic Acid | UGAAGGG | GCUUGGUACGUUAUAUUCA | -0.571 |
| Folinic Acid | UAGGUGG | GCUUGGUACGUUAUAUUCA | -0.571 |
| Folinic Acid | UAGGUAGG | GCUUGGUACGUUAUAUUCA | -0.570 |
| Folinic Acid | UAGCGG | GCUUGGUACGUUAUAUUCA | -0.570 |
| Folinic Acid | UGAGGG | GCUUGGUACGUUAUAUUCA | -0.570 |
| Folinic Acid | UAGGAGAG | GCUUGGUACGUUAUAUUCA | -0.570 |
| Folinic Acid | UAGGAAG | GCUUGGUACGUUAUAUUCA | -0.569 |
| Folinic Acid | UAUGGGG | GCUUGGUACGUUAUAUUCA | -0.568 |
| Folinic Acid | UAUUGG | GCUUGGUACGUUAUAUUCA | -0.568 |
| Folinic Acid | UAUGGG | GCUUGGUACGUUAUAUUCA | -0.568 |
| Folinic Acid | UAAGGAG | GCUUGGUACGUUAUAUUCA | -0.566 |
| Folinic Acid | UAUGUGGG | GCUUGGUACGUUAUAUUCA | -0.566 |
| Folinic Acid | UAUAGGG | GCUUGGUACGUUAUAUUCA | -0.566 |
| Folinic Acid | GCUUGGUACGUUAUAUUCA | UGGCG | -0.565 |
| Folinic Acid | UGUGGGG | GCUUGGUACGUUAUAUUCA | -0.565 |
| Folinic Acid | UAGUAGGG | GCUUGGUACGUUAUAUUCA | -0.565 |
| Folinic Acid | UGAUAG | GCUUGGUACGUUAUAUUCA | -0.565 |
| Folinic Acid | UAGGGUGG | GCUUGGUACGUUAUAUUCA | -0.564 |
| Folinic Acid | UAAUAGGG | GCUUGGUACGUUAUAUUCA | -0.564 |
| Folinic Acid | UAAUUG | GCUUGGUACGUUAUAUUCA | -0.564 |
| Folinic Acid | UAAUGGGG | GCUUGGUACGUUAUAUUCA | -0.564 |
| Folinic Acid | UAGGGAAG | GCUUGGUACGUUAUAUUCA | -0.564 |
| Folinic Acid | UGGAGG | GCUUGGUACGUUAUAUUCA | -0.564 |
| Folinic Acid | UAUGGAG | GCUUGGUACGUUAUAUUCA | -0.563 |
| Folinic Acid | UGUGGG | GCUUGGUACGUUAUAUUCA | -0.563 |
| Folinic Acid | UAGGUGAG | GCUUGGUACGUUAUAUUCA | -0.563 |
| Folinic Acid | UAAGGGAG | GCUUGGUACGUUAUAUUCA | -0.563 |
| Folinic Acid | UAAUGGG | GCUUGGUACGUUAUAUUCA | -0.563 |
| Folinic Acid | UAAAUG | GCUUGGUACGUUAUAUUCA | -0.562 |
| Folinic Acid | UAAGAGAG | GCUUGGUACGUUAUAUUCA | -0.562 |
| Folinic Acid | UAAGAAGG | GCUUGGUACGUUAUAUUCA | -0.562 |
| Folinic Acid | GCUUGGUACGUUAUAUUCA | AGAAG | -0.562 |
| Folinic Acid | UAGGAUGG | GCUUGGUACGUUAUAUUCA | -0.561 |
| Folinic Acid | UGUAG | GCUUGGUACGUUAUAUUCA | -0.561 |
| Folinic Acid | UAAAGGG | GCUUGGUACGUUAUAUUCA | -0.561 |
| Folinic Acid | UUAGGG | GCUUGGUACGUUAUAUUCA | -0.561 |
| Folinic Acid | UAGUAGG | GCUUGGUACGUUAUAUUCA | -0.561 |
| Folinic Acid | UGUAGGG | GCUUGGUACGUUAUAUUCA | -0.561 |
| Folinic Acid | UAAGG | GCUUGGUACGUUAUAUUCA | -0.561 |
| Folinic Acid | UGAGG | GCUUGGUACGUUAUAUUCA | -0.561 |
| Folinic Acid | UGGUGG | GCUUGGUACGUUAUAUUCA | -0.560 |
| Folinic Acid | GCUUGGUACGUUAUAUUCA | UUGAG | -0.560 |
| Folinic Acid | UAGGAAAG | GCUUGGUACGUUAUAUUCA | -0.559 |
| Folinic Acid | UAGGUAG | GCUUGGUACGUUAUAUUCA | -0.559 |
| Folinic Acid | UAGAGGGG | GCUUGGUACGUUAUAUUCA | -0.559 |
| Folinic Acid | UAUUGGG | GCUUGGUACGUUAUAUUCA | -0.559 |
| Folinic Acid | UAGGCAGG | GCUUGGUACGUUAUAUUCA | -0.559 |
| Folinic Acid | UUGUAG | GCUUGGUACGUUAUAUUCA | -0.559 |
| Folinic Acid | UAACAG | GCUUGGUACGUUAUAUUCA | -0.558 |
| Folinic Acid | GCUUGGUACGUUAUAUUCA | UACGG | -0.558 |
| Folinic Acid | UAUGCGGG | GCUUGGUACGUUAUAUUCA | -0.558 |
| Folinic Acid | GCUUGGUACGUUAUAUUCA | UGCGG | -0.557 |
| Folinic Acid | UAGUUGGG | GCUUGGUACGUUAUAUUCA | -0.557 |
| Folinic Acid | UAGAGAG | GCUUGGUACGUUAUAUUCA | -0.557 |
| Folinic Acid | UAGAAGG | GCUUGGUACGUUAUAUUCA | -0.557 |
| Folinic Acid | UAAGGAGG | GCUUGGUACGUUAUAUUCA | -0.556 |
| Folinic Acid | UAGUGAG | GCUUGGUACGUUAUAUUCA | -0.556 |
| Folinic Acid | UAAGAGG | GCUUGGUACGUUAUAUUCA | -0.556 |
| Folinic Acid | UAUGAGAG | GCUUGGUACGUUAUAUUCA | -0.555 |
| Folinic Acid | UGGGUG | GCUUGGUACGUUAUAUUCA | -0.555 |
| Folinic Acid | UAUGG | GCUUGGUACGUUAUAUUCA | -0.555 |
| Folinic Acid | UGUGGG | GCUUGGUACGUUAUAUUCA | -0.555 |
| Folinic Acid | GCUUGGUACGUUAUAUUCA | UAUAG | -0.555 |
| Folinic Acid | UAUUAGGG | GCUUGGUACGUUAUAUUCA | -0.555 |
| Folinic Acid | UGAAAG | GCUUGGUACGUUAUAUUCA | -0.555 |
| Folinic Acid | UGGGUG | GCUUGGUACGUUAUAUUCA | -0.554 |
| Folinic Acid | UGGUG | GCUUGGUACGUUAUAUUCA | -0.554 |
| Folinic Acid | UAGGCG | GCUUGGUACGUUAUAUUCA | -0.554 |
| Folinic Acid | UAUAGGG | GCUUGGUACGUUAUAUUCA | -0.554 |
| Folinic Acid | UACGAG | GCUUGGUACGUUAUAUUCA | -0.554 |
| Folinic Acid | UAGGCGAG | GCUUGGUACGUUAUAUUCA | -0.554 |
| Folinic Acid | UGAGUG | GCUUGGUACGUUAUAUUCA | -0.553 |
| Folinic Acid | UAGAAGGG | GCUUGGUACGUUAUAUUCA | -0.553 |
| Folinic Acid | UAGUG | GCUUGGUACGUUAUAUUCA | -0.553 |
| Folinic Acid | UAAUUGGG | GCUUGGUACGUUAUAUUCA | -0.552 |
| Folinic Acid | UAAGUGAG | GCUUGGUACGUUAUAUUCA | -0.552 |
| Folinic Acid | UGGGGUG | GCUUGGUACGUUAUAUUCA | -0.552 |
| Folinic Acid | UAAGUG | GCUUGGUACGUUAUAUUCA | -0.552 |
| Folinic Acid | UUUGAG | GCUUGGUACGUUAUAUUCA | -0.552 |
| Folinic Acid | GCUUGGUACGUUAUAUUCA | UGGUA | -0.551 |
| Folinic Acid | UAUGGGAG | GCUUGGUACGUUAUAUUCA | -0.551 |
| Folinic Acid | UAUGAAGG | GCUUGGUACGUUAUAUUCA | -0.551 |
| Folinic Acid | UGAGGUG | GCUUGGUACGUUAUAUUCA | -0.550 |
| Folinic Acid | UGUGG | GCUUGGUACGUUAUAUUCA | -0.550 |
| Folinic Acid | UAUAG | GCUUGGUACGUUAUAUUCA | -0.550 |
| Folinic Acid | UAGGUG | GCUUGGUACGUUAUAUUCA | -0.550 |
| Folinic Acid | GCUUGGUACGUUAUAUUCA | UGUAA | -0.550 |
| Folinic Acid | UAUUGGGG | GCUUGGUACGUUAUAUUCA | -0.549 |
| Folinic Acid | UAGAGG | GCUUGGUACGUUAUAUUCA | -0.549 |
| Folinic Acid | UGGAGG | GCUUGGUACGUUAUAUUCA | -0.549 |
| Folinic Acid | UAGGUUGG | GCUUGGUACGUUAUAUUCA | -0.549 |
| Folinic Acid | UACGGG | GCUUGGUACGUUAUAUUCA | -0.549 |
| Folinic Acid | UAUGG | GCUUGGUACGUUAUAUUCA | -0.549 |
| Folinic Acid | UAGUGGG | GCUUGGUACGUUAUAUUCA | -0.548 |
| Folinic Acid | UGUUAG | GCUUGGUACGUUAUAUUCA | -0.548 |
| Folinic Acid | UAGAUGG | GCUUGGUACGUUAUAUUCA | -0.548 |
| Folinic Acid | UAGUAAGG | GCUUGGUACGUUAUAUUCA | -0.548 |
| Folinic Acid | GCUUGGUACGUUAUAUUCA | GGAAG | -0.547 |
| Folinic Acid | UUGAAG | GCUUGGUACGUUAUAUUCA | -0.547 |
| Folinic Acid | UAGUCGGG | GCUUGGUACGUUAUAUUCA | -0.547 |
| Folinic Acid | UAGUUGG | GCUUGGUACGUUAUAUUCA | -0.546 |
| Folinic Acid | UGGUGGG | GCUUGGUACGUUAUAUUCA | -0.546 |
| Folinic Acid | UAGGUAAG | GCUUGGUACGUUAUAUUCA | -0.546 |
| Folinic Acid | UGUGG | GCUUGGUACGUUAUAUUCA | -0.546 |
| Neomycin | GCUUGUCCUUUAAUGGUCC | AGGGG | -0.416 |
| Neomycin | GCUUGUCCUUUAAUGGUCC | CGGAA | -0.412 |
| Neomycin | GCUUGUCCUUUAAUGGUCC | AGGAG | -0.401 |
| Neomycin | GCUUGUCCUUUAAUGGUCC | AGGGGG | -0.382 |
| Neomycin | GCUUGUCCUUUAAUGGUCC | AGUGG | -0.373 |
| Neomycin | GCUUGUCCUUUAAUGGUCC | AAGGG | -0.373 |
| Neomycin | GCUUGUCCUUUAAUGGUCC | CAGAA | -0.373 |
| Neomycin | GCUUGUCCUUUAAUGGUCC | CGUAA | -0.370 |
| Neomycin | GCUUGUCCUUUAAUGGUCC | AGAGG | -0.369 |
| Neomycin | GCUUGUCCUUUAAUGGUCC | AAGGGG | -0.367 |
| Neomycin | GCUUGUCCUUUAAUGGUCC | ACGGG | -0.366 |
| Neomycin | GCUUGUCCUUUAAUGGUCC | UGGAG | -0.358 |
| Neomycin | GCUUGUCCUUUAAUGGUCC | AGGAGG | -0.348 |
| Neomycin | GCUUGUCCUUUAAUGGUCC | AAGAG | -0.342 |
| Neomycin | GCUUGUCCUUUAAUGGUCC | AGUAG | -0.339 |
| Neomycin | GCUUGUCCUUUAAUGGUCC | AGAGGG | -0.339 |
| Neomycin | GCUUGUCCUUUAAUGGUCC | ACGAG | -0.334 |
| Neomycin | GCUUGUCCUUUAAUGGUCC | AUGGG | -0.333 |
| Neomycin | GCUUGUCCUUUAAUGGUCC | CGGUA | -0.332 |
| Neomycin | GCUUGUCCUUUAAUGGUCC | UAGGG | -0.331 |
| Neomycin | GCUUGUCCUUUAAUGGUCC | AAGAGG | -0.331 |
| Neomycin | GCUUGUCCUUUAAUGGUCC | CAUAA | -0.328 |
| Neomycin | GCUUGUCCUUUAAUGGUCC | AAUGG | -0.326 |
| Neomycin | GCUUGUCCUUUAAUGGUCC | CGAAA | -0.325 |
| Neomycin | GCUUGUCCUUUAAUGGUCC | AGGAA | -0.325 |
| Neomycin | GCUUGUCCUUUAAUGGUCC | CCGAA | -0.324 |
| Neomycin | GCUUGUCCUUUAAUGGUCC | GGGAG | -0.323 |
| Neomycin | GCUUGUCCUUUAAUGGUCC | CGGCA | -0.321 |
| Neomycin | GCUUGUCCUUUAAUGGUCC | AAAGGG | -0.321 |
| Neomycin | GCUUGUCCUUUAAUGGUCC | GAGGGG | -0.321 |
| Neomycin | GCUUGUCCUUUAAUGGUCC | AGAAG | -0.320 |
| Neomycin | GCUUGUCCUUUAAUGGUCC | UCGAG | -0.319 |
| Neomycin | GCUUGUCCUUUAAUGGUCC | ACUGG | -0.318 |
| Neomycin | GCUUGUCCUUUAAUGGUCC | AUGAG | -0.318 |
| Neomycin | GCUUGUCCUUUAAUGGUCC | AGGUGG | -0.316 |
| Neomycin | GCUUGUCCUUUAAUGGUCC | GCGGG | -0.313 |
| Neomycin | GCUUGUCCUUUAAUGGUCC | UGGAA | -0.313 |
| Neomycin | GCUUGUCCUUUAAUGGUCC | AGAAGG | -0.313 |
| Neomycin | GCUUGUCCUUUAAUGGUCC | AGGCGG | -0.311 |
| Neomycin | GCUUGUCCUUUAAUGGUCC | GAGGG | -0.311 |
| Neomycin | GCUUGUCCUUUAAUGGUCC | CAGUA | -0.311 |
| Neomycin | GCUUGUCCUUUAAUGGUCC | GGUGG | -0.310 |
| Neomycin | GCUUGUCCUUUAAUGGUCC | AGGGA | -0.308 |
| Neomycin | GCUUGUCCUUUAAUGGUCC | AAAGG | -0.305 |
| Neomycin | GCUUGUCCUUUAAUGGUCC | GUGGG | -0.305 |
| Neomycin | GCUUGUCCUUUAAUGGUCC | GGGAGG | -0.303 |
| Neomycin | GCUUGUCCUUUAAUGGUCC | UAGAG | -0.302 |
| Neomycin | GCUUGUCCUUUAAUGGUCC | AUGGGG | -0.300 |
| Neomycin | GCUUGUCCUUUAAUGGUCC | CGUGA | -0.300 |
| Neomycin | GCUUGUCCUUUAAUGGUCC | AAGCGG | -0.297 |
| Neomycin | GCUUGUCCUUUAAUGGUCC | CAGCA | -0.297 |
| Neomycin | GCUUGUCCUUUAAUGGUCC | CGGGAA | -0.297 |
| Neomycin | GCUUGUCCUUUAAUGGUCC | CUGAA | -0.295 |
| Neomycin | GCUUGUCCUUUAAUGGUCC | AAUGGG | -0.295 |
| Neomycin | GCUUGUCCUUUAAUGGUCC | AAGUGG | -0.295 |
| Neomycin | GCUUGUCCUUUAAUGGUCC | AGUGGG | -0.295 |
| Neomycin | GCUUGUCCUUUAAUGGUCC | AAAAGG | -0.295 |
| Neomycin | GCUUGUCCUUUAAUGGUCC | AGAUGG | -0.295 |
| Neomycin | GCUUGUCCUUUAAUGGUCC | CCUAA | -0.295 |
| Neomycin | GCUUGUCCUUUAAUGGUCC | CGUUA | -0.295 |
| Neomycin | GCUUGUCCUUUAAUGGUCC | ACUAG | -0.291 |
| Neomycin | GCUUGUCCUUUAAUGGUCC | AACGGG | -0.290 |
| Neomycin | GCUUGUCCUUUAAUGGUCC | ACAGG | -0.289 |
| Neomycin | GCUUGUCCUUUAAUGGUCC | UGGGG | -0.289 |
| Neomycin | GCUUGUCCUUUAAUGGUCC | UAGGA | -0.288 |
| Neomycin | GCUUGUCCUUUAAUGGUCC | UGAAG | -0.288 |
| Neomycin | GCUUGUCCUUUAAUGGUCC | AGUUGG | -0.288 |
| Neomycin | GCUUGUCCUUUAAUGGUCC | AUUGG | -0.287 |
| Neomycin | GCUUGUCCUUUAAUGGUCC | CAGGAA | -0.287 |
| Neomycin | GCUUGUCCUUUAAUGGUCC | AAUAG | -0.287 |
| Neomycin | GCUUGUCCUUUAAUGGUCC | AGGGGA | -0.286 |
| Neomycin | GCUUGUCCUUUAAUGGUCC | AGUAGG | -0.285 |
| Neomycin | GCUUGUCCUUUAAUGGUCC | AAGCGGG | -0.285 |
| Neomycin | GCUUGUCCUUUAAUGGUCC | AGCGGG | -0.284 |
| Neomycin | GCUUGUCCUUUAAUGGUCC | AACAGG | -0.283 |
| Neomycin | GCUUGUCCUUUAAUGGUCC | AUGAGG | -0.282 |
| Neomycin | GCUUGUCCUUUAAUGGUCC | GAGAGG | -0.282 |
| Neomycin | GCUUGUCCUUUAAUGGUCC | UCGAA | -0.281 |
| Neomycin | GCUUGUCCUUUAAUGGUCC | CGAGA | -0.281 |
| Neomycin | GCUUGUCCUUUAAUGGUCC | CAUGA | -0.281 |
| Neomycin | GCUUGUCCUUUAAUGGUCC | AGGGAG | -0.280 |
| Neomycin | GCUUGUCCUUUAAUGGUCC | AGACGG | -0.280 |
| Neomycin | GCUUGUCCUUUAAUGGUCC | AGCAGG | -0.279 |
| Neomycin | GCUUGUCCUUUAAUGGUCC | AGUAA | -0.278 |
| Neomycin | GCUUGUCCUUUAAUGGUCC | AAGGAG | -0.278 |
| Neomycin | GCUUGUCCUUUAAUGGUCC | UGGGA | -0.278 |
| Neomycin | GCUUGUCCUUUAAUGGUCC | CGAUA | -0.277 |
| Neomycin | GCUUGUCCUUUAAUGGUCC | UAAGG | -0.277 |
| Neomycin | GCUUGUCCUUUAAUGGUCC | AAGGGGG | -0.276 |
| Neomycin | GCUUGUCCUUUAAUGGUCC | AGUGA | -0.275 |
| Neomycin | GCUUGUCCUUUAAUGGUCC | GAAGGG | -0.275 |
| Neomycin | GCUUGUCCUUUAAUGGUCC | CAUUA | -0.274 |
| Neomycin | GCUUGUCCUUUAAUGGUCC | AAUAGG | -0.274 |
| Neomycin | GCUUGUCCUUUAAUGGUCC | CAGGGAA | -0.274 |
| Neomycin | GCUUGUCCUUUAAUGGUCC | AAGAA | -0.273 |
| Neomycin | GCUUGUCCUUUAAUGGUCC | UGAGG | -0.273 |
| Neomycin | GCUUGUCCUUUAAUGGUCC | GGAAGG | -0.272 |
| Neomycin | GCUUGUCCUUUAAUGGUCC | CAAAA | -0.272 |
| Neomycin | GCUUGUCCUUUAAUGGUCC | AUAGG | -0.272 |
| Neomycin | GCUUGUCCUUUAAUGGUCC | GGGUGG | -0.272 |
| Neomycin | GCUUGUCCUUUAAUGGUCC | ACGGGG | -0.271 |
| Neomycin | GCUUGUCCUUUAAUGGUCC | AAUUGG | -0.270 |
| Neomycin | GCUUGUCCUUUAAUGGUCC | GGUAG | -0.269 |
| Neomycin | GCUUGUCCUUUAAUGGUCC | AUUAG | -0.268 |
| Neomycin | GCUUGUCCUUUAAUGGUCC | AGGCG | -0.268 |
| Neomycin | GCUUGUCCUUUAAUGGUCC | AAAUGG | -0.267 |
| Neomycin | GCUUGUCCUUUAAUGGUCC | CAGAGAA | -0.267 |
| Neomycin | GCUUGUCCUUUAAUGGUCC | AAGCAGG | -0.267 |
| Neomycin | GCUUGUCCUUUAAUGGUCC | CAGCGAA | -0.267 |
| Neomycin | GCUUGUCCUUUAAUGGUCC | GAUGG | -0.267 |
| Neomycin | GCUUGUCCUUUAAUGGUCC | GCUGG | -0.267 |
| Neomycin | GCUUGUCCUUUAAUGGUCC | GUGGGG | -0.266 |
| Neomycin | GCUUGUCCUUUAAUGGUCC | AGCUGG | -0.266 |
| Neomycin | GCUUGUCCUUUAAUGGUCC | GCGAG | -0.265 |
| Neomycin | GCUUGUCCUUUAAUGGUCC | AAACGG | -0.265 |
| Neomycin | GCUUGUCCUUUAAUGGUCC | GGGCGG | -0.265 |
| Neomycin | GCUUGUCCUUUAAUGGUCC | AGAGA | -0.264 |
| Neomycin | GCUUGUCCUUUAAUGGUCC | GUGAG | -0.264 |
| Neomycin | GCUUGUCCUUUAAUGGUCC | CGGGGA | -0.263 |
| Neomycin | GCUUGUCCUUUAAUGGUCC | AUGUGG | -0.262 |
| Neomycin | GCUUGUCCUUUAAUGGUCC | UGAGA | -0.262 |
| Neomycin | GCUUGUCCUUUAAUGGUCC | CGGUAA | -0.260 |
| Neomycin | GCUUGUCCUUUAAUGGUCC | AUAGGG | -0.260 |
| Neomycin | GCUUGUCCUUUAAUGGUCC | CUUAA | -0.260 |
| Neomycin | GCUUGUCCUUUAAUGGUCC | AGUCGG | -0.260 |
| Neomycin | GCUUGUCCUUUAAUGGUCC | AGCGG | -0.260 |
| Neomycin | GCUUGUCCUUUAAUGGUCC | UGAAA | -0.260 |
| Neomycin | GCUUGUCCUUUAAUGGUCC | GUUGG | -0.260 |
| Neomycin | GCUUGUCCUUUAAUGGUCC | ACGAGG | -0.259 |
| Neomycin | GCUUGUCCUUUAAUGGUCC | AAGGAGG | -0.259 |
| Neomycin | GCUUGUCCUUUAAUGGUCC | AGGUG | -0.258 |
| Neomycin | GCUUGUCCUUUAAUGGUCC | AAGCGAG | -0.257 |
| Neomycin | GCUUGUCCUUUAAUGGUCC | AAGGGA | -0.257 |
| Neomycin | GCUUGUCCUUUAAUGGUCC | AAGGA | -0.256 |
| Neomycin | GCUUGUCCUUUAAUGGUCC | GGUGGG | -0.255 |
| Neomycin | GCUUGUCCUUUAAUGGUCC | AAUCGG | -0.255 |
| Neomycin | GCUUGUCCUUUAAUGGUCC | UGUAG | -0.255 |
| Neomycin | GCUUGUCCUUUAAUGGUCC | GAGAG | -0.254 |
| Neomycin | GCUUGUCCUUUAAUGGUCC | UCGGAG | -0.253 |
| Neomycin | GCUUGUCCUUUAAUGGUCC | AUGCGG | -0.253 |
| Neomycin | GCUUGUCCUUUAAUGGUCC | AAGAGGG | -0.253 |
| Neomycin | GCUUGUCCUUUAAUGGUCC | CGUUAA | -0.252 |
| Neomycin | GCUUGUCCUUUAAUGGUCC | GGAAG | -0.252 |
| Neomycin | GCUUGUCCUUUAAUGGUCC | GGAUGG | -0.252 |
| Neomycin | GCUUGUCCUUUAAUGGUCC | UAAGA | -0.252 |
| Neomycin | GCUUGUCCUUUAAUGGUCC | GGGAA | -0.251 |
| Neomycin | GCUUGUCCUUUAAUGGUCC | UGGGAG | -0.251 |
| Neomycin | GCUUGUCCUUUAAUGGUCC | AAGGGAG | -0.251 |
| Neomycin | GCUUGUCCUUUAAUGGUCC | AGGGAA | -0.250 |
| Neomycin | GCUUGUCCUUUAAUGGUCC | AGGUAG | -0.250 |
| Neomycin | GCUUGUCCUUUAAUGGUCC | AAGAAGG | -0.250 |
| Neomycin | GCUUGUCCUUUAAUGGUCC | GAUGGG | -0.250 |
| Neomycin | GCUUGUCCUUUAAUGGUCC | AGAGGA | -0.249 |
| Neomycin | GCUUGUCCUUUAAUGGUCC | AUAAGG | -0.249 |
| Neomycin | GCUUGUCCUUUAAUGGUCC | GAAAGG | -0.248 |
| Neomycin | GCUUGUCCUUUAAUGGUCC | AAAAG | -0.248 |
| Neomycin | GCUUGUCCUUUAAUGGUCC | AACUGG | -0.247 |
| Neomycin | GCUUGUCCUUUAAUGGUCC | ACGUGG | -0.247 |
| Neomycin | GCUUGUCCUUUAAUGGUCC | GUGAGG | -0.247 |
| Neomycin | GCUUGUCCUUUAAUGGUCC | GAAGG | -0.247 |
| Neomycin | GCUUGUCCUUUAAUGGUCC | GUAGG | -0.247 |
| Neomycin | GCUUGUCCUUUAAUGGUCC | CAGUAA | -0.247 |
| Neomycin | GCUUGUCCUUUAAUGGUCC | AGAAA | -0.247 |
| Neomycin | GCUUGUCCUUUAAUGGUCC | CCGUA | -0.247 |
| Neomycin | GCUUGUCCUUUAAUGGUCC | GGUUGG | -0.246 |
| Neomycin | GCUUGUCCUUUAAUGGUCC | UGGGGCA | -0.246 |
| Neomycin | GCUUGUCCUUUAAUGGUCC | CAGGGA | -0.246 |
| Neomycin | GCUUGUCCUUUAAUGGUCC | AGGAGA | -0.246 |
| Neomycin | GCUUGUCCUUUAAUGGUCC | AGCAG | -0.245 |
| Neomycin | GCUUGUCCUUUAAUGGUCC | GAGUGG | -0.245 |
| Neomycin | GCUUGUCCUUUAAUGGUCC | ACAAG | -0.245 |
| Neomycin | GCUUGUCCUUUAAUGGUCC | CAAGA | -0.245 |
| Neomycin | GCUUGUCCUUUAAUGGUCC | GACGGG | -0.245 |
| Neomycin | GCUUGUCCUUUAAUGGUCC | CAGGUAA | -0.244 |
| Neomycin | GCUUGUCCUUUAAUGGUCC | GAGCGG | -0.244 |
| Neomycin | GCUUGUCCUUUAAUGGUCC | GGUAGG | -0.243 |
| Neomycin | GCUUGUCCUUUAAUGGUCC | GGCGGG | -0.243 |
| Neomycin | GCUUGUCCUUUAAUGGUCC | CAAUA | -0.243 |
| Neomycin | GCUUGUCCUUUAAUGGUCC | AGGAAG | -0.243 |
| Neomycin | GCUUGUCCUUUAAUGGUCC | CGUGAA | -0.243 |
| Neomycin | GCUUGUCCUUUAAUGGUCC | ACAGGG | -0.242 |
| Neomycin | GCUUGUCCUUUAAUGGUCC | GCAGG | -0.241 |
| Neomycin | GCUUGUCCUUUAAUGGUCC | UAGAA | -0.241 |
| Neomycin | GCUUGUCCUUUAAUGGUCC | UCGGGAA | -0.240 |
| Neomycin | GCUUGUCCUUUAAUGGUCC | GGCAGG | -0.240 |
| Neomycin | GCUUGUCCUUUAAUGGUCC | CGACA | -0.239 |
| Neomycin | GCUUGUCCUUUAAUGGUCC | AGGCAGG | -0.239 |
| Neomycin | GCUUGUCCUUUAAUGGUCC | AAGAAG | -0.239 |
| Neomycin | GCUUGUCCUUUAAUGGUCC | CUGUA | -0.238 |
| Neomycin | GCUUGUCCUUUAAUGGUCC | CGAGAA | -0.237 |
| Neomycin | GCUUGUCCUUUAAUGGUCC | CAGAAA | -0.237 |
| Neomycin | GCUUGUCCUUUAAUGGUCC | CGGAAA | -0.237 |
| Neomycin | GCUUGUCCUUUAAUGGUCC | AAGUAG | -0.237 |
| Neomycin | GCUUGUCCUUUAAUGGUCC | CAGAAAA | -0.237 |
| Neomycin | GCUUGUCCUUUAAUGGUCC | CGCAA | -0.237 |
| Neomycin | GCUUGUCCUUUAAUGGUCC | AACCAGG | -0.236 |
| Neomycin | GCUUGUCCUUUAAUGGUCC | GACAGG | -0.236 |
| Neomycin | GCUUGUCCUUUAAUGGUCC | AGGGGGG | -0.236 |
| Neomycin | GCUUGUCCUUUAAUGGUCC | AUAAG | -0.236 |
| Neomycin | GCUUGUCCUUUAAUGGUCC | CGGUUA | -0.236 |
| Theophylline | AUACCAGCAUCGUCUUGAUGCCCUUGGCAG | UGGAG | -0.849 |
| Theophylline | AUACCAGCAUCGUCUUGAUGCCCUUGGCAG | UCGAG | -0.799 |
| Theophylline | AUACCAGCAUCGUCUUGAUGCCCUUGGCAG | UGGGG | -0.797 |
| Theophylline | AUACCAGCAUCGUCUUGAUGCCCUUGGCAG | UGGAA | -0.743 |
| Theophylline | AUACCAGCAUCGUCUUGAUGCCCUUGGCAG | UGAAG | -0.742 |
| Theophylline | AUACCAGCAUCGUCUUGAUGCCCUUGGCAG | UCGGG | -0.722 |
| Theophylline | AUACCAGCAUCGUCUUGAUGCCCUUGGCAG | UAGAG | -0.713 |
| Theophylline | AUACCAGCAUCGUCUUGAUGCCCUUGGCAG | UGGGA | -0.710 |
| Theophylline | AUACCAGCAUCGUCUUGAUGCCCUUGGCAG | UAGGG | -0.706 |
| Theophylline | AUACCAGCAUCGUCUUGAUGCCCUUGGCAG | UCGAA | -0.706 |
| Theophylline | AUACCAGCAUCGUCUUGAUGCCCUUGGCAG | UGAGG | -0.686 |
| Theophylline | AUACCAGCAUCGUCUUGAUGCCCUUGGCAG | UCAAG | -0.683 |
| Theophylline | AAAUGGG | AUACCAGCAUCGUCUUGAUGCCCUUGGCAG | -0.675 |
| Theophylline | AUAUGGG | AUACCAGCAUCGUCUUGAUGCCCUUGGCAG | -0.674 |
| Theophylline | AUACCAGCAUCGUCUUGAUGCCCUUGGCAG | AAGGGA | -0.672 |
| Theophylline | AUACCAGCAUCGUCUUGAUGCCCUUGGCAG | UGGCG | -0.665 |
| Theophylline | AUACCAGCAUCGUCUUGAUGCCCUUGGCAG | CGGAG | -0.655 |
| Theophylline | AUACCAGCAUCGUCUUGAUGCCCUUGGCAG | UGAAA | -0.652 |
| Theophylline | AUACCAGCAUCGUCUUGAUGCCCUUGGCAG | UGUAG | -0.650 |
| Theophylline | AUACCAGCAUCGUCUUGAUGCCCUUGGCAG | UGGCA | -0.646 |
| Theophylline | AAUUGGG | AUACCAGCAUCGUCUUGAUGCCCUUGGCAG | -0.643 |
| Theophylline | AUACCAGCAUCGUCUUGAUGCCCUUGGCAG | UCGGA | -0.643 |
| Theophylline | AUACCAGCAUCGUCUUGAUGCCCUUGGCAG | CGGGG | -0.636 |
| Theophylline | AUACCAGCAUCGUCUUGAUGCCCUUGGCAG | AAGUGA | -0.634 |
| Theophylline | AUUUGGG | AUACCAGCAUCGUCUUGAUGCCCUUGGCAG | -0.626 |
| Theophylline | AUACCAGCAUCGUCUUGAUGCCCUUGGCAG | UGUGG | -0.623 |
| Theophylline | AAAUGAG | AUACCAGCAUCGUCUUGAUGCCCUUGGCAG | -0.622 |
| Theophylline | GAAUGGG | AUACCAGCAUCGUCUUGAUGCCCUUGGCAG | -0.620 |
| Theophylline | AUACCAGCAUCGUCUUGAUGCCCUUGGCAG | AGGGGA | -0.617 |
| Theophylline | AAAUGUG | AUACCAGCAUCGUCUUGAUGCCCUUGGCAG | -0.617 |
| Theophylline | AUACCAGCAUCGUCUUGAUGCCCUUGGCAG | UAAGGGG | -0.617 |
| Theophylline | AUAUAGG | AUACCAGCAUCGUCUUGAUGCCCUUGGCAG | -0.617 |
| Theophylline | AAAGGGG | AUACCAGCAUCGUCUUGAUGCCCUUGGCAG | -0.616 |
| Theophylline | AUAUUGG | AUACCAGCAUCGUCUUGAUGCCCUUGGCAG | -0.615 |
| Theophylline | UAAUGGU | AUACCAGCAUCGUCUUGAUGCCCUUGGCAG | -0.615 |
| Theophylline | AAUUAGG | AUACCAGCAUCGUCUUGAUGCCCUUGGCAG | -0.615 |
| Theophylline | AUACCAGCAUCGUCUUGAUGCCCUUGGCAG | UAGGGGG | -0.614 |
| Theophylline | AUACCAGCAUCGUCUUGAUGCCCUUGGCAG | UGAGA | -0.614 |
| Theophylline | AUACCAGCAUCGUCUUGAUGCCCUUGGCAG | UAAAG | -0.612 |
| Theophylline | AUAGGGG | AUACCAGCAUCGUCUUGAUGCCCUUGGCAG | -0.610 |
| Theophylline | AUAUGUG | AUACCAGCAUCGUCUUGAUGCCCUUGGCAG | -0.610 |
| Theophylline | AUACCAGCAUCGUCUUGAUGCCCUUGGCAG | AUGGGA | -0.608 |
| Theophylline | AAAUAGG | AUACCAGCAUCGUCUUGAUGCCCUUGGCAG | -0.606 |
| Theophylline | AAUGGGG | AUACCAGCAUCGUCUUGAUGCCCUUGGCAG | -0.606 |
| Theophylline | AUACCAGCAUCGUCUUGAUGCCCUUGGCAG | UUGGG | -0.605 |
| Theophylline | AUACCAGCAUCGUCUUGAUGCCCUUGGCAG | UCAAA | -0.604 |
| Theophylline | GAAUGAG | AUACCAGCAUCGUCUUGAUGCCCUUGGCAG | -0.603 |
| Theophylline | AAAUUGG | AUACCAGCAUCGUCUUGAUGCCCUUGGCAG | -0.603 |
| Theophylline | AUACCAGCAUCGUCUUGAUGCCCUUGGCAG | UGGUG | -0.603 |
| Theophylline | AUACCAGCAUCGUCUUGAUGCCCUUGGCAG | AAGCGA | -0.602 |
| Theophylline | AUUUAGG | AUACCAGCAUCGUCUUGAUGCCCUUGGCAG | -0.600 |
| Theophylline | AUAUGAG | AUACCAGCAUCGUCUUGAUGCCCUUGGCAG | -0.600 |
| Theophylline | AUACCAGCAUCGUCUUGAUGCCCUUGGCAG | CCGAG | -0.600 |
| Theophylline | AUACCAGCAUCGUCUUGAUGCCCUUGGCAG | AAGAGA | -0.599 |
| Theophylline | AUACCAGCAUCGUCUUGAUGCCCUUGGCAG | UAGGA | -0.595 |
| Theophylline | AUACCAGCAUCGUCUUGAUGCCCUUGGCAG | UAAGG | -0.595 |
| Theophylline | UAUUGGU | AUACCAGCAUCGUCUUGAUGCCCUUGGCAG | -0.594 |
| Theophylline | AAUUGAG | AUACCAGCAUCGUCUUGAUGCCCUUGGCAG | -0.594 |
| Theophylline | AUACCAGCAUCGUCUUGAUGCCCUUGGCAG | UAGAA | -0.592 |
| Theophylline | AUACCAGCAUCGUCUUGAUGCCCUUGGCAG | ACGGGA | -0.592 |
| Theophylline | AUACCAGCAUCGUCUUGAUGCCCUUGGCAG | CAGGUA | -0.592 |
| Theophylline | GAUUGGG | AUACCAGCAUCGUCUUGAUGCCCUUGGCAG | -0.591 |
| Theophylline | AUACCAGCAUCGUCUUGAUGCCCUUGGCAG | UCUAG | -0.591 |
| Theophylline | AUACCAGCAUCGUCUUGAUGCCCUUGGCAG | AACGGA | -0.587 |
| Theophylline | AUACCAGCAUCGUCUUGAUGCCCUUGGCAG | AAUGGA | -0.586 |
| Theophylline | AUACCAGCAUCGUCUUGAUGCCCUUGGCAG | UAGCG | -0.586 |
| Theophylline | AUACCAGCAUCGUCUUGAUGCCCUUGGCAG | UCAGG | -0.584 |
| Theophylline | AUACCAGCAUCGUCUUGAUGCCCUUGGCAG | UGUAA | -0.582 |
| Theophylline | AUACCAGCAUCGUCUUGAUGCCCUUGGCAG | UCGGAA | -0.582 |
| Theophylline | UAAUGGC | AUACCAGCAUCGUCUUGAUGCCCUUGGCAG | -0.582 |
| Theophylline | GAUUGAG | AUACCAGCAUCGUCUUGAUGCCCUUGGCAG | -0.582 |
| Theophylline | AUACCAGCAUCGUCUUGAUGCCCUUGGCAG | UAGGCGG | -0.581 |
| Theophylline | AUACCAGCAUCGUCUUGAUGCCCUUGGCAG | AGGUGA | -0.581 |
| Theophylline | AUACCAGCAUCGUCUUGAUGCCCUUGGCAG | UUGAG | -0.580 |
| Theophylline | AUUGGGG | AUACCAGCAUCGUCUUGAUGCCCUUGGCAG | -0.580 |
| Theophylline | AUACCAGCAUCGUCUUGAUGCCCUUGGCAG | UCGCAA | -0.580 |
| Theophylline | AUACCAGCAUCGUCUUGAUGCCCUUGGCAG | UCGCAG | -0.577 |
| Theophylline | AUACCAGCAUCGUCUUGAUGCCCUUGGCAG | AAGGUA | -0.576 |
| Theophylline | AAUUGUG | AUACCAGCAUCGUCUUGAUGCCCUUGGCAG | -0.575 |
| Theophylline | AUACCAGCAUCGUCUUGAUGCCCUUGGCAG | UCGGAG | -0.575 |
| Theophylline | AUACCAGCAUCGUCUUGAUGCCCUUGGCAG | UGUGA | -0.575 |
| Theophylline | GAUUAGG | AUACCAGCAUCGUCUUGAUGCCCUUGGCAG | -0.574 |
| Theophylline | AAAAGGG | AUACCAGCAUCGUCUUGAUGCCCUUGGCAG | -0.573 |
| Theophylline | GAAUGUG | AUACCAGCAUCGUCUUGAUGCCCUUGGCAG | -0.573 |
| Theophylline | AUACCAGCAUCGUCUUGAUGCCCUUGGCAG | CAGUUA | -0.571 |
| Theophylline | AAUGAGG | AUACCAGCAUCGUCUUGAUGCCCUUGGCAG | -0.571 |
| Theophylline | AUACCAGCAUCGUCUUGAUGCCCUUGGCAG | UAAGCGG | -0.570 |
| Theophylline | AUACCAGCAUCGUCUUGAUGCCCUUGGCAG | GAGGGA | -0.570 |
| Theophylline | AAACGGG | AUACCAGCAUCGUCUUGAUGCCCUUGGCAG | -0.570 |
| Theophylline | AUACCAGCAUCGUCUUGAUGCCCUUGGCAG | AAAGGA | -0.570 |
| Theophylline | AUACCAGCAUCGUCUUGAUGCCCUUGGCAG | CCGGG | -0.570 |
| Theophylline | AGAUGGG | AUACCAGCAUCGUCUUGAUGCCCUUGGCAG | -0.570 |
| Theophylline | AUACCAGCAUCGUCUUGAUGCCCUUGGCAG | CGGGUA | -0.569 |
| Theophylline | GAAGGGG | AUACCAGCAUCGUCUUGAUGCCCUUGGCAG | -0.568 |
| Theophylline | AAAGGAG | AUACCAGCAUCGUCUUGAUGCCCUUGGCAG | -0.567 |
| Theophylline | AAUUUGG | AUACCAGCAUCGUCUUGAUGCCCUUGGCAG | -0.567 |
| Theophylline | UAAUGUU | AUACCAGCAUCGUCUUGAUGCCCUUGGCAG | -0.567 |
| Theophylline | UAUUGGC | AUACCAGCAUCGUCUUGAUGCCCUUGGCAG | -0.565 |
| Theophylline | AUACCAGCAUCGUCUUGAUGCCCUUGGCAG | UAGGGAG | -0.565 |
| Theophylline | AUACCAGCAUCGUCUUGAUGCCCUUGGCAG | CGGUUA | -0.565 |
| Theophylline | AUAAGGG | AUACCAGCAUCGUCUUGAUGCCCUUGGCAG | -0.564 |
| Theophylline | AUGUGGG | AUACCAGCAUCGUCUUGAUGCCCUUGGCAG | -0.563 |
| Theophylline | GAAUAGG | AUACCAGCAUCGUCUUGAUGCCCUUGGCAG | -0.563 |
| Theophylline | AUACCAGCAUCGUCUUGAUGCCCUUGGCAG | AUGUGA | -0.562 |
| Theophylline | AAUAGGG | AUACCAGCAUCGUCUUGAUGCCCUUGGCAG | -0.562 |
| Theophylline | AUACCAGCAUCGUCUUGAUGCCCUUGGCAG | CAGGG | -0.561 |
| Theophylline | GUAUGGG | AUACCAGCAUCGUCUUGAUGCCCUUGGCAG | -0.561 |
| Theophylline | AUAGUGG | AUACCAGCAUCGUCUUGAUGCCCUUGGCAG | -0.561 |
| Theophylline | AUACCAGCAUCGUCUUGAUGCCCUUGGCAG | AGGGUA | -0.561 |
| Theophylline | AAUGGAG | AUACCAGCAUCGUCUUGAUGCCCUUGGCAG | -0.561 |
| Theophylline | AAAUUAG | AUACCAGCAUCGUCUUGAUGCCCUUGGCAG | -0.560 |
| Theophylline | AUACGGG | AUACCAGCAUCGUCUUGAUGCCCUUGGCAG | -0.560 |
| Theophylline | GAUUAAG | AUACCAGCAUCGUCUUGAUGCCCUUGGCAG | -0.559 |
| Theophylline | UUAUGGU | AUACCAGCAUCGUCUUGAUGCCCUUGGCAG | -0.559 |
| Theophylline | AUACCAGCAUCGUCUUGAUGCCCUUGGCAG | CAGGGA | -0.559 |
| Theophylline | AAUUAAG | AUACCAGCAUCGUCUUGAUGCCCUUGGCAG | -0.559 |
| Theophylline | GAUGGGG | AUACCAGCAUCGUCUUGAUGCCCUUGGCAG | -0.559 |
| Theophylline | AAAGGUG | AUACCAGCAUCGUCUUGAUGCCCUUGGCAG | -0.559 |
| Theophylline | UAAUGUC | AUACCAGCAUCGUCUUGAUGCCCUUGGCAG | -0.559 |
| Theophylline | AUACCAGCAUCGUCUUGAUGCCCUUGGCAG | UAUGGGG | -0.559 |
| Theophylline | AAAUUUG | AUACCAGCAUCGUCUUGAUGCCCUUGGCAG | -0.558 |
| Theophylline | AUAUUUG | AUACCAGCAUCGUCUUGAUGCCCUUGGCAG | -0.558 |
| Theophylline | AAGUGGG | AUACCAGCAUCGUCUUGAUGCCCUUGGCAG | -0.558 |
| Theophylline | AUACCAGCAUCGUCUUGAUGCCCUUGGCAG | CAGCUA | -0.557 |
| Theophylline | AAAUAAG | AUACCAGCAUCGUCUUGAUGCCCUUGGCAG | -0.557 |
| Theophylline | AUACCAGCAUCGUCUUGAUGCCCUUGGCAG | UCGCG | -0.556 |
| Theophylline | AUAGAGG | AUACCAGCAUCGUCUUGAUGCCCUUGGCAG | -0.555 |
| Theophylline | GAAUAAG | AUACCAGCAUCGUCUUGAUGCCCUUGGCAG | -0.555 |
| Theophylline | AUACCAGCAUCGUCUUGAUGCCCUUGGCAG | UAGGCAG | -0.554 |
| Theophylline | AUUUUGG | AUACCAGCAUCGUCUUGAUGCCCUUGGCAG | -0.553 |
| Theophylline | AUUGAGG | AUACCAGCAUCGUCUUGAUGCCCUUGGCAG | -0.553 |
| Theophylline | AUACCAGCAUCGUCUUGAUGCCCUUGGCAG | CGAAG | -0.553 |
| Theophylline | GAUGGAG | AUACCAGCAUCGUCUUGAUGCCCUUGGCAG | -0.552 |
| Theophylline | GAAGGAG | AUACCAGCAUCGUCUUGAUGCCCUUGGCAG | -0.552 |
| Theophylline | UAAGGGU | AUACCAGCAUCGUCUUGAUGCCCUUGGCAG | -0.551 |
| Theophylline | AUUUGAG | AUACCAGCAUCGUCUUGAUGCCCUUGGCAG | -0.551 |
| Theophylline | AGAUGUG | AUACCAGCAUCGUCUUGAUGCCCUUGGCAG | -0.551 |
| Theophylline | AGAUGAG | AUACCAGCAUCGUCUUGAUGCCCUUGGCAG | -0.551 |
| Theophylline | GAAUUAG | AUACCAGCAUCGUCUUGAUGCCCUUGGCAG | -0.551 |
| Theophylline | AUACCAGCAUCGUCUUGAUGCCCUUGGCAG | UAAGGGA | -0.550 |
| Theophylline | AAAGUGG | AUACCAGCAUCGUCUUGAUGCCCUUGGCAG | -0.550 |
| Theophylline | AAAUAUG | AUACCAGCAUCGUCUUGAUGCCCUUGGCAG | -0.550 |
| Theophylline | AAAGAGG | AUACCAGCAUCGUCUUGAUGCCCUUGGCAG | -0.548 |
| Theophylline | AUAUUAG | AUACCAGCAUCGUCUUGAUGCCCUUGGCAG | -0.548 |
| Theophylline | AUACCAGCAUCGUCUUGAUGCCCUUGGCAG | UAGGGGA | -0.548 |
| Theophylline | AUACCAGCAUCGUCUUGAUGCCCUUGGCAG | ACGUGA | -0.548 |
| Theophylline | AUACCAGCAUCGUCUUGAUGCCCUUGGCAG | CAGGAA | -0.548 |
| Theophylline | GAAUUGG | AUACCAGCAUCGUCUUGAUGCCCUUGGCAG | -0.547 |
| Theophylline | GGAUGAG | AUACCAGCAUCGUCUUGAUGCCCUUGGCAG | -0.547 |
| Theophylline | AUAUAUG | AUACCAGCAUCGUCUUGAUGCCCUUGGCAG | -0.547 |
| Theophylline | AGUUAGG | AUACCAGCAUCGUCUUGAUGCCCUUGGCAG | -0.547 |
| Theophylline | AUACCAGCAUCGUCUUGAUGCCCUUGGCAG | AAGUUA | -0.547 |
| Theophylline | AUACCAGCAUCGUCUUGAUGCCCUUGGCAG | CGGGAA | -0.546 |
| Theophylline | AUACCAGCAUCGUCUUGAUGCCCUUGGCAG | AGGUUA | -0.546 |
| Theophylline | AGAUAGG | AUACCAGCAUCGUCUUGAUGCCCUUGGCAG | -0.546 |
| Theophylline | AUACCAGCAUCGUCUUGAUGCCCUUGGCAG | UAAGGAG | -0.546 |
| Theophylline | UAUGGGU | AUACCAGCAUCGUCUUGAUGCCCUUGGCAG | -0.544 |
| Theophylline | AUACCAGCAUCGUCUUGAUGCCCUUGGCAG | UGGGAG | -0.544 |
| Theophylline | AUAGGUG | AUACCAGCAUCGUCUUGAUGCCCUUGGCAG | -0.543 |
| Theophylline | AUACCAGCAUCGUCUUGAUGCCCUUGGCAG | UAUGG | -0.543 |
| Theophylline | AAUGUGG | AUACCAGCAUCGUCUUGAUGCCCUUGGCAG | -0.542 |
| Theophylline | AUAGGAG | AUACCAGCAUCGUCUUGAUGCCCUUGGCAG | -0.541 |
| Theophylline | AUACCAGCAUCGUCUUGAUGCCCUUGGCAG | CGGCUA | -0.541 |
| Theophylline | UAUUAGU | AUACCAGCAUCGUCUUGAUGCCCUUGGCAG | -0.540 |
| Theophylline | UAAUGAU | AUACCAGCAUCGUCUUGAUGCCCUUGGCAG | -0.540 |
| Theophylline | AUAUAAG | AUACCAGCAUCGUCUUGAUGCCCUUGGCAG | -0.540 |
| Theophylline | AUUUGUG | AUACCAGCAUCGUCUUGAUGCCCUUGGCAG | -0.538 |
| Theophylline | AAUCGGG | AUACCAGCAUCGUCUUGAUGCCCUUGGCAG | -0.538 |
| Theophylline | UAAUUGU | AUACCAGCAUCGUCUUGAUGCCCUUGGCAG | -0.538 |
| Theophylline | AUACCAGCAUCGUCUUGAUGCCCUUGGCAG | UAGCA | -0.538 |
| Theophylline | AUACCAGCAUCGUCUUGAUGCCCUUGGCAG | AGGGAA | -0.538 |
| Theophylline | AUACCAGCAUCGUCUUGAUGCCCUUGGCAG | AGUGGA | -0.537 |
| Theophylline | AUACCAGCAUCGUCUUGAUGCCCUUGGCAG | CAGAG | -0.536 |
| Theophylline | AUACCAGCAUCGUCUUGAUGCCCUUGGCAG | UCGCA | -0.536 |
| Theophylline | AUACCAGCAUCGUCUUGAUGCCCUUGGCAG | AGGAGA | -0.536 |
| Theophylline | AUACCAGCAUCGUCUUGAUGCCCUUGGCAG | UCUAA | -0.536 |
| Theophylline | AUUAGGG | AUACCAGCAUCGUCUUGAUGCCCUUGGCAG | -0.536 |
| Theophylline | AGUUGGG | AUACCAGCAUCGUCUUGAUGCCCUUGGCAG | -0.535 |
| Theophylline | GAUGAGG | AUACCAGCAUCGUCUUGAUGCCCUUGGCAG | -0.535 |
| Theophylline | AUACCAGCAUCGUCUUGAUGCCCUUGGCAG | AAGCUA | -0.535 |
| Theophylline | GAUUGUG | AUACCAGCAUCGUCUUGAUGCCCUUGGCAG | -0.535 |
| Theophylline | GGAUGGG | AUACCAGCAUCGUCUUGAUGCCCUUGGCAG | -0.535 |
| Theophylline | AUACCAGCAUCGUCUUGAUGCCCUUGGCAG | AGGGCA | -0.535 |
| Theophylline | AAUGGUG | AUACCAGCAUCGUCUUGAUGCCCUUGGCAG | -0.535 |
| Theophylline | AAGUGAG | AUACCAGCAUCGUCUUGAUGCCCUUGGCAG | -0.534 |
| Theophylline | AUACCAGCAUCGUCUUGAUGCCCUUGGCAG | GAGUGA | -0.534 |
| Theophylline | AUACCAGCAUCGUCUUGAUGCCCUUGGCAG | UCGAAG | -0.534 |
| Theophylline | AAAAGAG | AUACCAGCAUCGUCUUGAUGCCCUUGGCAG | -0.534 |
| Theophylline | AUACCAGCAUCGUCUUGAUGCCCUUGGCAG | UAGCGG | -0.533 |
| Theophylline | AAUUAUG | AUACCAGCAUCGUCUUGAUGCCCUUGGCAG | -0.533 |
| Theophylline | GGAUAAG | AUACCAGCAUCGUCUUGAUGCCCUUGGCAG | -0.533 |
| Theophylline | AUACCAGCAUCGUCUUGAUGCCCUUGGCAG | UAAUGGG | -0.533 |
| Theophylline | AUACCAGCAUCGUCUUGAUGCCCUUGGCAG | AUUGGA | -0.532 |
| Theophylline | AUGUAGG | AUACCAGCAUCGUCUUGAUGCCCUUGGCAG | -0.532 |
| Theophylline | AUACCAGCAUCGUCUUGAUGCCCUUGGCAG | UAAAGGG | -0.531 |
| Theophylline | GAAAGGG | AUACCAGCAUCGUCUUGAUGCCCUUGGCAG | -0.531 |
| Theophylline | UAUUGUU | AUACCAGCAUCGUCUUGAUGCCCUUGGCAG | -0.531 |
| Theophylline | AUACCAGCAUCGUCUUGAUGCCCUUGGCAG | AGAGGA | -0.531 |
| Theophylline | GGUUAAG | AUACCAGCAUCGUCUUGAUGCCCUUGGCAG | -0.530 |
| Theophylline | AUACCAGCAUCGUCUUGAUGCCCUUGGCAG | UCGAAA | -0.530 |
| Tetracycline | AAAACAUACCAGAUUUCGAUCUGGAGAGGUGAAGAAUUCGACCACCU | AGGAA | -0.592 |
| Tetracycline | AAAACAUACCAGAUUUCGAUCUGGAGAGGUGAAGAAUUCGACCACCU | AGGGA | -0.574 |
| Tetracycline | AAAACAUACCAGAUUUCGAUCUGGAGAGGUGAAGAAUUCGACCACCU | AGGAG | -0.564 |
| Tetracycline | AAAACAUACCAGAUUUCGAUCUGGAGAGGUGAAGAAUUCGACCACCU | AGGGGGAA | -0.562 |
| Tetracycline | AAAACAUACCAGAUUUCGAUCUGGAGAGGUGAAGAAUUCGACCACCU | AAGAA | -0.562 |
| Tetracycline | AAAACAUACCAGAUUUCGAUCUGGAGAGGUGAAGAAUUCGACCACCU | AAGGA | -0.550 |
| Tetracycline | AAAACAUACCAGAUUUCGAUCUGGAGAGGUGAAGAAUUCGACCACCU | AGGGAA | -0.546 |
| Tetracycline | AAAACAUACCAGAUUUCGAUCUGGAGAGGUGAAGAAUUCGACCACCU | AGAAA | -0.542 |
| Tetracycline | AAAACAUACCAGAUUUCGAUCUGGAGAGGUGAAGAAUUCGACCACCU | AGGGGUAA | -0.542 |
| Tetracycline | AAAACAUACCAGAUUUCGAUCUGGAGAGGUGAAGAAUUCGACCACCU | AGGGUGAA | -0.541 |
| Tetracycline | AAAACAUACCAGAUUUCGAUCUGGAGAGGUGAAGAAUUCGACCACCU | AGGGAUAA | -0.539 |
| Tetracycline | AAAACAUACCAGAUUUCGAUCUGGAGAGGUGAAGAAUUCGACCACCU | AGGGGAAA | -0.539 |
| Tetracycline | AAAACAUACCAGAUUUCGAUCUGGAGAGGUGAAGAAUUCGACCACCU | AGGGUGGA | -0.538 |
| Tetracycline | AAAACAUACCAGAUUUCGAUCUGGAGAGGUGAAGAAUUCGACCACCU | AGGGAAAA | -0.538 |
| Tetracycline | AAAACAUACCAGAUUUCGAUCUGGAGAGGUGAAGAAUUCGACCACCU | AGGGGUGA | -0.537 |
| Tetracycline | AAAACAUACCAGAUUUCGAUCUGGAGAGGUGAAGAAUUCGACCACCU | AGGGGAA | -0.537 |
| Tetracycline | AAAACAUACCAGAUUUCGAUCUGGAGAGGUGAAGAAUUCGACCACCU | UGGAG | -0.535 |
| Tetracycline | AAAACAUACCAGAUUUCGAUCUGGAGAGGUGAAGAAUUCGACCACCU | AGGGGA | -0.535 |
| Tetracycline | AAAACAUACCAGAUUUCGAUCUGGAGAGGUGAAGAAUUCGACCACCU | AGGAUGAA | -0.535 |
| Tetracycline | AAAACAUACCAGAUUUCGAUCUGGAGAGGUGAAGAAUUCGACCACCU | AAGGAGAA | -0.534 |
| Tetracycline | AAAACAUACCAGAUUUCGAUCUGGAGAGGUGAAGAAUUCGACCACCU | AGGUAGAA | -0.534 |
| Tetracycline | AAAACAUACCAGAUUUCGAUCUGGAGAGGUGAAGAAUUCGACCACCU | AGGCAGAA | -0.533 |
| Tetracycline | AAAACAUACCAGAUUUCGAUCUGGAGAGGUGAAGAAUUCGACCACCU | AGGGAUGA | -0.532 |
| Tetracycline | AAAACAUACCAGAUUUCGAUCUGGAGAGGUGAAGAAUUCGACCACCU | AGGCAGGA | -0.532 |
| Tetracycline | AAAACAUACCAGAUUUCGAUCUGGAGAGGUGAAGAAUUCGACCACCU | AGGAGAAA | -0.532 |
| Tetracycline | AAAACAUACCAGAUUUCGAUCUGGAGAGGUGAAGAAUUCGACCACCU | AGGACGAA | -0.532 |
| Tetracycline | AAAACAUACCAGAUUUCGAUCUGGAGAGGUGAAGAAUUCGACCACCU | AGGGGAGA | -0.531 |
| Tetracycline | AAAACAUACCAGAUUUCGAUCUGGAGAGGUGAAGAAUUCGACCACCU | AAGGGGAA | -0.531 |
| Tetracycline | AAAACAUACCAGAUUUCGAUCUGGAGAGGUGAAGAAUUCGACCACCU | AGGGCGAA | -0.531 |
| Tetracycline | AAAACAUACCAGAUUUCGAUCUGGAGAGGUGAAGAAUUCGACCACCU | AGGUAGGA | -0.531 |
| Tetracycline | AAAACAUACCAGAUUUCGAUCUGGAGAGGUGAAGAAUUCGACCACCU | AGGGAAGA | -0.529 |
| Tetracycline | AAAACAUACCAGAUUUCGAUCUGGAGAGGUGAAGAAUUCGACCACCU | AGGAAAAA | -0.529 |
| Tetracycline | AAAACAUACCAGAUUUCGAUCUGGAGAGGUGAAGAAUUCGACCACCU | AAGAAGAA | -0.527 |
| Tetracycline | AAAACAUACCAGAUUUCGAUCUGGAGAGGUGAAGAAUUCGACCACCU | AAGGAGGA | -0.527 |
| Tetracycline | AAAACAUACCAGAUUUCGAUCUGGAGAGGUGAAGAAUUCGACCACCU | AAGGGGGA | -0.527 |
| Tetracycline | AAAACAUACCAGAUUUCGAUCUGGAGAGGUGAAGAAUUCGACCACCU | AAAACAUACCAGAUUUCGAUCUGGAGAGGUGAAGAAUUCGACCACCU | -0.526 |
| Tetracycline | AAAACAUACCAGAUUUCGAUCUGGAGAGGUGAAGAAUUCGACCACCU | AAGAGGAA | -0.526 |
| Tetracycline | AAAACAUACCAGAUUUCGAUCUGGAGAGGUGAAGAAUUCGACCACCU | AGGGCGGA | -0.525 |
| Tetracycline | AAAACAUACCAGAUUUCGAUCUGGAGAGGUGAAGAAUUCGACCACCU | AGGAGAGA | -0.524 |
| Tetracycline | AAAACAUACCAGAUUUCGAUCUGGAGAGGUGAAGAAUUCGACCACCU | AAAACAUACCAGAUUUCGAUCUGGAGAGGUGAAGAAUUCGACCACCU | -0.523 |
| Tetracycline | AAAACAUACCAGAUUUCGAUCUGGAGAGGUGAAGAAUUCGACCACCU | AGAGA | -0.523 |
| Tetracycline | AAAACAUACCAGAUUUCGAUCUGGAGAGGUGAAGAAUUCGACCACCU | AGGAAAGA | -0.522 |
| Tetracycline | AAAACAUACCAGAUUUCGAUCUGGAGAGGUGAAGAAUUCGACCACCU | AGGAGUAA | -0.522 |
| Tetracycline | AAAACAUACCAGAUUUCGAUCUGGAGAGGUGAAGAAUUCGACCACCU | GAGAG | -0.521 |
| Tetracycline | AAAACAUACCAGAUUUCGAUCUGGAGAGGUGAAGAAUUCGACCACCU | AAAACAUACCAGAUUUCGAUCUGGAGAGGUGAAGAAUUCGACCACCU | -0.520 |
| Tetracycline | AAAACAUACCAGAUUUCGAUCUGGAGAGGUGAAGAAUUCGACCACCU | AGGUGGAA | -0.520 |
| Tetracycline | AAAACAUACCAGAUUUCGAUCUGGAGAGGUGAAGAAUUCGACCACCU | AUGGAGAA | -0.520 |
| Tetracycline | AAAACAUACCAGAUUUCGAUCUGGAGAGGUGAAGAAUUCGACCACCU | AGGACAAA | -0.519 |
| Tetracycline | AAAACAUACCAGAUUUCGAUCUGGAGAGGUGAAGAAUUCGACCACCU | AGGGUUAA | -0.519 |
| Tetracycline | AAAACAUACCAGAUUUCGAUCUGGAGAGGUGAAGAAUUCGACCACCU | AAAACAUACCAGAUUUCGAUCUGGAGAGGUGAAGAAUUCGACCACCU | -0.519 |
| Tetracycline | AAAACAUACCAGAUUUCGAUCUGGAGAGGUGAAGAAUUCGACCACCU | AGAGAGAA | -0.518 |
| Tetracycline | AAAACAUACCAGAUUUCGAUCUGGAGAGGUGAAGAAUUCGACCACCU | AGGGUAAA | -0.518 |
| Tetracycline | AAAACAUACCAGAUUUCGAUCUGGAGAGGUGAAGAAUUCGACCACCU | AGAAG | -0.518 |
| Tetracycline | AAAACAUACCAGAUUUCGAUCUGGAGAGGUGAAGAAUUCGACCACCU | AAAACAUACCAGAUUUCGAUCUGGAGAGGUGAAGAAUUCGACCACCU | -0.518 |
| Tetracycline | AAAACAUACCAGAUUUCGAUCUGGAGAGGUGAAGAAUUCGACCACCU | AAGAG | -0.518 |
| Tetracycline | AAAACAUACCAGAUUUCGAUCUGGAGAGGUGAAGAAUUCGACCACCU | AGGAUAAA | -0.517 |
| Tetracycline | AAAACAUACCAGAUUUCGAUCUGGAGAGGUGAAGAAUUCGACCACCU | AGGAAUAA | -0.517 |
| Tetracycline | AAAACAUACCAGAUUUCGAUCUGGAGAGGUGAAGAAUUCGACCACCU | AAGAGGGA | -0.517 |
| Tetracycline | AAAACAUACCAGAUUUCGAUCUGGAGAGGUGAAGAAUUCGACCACCU | AGGCAAAA | -0.517 |
| Tetracycline | AAAACAUACCAGAUUUCGAUCUGGAGAGGUGAAGAAUUCGACCACCU | AGGGG | -0.517 |
| Tetracycline | AAAACAUACCAGAUUUCGAUCUGGAGAGGUGAAGAAUUCGACCACCU | AGGGCAAA | -0.516 |
| Tetracycline | AAAACAUACCAGAUUUCGAUCUGGAGAGGUGAAGAAUUCGACCACCU | AAAACAUACCAGAUUUCGAUCUGGAGAGGUGAAGAAUUCGACCACCU | -0.516 |
| Tetracycline | AAAACAUACCAGAUUUCGAUCUGGAGAGGUGAAGAAUUCGACCACCU | AUGGGGAA | -0.516 |
| Tetracycline | AAAACAUACCAGAUUUCGAUCUGGAGAGGUGAAGAAUUCGACCACCU | AGGAGUGA | -0.515 |
| Tetracycline | AAAACAUACCAGAUUUCGAUCUGGAGAGGUGAAGAAUUCGACCACCU | AGAGAGGA | -0.514 |
| Tetracycline | AAAACAUACCAGAUUUCGAUCUGGAGAGGUGAAGAAUUCGACCACCU | AGGUGAA | -0.514 |
| Tetracycline | AAAACAUACCAGAUUUCGAUCUGGAGAGGUGAAGAAUUCGACCACCU | AAAACAUACCAGAUUUCGAUCUGGAGAGGUGAAGAAUUCGACCACCU | -0.513 |
| Tetracycline | AAAACAUACCAGAUUUCGAUCUGGAGAGGUGAAGAAUUCGACCACCU | AGGCGAAA | -0.513 |
| Tetracycline | AAAACAUACCAGAUUUCGAUCUGGAGAGGUGAAGAAUUCGACCACCU | AAGGUGAA | -0.513 |
| Tetracycline | AAAACAUACCAGAUUUCGAUCUGGAGAGGUGAAGAAUUCGACCACCU | AUGAA | -0.513 |
| Tetracycline | AAAACAUACCAGAUUUCGAUCUGGAGAGGUGAAGAAUUCGACCACCU | AGGAAUGA | -0.512 |
| Tetracycline | AAAACAUACCAGAUUUCGAUCUGGAGAGGUGAAGAAUUCGACCACCU | AGAAAGAA | -0.512 |
| Tetracycline | AAAACAUACCAGAUUUCGAUCUGGAGAGGUGAAGAAUUCGACCACCU | AUGGAGGA | -0.512 |
| Tetracycline | AAAACAUACCAGAUUUCGAUCUGGAGAGGUGAAGAAUUCGACCACCU | AGGGUUGA | -0.511 |
| Tetracycline | AAAACAUACCAGAUUUCGAUCUGGAGAGGUGAAGAAUUCGACCACCU | AAAACAUACCAGAUUUCGAUCUGGAGAGGUGAAGAAUUCGACCACCU | -0.511 |
| Tetracycline | AAAACAUACCAGAUUUCGAUCUGGAGAGGUGAAGAAUUCGACCACCU | AGGCUGAA | -0.511 |
| Tetracycline | AAAACAUACCAGAUUUCGAUCUGGAGAGGUGAAGAAUUCGACCACCU | AGGGCUAA | -0.511 |
| Tetracycline | AAAACAUACCAGAUUUCGAUCUGGAGAGGUGAAGAAUUCGACCACCU | AGGUAAAA | -0.511 |
| Tetracycline | AAAACAUACCAGAUUUCGAUCUGGAGAGGUGAAGAAUUCGACCACCU | AAGGGUAA | -0.510 |
| Tetracycline | AAAACAUACCAGAUUUCGAUCUGGAGAGGUGAAGAAUUCGACCACCU | AUGGGGGA | -0.510 |
| Tetracycline | AAAACAUACCAGAUUUCGAUCUGGAGAGGUGAAGAAUUCGACCACCU | AAGGAAAA | -0.510 |
| Tetracycline | AAAACAUACCAGAUUUCGAUCUGGAGAGGUGAAGAAUUCGACCACCU | ACGAA | -0.510 |
| Tetracycline | AAAACAUACCAGAUUUCGAUCUGGAGAGGUGAAGAAUUCGACCACCU | AGGGUAGA | -0.510 |
| Tetracycline | AAAACAUACCAGAUUUCGAUCUGGAGAGGUGAAGAAUUCGACCACCU | AGGACAGA | -0.509 |
| Tetracycline | AAAACAUACCAGAUUUCGAUCUGGAGAGGUGAAGAAUUCGACCACCU | AAGGGAAA | -0.509 |
| Tetracycline | AAAACAUACCAGAUUUCGAUCUGGAGAGGUGAAGAAUUCGACCACCU | AGGGGCAA | -0.509 |
| Tetracycline | AAAACAUACCAGAUUUCGAUCUGGAGAGGUGAAGAAUUCGACCACCU | AGGGACAA | -0.509 |
| Tetracycline | AAAACAUACCAGAUUUCGAUCUGGAGAGGUGAAGAAUUCGACCACCU | AGAAAGGA | -0.509 |
| Tetracycline | AAAACAUACCAGAUUUCGAUCUGGAGAGGUGAAGAAUUCGACCACCU | AGGCGAA | -0.509 |
| Tetracycline | AAAACAUACCAGAUUUCGAUCUGGAGAGGUGAAGAAUUCGACCACCU | UGGGG | -0.509 |
| Tetracycline | AAAACAUACCAGAUUUCGAUCUGGAGAGGUGAAGAAUUCGACCACCU | AAGAUGAA | -0.509 |
| Tetracycline | AAAACAUACCAGAUUUCGAUCUGGAGAGGUGAAGAAUUCGACCACCU | AAGGGUGA | -0.509 |
| Tetracycline | AAAACAUACCAGAUUUCGAUCUGGAGAGGUGAAGAAUUCGACCACCU | AGGCAAGA | -0.508 |
| Tetracycline | AAAACAUACCAGAUUUCGAUCUGGAGAGGUGAAGAAUUCGACCACCU | AGGAUAGA | -0.508 |
| Tetracycline | AAAACAUACCAGAUUUCGAUCUGGAGAGGUGAAGAAUUCGACCACCU | AAAACAUACCAGAUUUCGAUCUGGAGAGGUGAAGAAUUCGACCACCU | -0.508 |
| Tetracycline | AAAACAUACCAGAUUUCGAUCUGGAGAGGUGAAGAAUUCGACCACCU | AAGCAGAA | -0.508 |
| Tetracycline | AAAACAUACCAGAUUUCGAUCUGGAGAGGUGAAGAAUUCGACCACCU | AUGAAGAA | -0.508 |
| Tetracycline | AAAACAUACCAGAUUUCGAUCUGGAGAGGUGAAGAAUUCGACCACCU | AAAACAUACCAGAUUUCGAUCUGGAGAGGUGAAGAAUUCGACCACCU | -0.507 |
| Tetracycline | AAAACAUACCAGAUUUCGAUCUGGAGAGGUGAAGAAUUCGACCACCU | AGAGGUAA | -0.507 |
| Tetracycline | AAAACAUACCAGAUUUCGAUCUGGAGAGGUGAAGAAUUCGACCACCU | AAGAAAAA | -0.507 |
| Tetracycline | AAAACAUACCAGAUUUCGAUCUGGAGAGGUGAAGAAUUCGACCACCU | AAAACAUACCAGAUUUCGAUCUGGAGAGGUGAAGAAUUCGACCACCU | -0.507 |
| Tetracycline | AAAACAUACCAGAUUUCGAUCUGGAGAGGUGAAGAAUUCGACCACCU | AUGAGGAA | -0.507 |
| Tetracycline | AAAACAUACCAGAUUUCGAUCUGGAGAGGUGAAGAAUUCGACCACCU | AAGUAGAA | -0.506 |
| Tetracycline | AAAACAUACCAGAUUUCGAUCUGGAGAGGUGAAGAAUUCGACCACCU | AGAGGUGA | -0.506 |
| Tetracycline | AAAACAUACCAGAUUUCGAUCUGGAGAGGUGAAGAAUUCGACCACCU | AAGGAUAA | -0.506 |
| Tetracycline | AAAACAUACCAGAUUUCGAUCUGGAGAGGUGAAGAAUUCGACCACCU | AAGGAA | -0.506 |
| Tetracycline | AAAACAUACCAGAUUUCGAUCUGGAGAGGUGAAGAAUUCGACCACCU | AAGAGAAA | -0.506 |
| Tetracycline | AAAACAUACCAGAUUUCGAUCUGGAGAGGUGAAGAAUUCGACCACCU | AAGGUGGA | -0.506 |
| Tetracycline | AAAACAUACCAGAUUUCGAUCUGGAGAGGUGAAGAAUUCGACCACCU | AGGCGAGA | -0.506 |
| Tetracycline | AAAACAUACCAGAUUUCGAUCUGGAGAGGUGAAGAAUUCGACCACCU | AGGUGAAA | -0.506 |
| Tetracycline | AAAACAUACCAGAUUUCGAUCUGGAGAGGUGAAGAAUUCGACCACCU | AGGUUGAA | -0.506 |
| Tetracycline | AAAACAUACCAGAUUUCGAUCUGGAGAGGUGAAGAAUUCGACCACCU | AAGGAUGA | -0.505 |
| Tetracycline | AAAACAUACCAGAUUUCGAUCUGGAGAGGUGAAGAAUUCGACCACCU | AAAACAUACCAGAUUUCGAUCUGGAGAGGUGAAGAAUUCGACCACCU | -0.505 |
| Tetracycline | AAAACAUACCAGAUUUCGAUCUGGAGAGGUGAAGAAUUCGACCACCU | AAGACGAA | -0.505 |
| Tetracycline | AAAACAUACCAGAUUUCGAUCUGGAGAGGUGAAGAAUUCGACCACCU | AAAACAUACCAGAUUUCGAUCUGGAGAGGUGAAGAAUUCGACCACCU | -0.505 |
| Tetracycline | AAAACAUACCAGAUUUCGAUCUGGAGAGGUGAAGAAUUCGACCACCU | AGGCAUAA | -0.505 |
| Tetracycline | AAAACAUACCAGAUUUCGAUCUGGAGAGGUGAAGAAUUCGACCACCU | AGGGCAGA | -0.505 |
| Tetracycline | AAAACAUACCAGAUUUCGAUCUGGAGAGGUGAAGAAUUCGACCACCU | AAAACAUACCAGAUUUCGAUCUGGAGAGGUGAAGAAUUCGACCACCU | -0.505 |
| Tetracycline | AAAACAUACCAGAUUUCGAUCUGGAGAGGUGAAGAAUUCGACCACCU | AAAACAUACCAGAUUUCGAUCUGGAGAGGUGAAGAAUUCGACCACCU | -0.504 |
| Tetracycline | AAAACAUACCAGAUUUCGAUCUGGAGAGGUGAAGAAUUCGACCACCU | AGUGAGGA | -0.504 |
| Tetracycline | AAAACAUACCAGAUUUCGAUCUGGAGAGGUGAAGAAUUCGACCACCU | AGAGAUGA | -0.504 |
| Tetracycline | AAAACAUACCAGAUUUCGAUCUGGAGAGGUGAAGAAUUCGACCACCU | AGUAA | -0.504 |
| Tetracycline | AAAACAUACCAGAUUUCGAUCUGGAGAGGUGAAGAAUUCGACCACCU | AGGUA | -0.504 |
| Tetracycline | AAAACAUACCAGAUUUCGAUCUGGAGAGGUGAAGAAUUCGACCACCU | ACGGGGAA | -0.504 |
| Tetracycline | AAAACAUACCAGAUUUCGAUCUGGAGAGGUGAAGAAUUCGACCACCU | AGUGGGGA | -0.504 |
| Tetracycline | AAAACAUACCAGAUUUCGAUCUGGAGAGGUGAAGAAUUCGACCACCU | AGGCGUAA | -0.504 |
| Tetracycline | AAAACAUACCAGAUUUCGAUCUGGAGAGGUGAAGAAUUCGACCACCU | AAAAA | -0.504 |
| Tetracycline | AAAACAUACCAGAUUUCGAUCUGGAGAGGUGAAGAAUUCGACCACCU | AGAGAUAA | -0.504 |
| Tetracycline | AAAACAUACCAGAUUUCGAUCUGGAGAGGUGAAGAAUUCGACCACCU | AGGCUAAA | -0.504 |
| Tetracycline | AAAACAUACCAGAUUUCGAUCUGGAGAGGUGAAGAAUUCGACCACCU | ACGGAGAA | -0.503 |
| Tetracycline | AAAACAUACCAGAUUUCGAUCUGGAGAGGUGAAGAAUUCGACCACCU | AAAACAUACCAGAUUUCGAUCUGGAGAGGUGAAGAAUUCGACCACCU | -0.503 |
| Tetracycline | AAAACAUACCAGAUUUCGAUCUGGAGAGGUGAAGAAUUCGACCACCU | AAAACAUACCAGAUUUCGAUCUGGAGAGGUGAAGAAUUCGACCACCU | -0.503 |
| Tetracycline | AAAACAUACCAGAUUUCGAUCUGGAGAGGUGAAGAAUUCGACCACCU | AGGUAUAA | -0.502 |
| Tetracycline | AAAACAUACCAGAUUUCGAUCUGGAGAGGUGAAGAAUUCGACCACCU | AAGGGA | -0.502 |
| Tetracycline | AAAACAUACCAGAUUUCGAUCUGGAGAGGUGAAGAAUUCGACCACCU | AGGUCGAA | -0.502 |
| Tetracycline | AAAACAUACCAGAUUUCGAUCUGGAGAGGUGAAGAAUUCGACCACCU | AGGUUGGA | -0.502 |
| Tetracycline | AAAACAUACCAGAUUUCGAUCUGGAGAGGUGAAGAAUUCGACCACCU | AUGAGGGA | -0.502 |
| Tetracycline | AAAACAUACCAGAUUUCGAUCUGGAGAGGUGAAGAAUUCGACCACCU | AGGUGUAA | -0.502 |
| Tetracycline | AAAACAUACCAGAUUUCGAUCUGGAGAGGUGAAGAAUUCGACCACCU | AAGCAGGA | -0.501 |
| Tetracycline | AAAACAUACCAGAUUUCGAUCUGGAGAGGUGAAGAAUUCGACCACCU | AAGGCGAA | -0.501 |
| Tetracycline | AAAACAUACCAGAUUUCGAUCUGGAGAGGUGAAGAAUUCGACCACCU | AGGAGAA | -0.501 |
| Tetracycline | AAAACAUACCAGAUUUCGAUCUGGAGAGGUGAAGAAUUCGACCACCU | AGGUAAGA | -0.501 |
| Tetracycline | AAAACAUACCAGAUUUCGAUCUGGAGAGGUGAAGAAUUCGACCACCU | AGGGCUGA | -0.501 |
| Tetracycline | AAAACAUACCAGAUUUCGAUCUGGAGAGGUGAAGAAUUCGACCACCU | AAAACAUACCAGAUUUCGAUCUGGAGAGGUGAAGAAUUCGACCACCU | -0.501 |
| Tetracycline | AAAACAUACCAGAUUUCGAUCUGGAGAGGUGAAGAAUUCGACCACCU | AAGAUGGA | -0.501 |
| Tetracycline | AAAACAUACCAGAUUUCGAUCUGGAGAGGUGAAGAAUUCGACCACCU | AGGCAUGA | -0.500 |
| Tetracycline | AAAACAUACCAGAUUUCGAUCUGGAGAGGUGAAGAAUUCGACCACCU | AAGGGAGA | -0.500 |
| Tetracycline | AAAACAUACCAGAUUUCGAUCUGGAGAGGUGAAGAAUUCGACCACCU | AGGGGCGA | -0.500 |
| Tetracycline | AAAACAUACCAGAUUUCGAUCUGGAGAGGUGAAGAAUUCGACCACCU | AGAGGAAA | -0.500 |
| Tetracycline | AAAACAUACCAGAUUUCGAUCUGGAGAGGUGAAGAAUUCGACCACCU | AAAACAUACCAGAUUUCGAUCUGGAGAGGUGAAGAAUUCGACCACCU | -0.499 |
| Tetracycline | AAAACAUACCAGAUUUCGAUCUGGAGAGGUGAAGAAUUCGACCACCU | AGGACUAA | -0.499 |
| Tetracycline | AAAACAUACCAGAUUUCGAUCUGGAGAGGUGAAGAAUUCGACCACCU | AGGAUUAA | -0.499 |
| Tetracycline | AAAACAUACCAGAUUUCGAUCUGGAGAGGUGAAGAAUUCGACCACCU | AAGUAGGA | -0.499 |
| Tetracycline | AAAACAUACCAGAUUUCGAUCUGGAGAGGUGAAGAAUUCGACCACCU | AAAACAUACCAGAUUUCGAUCUGGAGAGGUGAAGAAUUCGACCACCU | -0.499 |
| Tetracycline | AAAACAUACCAGAUUUCGAUCUGGAGAGGUGAAGAAUUCGACCACCU | AGUGGGAA | -0.499 |
| Tetracycline | AAAACAUACCAGAUUUCGAUCUGGAGAGGUGAAGAAUUCGACCACCU | AGUGAGAA | -0.499 |
| Tetracycline | AAAACAUACCAGAUUUCGAUCUGGAGAGGUGAAGAAUUCGACCACCU | AAGACGGA | -0.499 |
| Tetracycline | AAAACAUACCAGAUUUCGAUCUGGAGAGGUGAAGAAUUCGACCACCU | AAAACAUACCAGAUUUCGAUCUGGAGAGGUGAAGAAUUCGACCACCU | -0.498 |
| Tetracycline | AAAACAUACCAGAUUUCGAUCUGGAGAGGUGAAGAAUUCGACCACCU | AGACAGAA | -0.498 |
| Tetracycline | AAAACAUACCAGAUUUCGAUCUGGAGAGGUGAAGAAUUCGACCACCU | AAGGAAGA | -0.498 |
| Tetracycline | AAAACAUACCAGAUUUCGAUCUGGAGAGGUGAAGAAUUCGACCACCU | AGGUGAGA | -0.498 |
| Tetracycline | AAAACAUACCAGAUUUCGAUCUGGAGAGGUGAAGAAUUCGACCACCU | AAAACAUACCAGAUUUCGAUCUGGAGAGGUGAAGAAUUCGACCACCU | -0.498 |
| Tetracycline | AAAACAUACCAGAUUUCGAUCUGGAGAGGUGAAGAAUUCGACCACCU | AAAACAUACCAGAUUUCGAUCUGGAGAGGUGAAGAAUUCGACCACCU | -0.498 |
| Tetracycline | AAAACAUACCAGAUUUCGAUCUGGAGAGGUGAAGAAUUCGACCACCU | AAAACAUACCAGAUUUCGAUCUGGAGAGGUGAAGAAUUCGACCACCU | -0.498 |
| Tetracycline | AAAACAUACCAGAUUUCGAUCUGGAGAGGUGAAGAAUUCGACCACCU | AGGCCAAA | -0.498 |
| Tetracycline | AAAACAUACCAGAUUUCGAUCUGGAGAGGUGAAGAAUUCGACCACCU | AAAACAUACCAGAUUUCGAUCUGGAGAGGUGAAGAAUUCGACCACCU | -0.498 |
| Tetracycline | AAAACAUACCAGAUUUCGAUCUGGAGAGGUGAAGAAUUCGACCACCU | AGGCGUGA | -0.497 |
| Tetracycline | AAAACAUACCAGAUUUCGAUCUGGAGAGGUGAAGAAUUCGACCACCU | AAAACAUACCAGAUUUCGAUCUGGAGAGGUGAAGAAUUCGACCACCU | -0.497 |
| Tetracycline | AAAACAUACCAGAUUUCGAUCUGGAGAGGUGAAGAAUUCGACCACCU | AGCGAGAA | -0.497 |
| Tetracycline | AAAACAUACCAGAUUUCGAUCUGGAGAGGUGAAGAAUUCGACCACCU | AGGUCGGA | -0.497 |
| Tetracycline | AAAACAUACCAGAUUUCGAUCUGGAGAGGUGAAGAAUUCGACCACCU | AGGUAUGA | -0.497 |
| Tetracycline | AAAACAUACCAGAUUUCGAUCUGGAGAGGUGAAGAAUUCGACCACCU | AGAAAUGA | -0.497 |
| Tetracycline | AAAACAUACCAGAUUUCGAUCUGGAGAGGUGAAGAAUUCGACCACCU | AAAACAUACCAGAUUUCGAUCUGGAGAGGUGAAGAAUUCGACCACCU | -0.497 |
| Tetracycline | AAAACAUACCAGAUUUCGAUCUGGAGAGGUGAAGAAUUCGACCACCU | UAGGG | -0.496 |
| Tetracycline | AAAACAUACCAGAUUUCGAUCUGGAGAGGUGAAGAAUUCGACCACCU | CAGAA | -0.496 |
| Tetracycline | AAAACAUACCAGAUUUCGAUCUGGAGAGGUGAAGAAUUCGACCACCU | AAAACAUACCAGAUUUCGAUCUGGAGAGGUGAAGAAUUCGACCACCU | -0.496 |
| Tetracycline | AAAACAUACCAGAUUUCGAUCUGGAGAGGUGAAGAAUUCGACCACCU | AAGCGGAA | -0.496 |
| Tetracycline | AAAACAUACCAGAUUUCGAUCUGGAGAGGUGAAGAAUUCGACCACCU | AGGCUAGA | -0.496 |
| Tetracycline | AAAACAUACCAGAUUUCGAUCUGGAGAGGUGAAGAAUUCGACCACCU | AAAACAUACCAGAUUUCGAUCUGGAGAGGUGAAGAAUUCGACCACCU | -0.496 |
| Tetracycline | AAAACAUACCAGAUUUCGAUCUGGAGAGGUGAAGAAUUCGACCACCU | AGAGAAAA | -0.496 |
| Tetracycline | AAAACAUACCAGAUUUCGAUCUGGAGAGGUGAAGAAUUCGACCACCU | AAGAGUAA | -0.496 |
| Tetracycline | AAAACAUACCAGAUUUCGAUCUGGAGAGGUGAAGAAUUCGACCACCU | AAGGGAA | -0.496 |
| Tetracycline | AAAACAUACCAGAUUUCGAUCUGGAGAGGUGAAGAAUUCGACCACCU | AUGGA | -0.496 |
| Tetracycline | AAAACAUACCAGAUUUCGAUCUGGAGAGGUGAAGAAUUCGACCACCU | AGGGACGA | -0.496 |
| Tetracycline | AAAACAUACCAGAUUUCGAUCUGGAGAGGUGAAGAAUUCGACCACCU | AUGGUGAA | -0.496 |
| Tetracycline | AAAACAUACCAGAUUUCGAUCUGGAGAGGUGAAGAAUUCGACCACCU | AUGGGUAA | -0.496 |
| Tetracycline | AAAACAUACCAGAUUUCGAUCUGGAGAGGUGAAGAAUUCGACCACCU | AAAACAUACCAGAUUUCGAUCUGGAGAGGUGAAGAAUUCGACCACCU | -0.496 |
| Tetracycline | AAAACAUACCAGAUUUCGAUCUGGAGAGGUGAAGAAUUCGACCACCU | UAGAG | -0.495 |
| Tetracycline | AAAACAUACCAGAUUUCGAUCUGGAGAGGUGAAGAAUUCGACCACCU | AAAACAUACCAGAUUUCGAUCUGGAGAGGUGAAGAAUUCGACCACCU | -0.495 |
| Tetracycline | AAAACAUACCAGAUUUCGAUCUGGAGAGGUGAAGAAUUCGACCACCU | AGACAGGA | -0.495 |
| Tetracycline | AAAACAUACCAGAUUUCGAUCUGGAGAGGUGAAGAAUUCGACCACCU | AGGGUCAA | -0.495 |
| Tetracycline | AAAACAUACCAGAUUUCGAUCUGGAGAGGUGAAGAAUUCGACCACCU | AAAACAUACCAGAUUUCGAUCUGGAGAGGUGAAGAAUUCGACCACCU | -0.495 |
| Tetracycline | AAAACAUACCAGAUUUCGAUCUGGAGAGGUGAAGAAUUCGACCACCU | AGGAGA | -0.495 |
| Tetracycline | AAAACAUACCAGAUUUCGAUCUGGAGAGGUGAAGAAUUCGACCACCU | AGGAGCAA | -0.494 |
| Tetracycline | AAAACAUACCAGAUUUCGAUCUGGAGAGGUGAAGAAUUCGACCACCU | AGUGA | -0.494 |
| Tetracycline | AAAACAUACCAGAUUUCGAUCUGGAGAGGUGAAGAAUUCGACCACCU | AAAACAUACCAGAUUUCGAUCUGGAGAGGUGAAGAAUUCGACCACCU | -0.494 |
| Tetracycline | AAAACAUACCAGAUUUCGAUCUGGAGAGGUGAAGAAUUCGACCACCU | AAAGA | -0.494 |
| Tetracycline | AAAACAUACCAGAUUUCGAUCUGGAGAGGUGAAGAAUUCGACCACCU | AAAACAUACCAGAUUUCGAUCUGGAGAGGUGAAGAAUUCGACCACCU | -0.494 |
| Tetracycline | AAAACAUACCAGAUUUCGAUCUGGAGAGGUGAAGAAUUCGACCACCU | ACGAAGAA | -0.494 |
| Tetracycline | AAAACAUACCAGAUUUCGAUCUGGAGAGGUGAAGAAUUCGACCACCU | ACGAGGAA | -0.494 |
